# Machine-learning phenotyping and exploratory medication-outcome associations in heart failure with preserved ejection fraction: a multicohort retrospective study

**DOI:** 10.3389/fphar.2026.1838338

**Published:** 2026-09-28

**Authors:** Qianli Ma, Shaotong Zhang, Chengjian Guan, Yitong Liu, Xiuchun Yang, Xiong Chen, Sheng Jin, Bing Xiao

**Affiliations:** 1 Department of Cardiology, The Second Hospital of Hebei Medical University, Shijiazhuang, China; 2 Medical Technology College, Hebei Medical University, Shijiazhuang, China; 3 Department of Urology, Sun Yat-sen Memorial Hospital, Sun Yat-sen University, Guangzhou, China; 4 Department of Physiology, Hebei Medical University, Shijiazhuang, China; 5 The Key Laboratory of Neural and Vascular Biology, Ministry of Education, Shijiazhuang, China; 6 Hebei Key Laboratory of Cardiovascular Homeostasis and Aging, Shijiazhuang, China

**Keywords:** HFpEF, medication–mortality associations, phenotyping, TabPFN classifier, unsupervised machine learning

## Abstract

**Background:**

The marked clinical and pathophysiological heterogeneity of HFpEF has repeatedly undermined uniform treatment strategies. Consequently, identifying distinct phenotypes and medication-associated patterns across phenotypes may inform future precision-medicine research.

**Methods:**

In this multicohort retrospective study, patients with HFpEF were included from MIMIC-IV (derivation cohort, n = 2,511), the MIMIC-III CareVue subset (internal validation cohort, n = 1,524), and the Second Hospital of Hebei Medical University (external application cohort, n = 340). Unsupervised K-prototypes clustering was used to derive phenotypic groups, with K = 2 evaluated as a lower-resolution sensitivity solution. All phenotype-stratified medication analyses were exploratory. In the derivation and internal validation cohorts, overlap weighting was the primary weighted analysis and stabilized inverse probability of treatment weighting was a sensitivity analysis; external medication estimates were reported descriptively because adequate within-group covariate balance was not achieved. A TabPFN phenotype-assignment classifier was developed after recursive feature elimination, evaluated in internal validation against independently reclustered labels aligned by prespecified one-to-one Hungarian assignment on cluster-profile distances, and applied without refitting in the external cohort.

**Results:**

The primary three-cluster solution identified clinically interpretable but partially overlapping cardiorenal-metabolic, hypertensive-pulmonary, and low-blood-pressure/arrhythmia profiles. K = 2 had a higher mean silhouette width than K = 3 (0.083 versus 0.060), whereas both solutions showed high median resampling stability (ARI, 0.940 versus 0.924). A graded 365-day mortality difference was observed across the three clusters in the derivation cohort, but between-cluster survival differences were not statistically significant in the internal validation or external application cohorts. In the derivation and internal validation cohorts, no weighted cluster-specific medication association remained statistically significant after FDR correction, and all medication-by-cluster interaction tests were nonsignificant. External medication estimates were descriptive and were not used for confirmatory inference. The fixed TabPFN classifier achieved one-versus-rest AUCs of 0.951–0.969 in internal validation (10-bin multiclass ECE = 0.028).

**Conclusion:**

Routinely collected ICU data supported an exploratory framework for describing overlapping HFpEF phenotypes and reproducibly assigning machine-derived labels. The analyses did not establish phenotype-specific medication effects, and the classifier should be regarded as a research tool pending prospective multicenter validation.

## 1 Introduction

Heart failure with preserved ejection fraction (HFpEF) has emerged as a major contributor to the global burden of heart failure, especially among older adults (Campbell et al., 2024). Contemporary epidemiological reviews indicate a growing heart-failure burden across the left ventricular ejection-fraction spectrum, with substantial hospitalization and mortality associated with HFpEF (Campbell et al., 2024; Savarese et al., 2023).

The management of HFpEF is particularly challenging, primarily owing to its profound clinical and biological heterogeneity (Borlaug et al., 2023; Vilaro, 2023). Affected patients are typically elderly and frequently present with multiple coexisting comorbidities, including hypertension, atrial fibrillation, diabetes, obesity, and chronic kidney disease, which interact synergistically to create intricate and overlapping pathophysiological mechanisms (Redfield, 2016). Although sodium-glucose cotransporter 2 (SGLT2) inhibitors have improved clinical outcomes in HFpEF, treatment selection remains individualized, particularly for therapies used to manage congestion and coexisting cardiovascular conditions (Wei and Du, 2023; Metra et al., 2025; Vaduganathan et al., 2022). Evidence and guideline recommendations for renin–angiotensin system inhibitors, mineralocorticoid receptor antagonists, and beta-blockers in HFpEF differ by drug class, ejection-fraction range, and treatment indication (Heidenreich et al., 2022; Zafeiropoulos et al., 2024; Solomon et al., 2024; Peikert et al., 2024). This heterogeneity has motivated efforts to identify clinically meaningful phenotypes that may improve risk stratification and generate hypotheses for future phenotype-informed therapeutic studies.

Unsupervised machine learning has therefore gained increasing attention as a useful approach for examining HFpEF heterogeneity. By identifying patterns in routinely collected clinical variables, unsupervised clustering may reveal latent phenotypes that support patient stratification. For example, Segar et al. applied unsupervised clustering to heterogeneous clinical variables in the TOPCAT cohort, revealing three distinct phenogroups: a multimorbid high-risk phenotype, a diastolic dysfunction phenotype, and a low-risk phenotype, thereby underscoring the effectiveness of this analytic strategy (Segar et al., 2020). However, most previous studies have focused primarily on phenotype identification and prognosis. Whether associations between early exposure to commonly used medications and 365-day mortality are consistent across data-driven HFpEF phenotypes remains uncertain. Therefore, the present study aimed to derive exploratory ICU-based HFpEF phenotypes, evaluate their reproducibility in an internal validation cohort, assess the transportability of classifier-assigned phenotypes in an external application cohort, and explore phenotype-stratified associations of early exposure to diuretics, ACEI/ARB, and beta-blockers with 365-day mortality.

This multicohort study integrated clinical data from a derivation cohort, an internal validation cohort, and an external application cohort. We applied K-prototypes clustering to identify exploratory HFpEF phenotypes and evaluated cluster-stratified associations between early medication exposure and 365-day mortality using Cox proportional hazards models. All medication–phenotype analyses were exploratory and were not designed to establish treatment effects. Finally, we developed a TabPFN-based research phenotype-assignment model to facilitate standardized assignment of the machine-derived labels in future studies.

## 2 Materials and methods

### 2.1 Data sources, cohort construction, and eligibility criteria

The study included a derivation cohort, an internal validation cohort, and an external application cohort. The derivation cohort was sourced from the publicly available Medical Information Mart for Intensive Care (MIMIC)-IV database (version 2.2). The internal validation cohort was derived from the CareVue subset of the MIMIC-III database (version 1.4), which was selected because CareVue data are distinct from the MetaVision data included in MIMIC-IV. The external application cohort consisted of patients with HFpEF admitted to the intensive care unit of the Second Hospital of Hebei Medical University between January 2022 and September 2024. Data harmonization procedures, including variable definitions, unit conversion, and outcome ascertainment, were applied consistently across cohorts. Use of the external cohort was approved by the Research Ethics Committee of the Second Hospital of Hebei Medical University (Approval Letter No. 2025-R048, 20 February 2025) (Figure 1). The MIMIC-IV and MIMIC-III data resources have been described in detail elsewhere (Johnson et al., 2023; Johnson et al., 2016).

**FIGURE 1:**
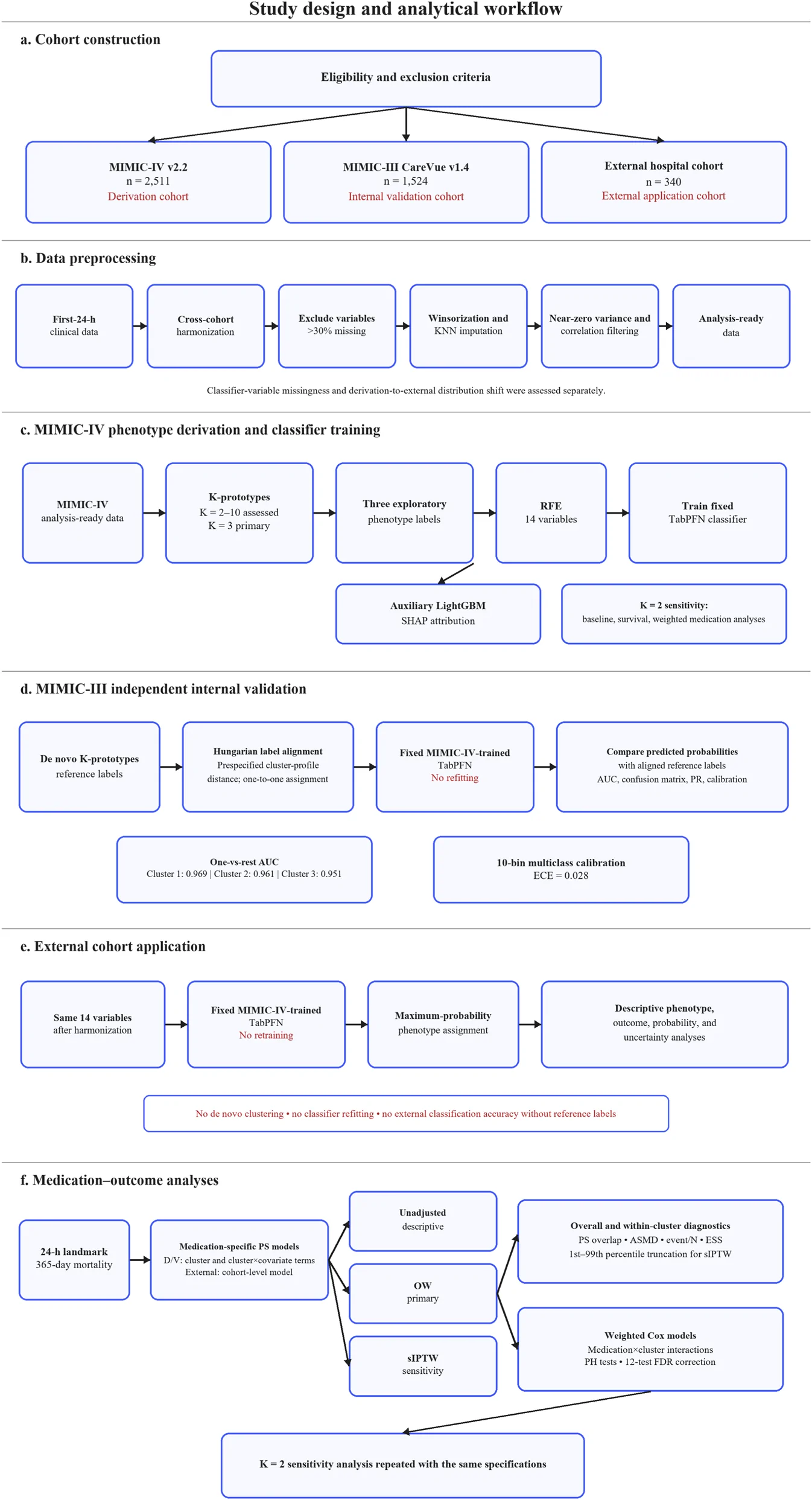
Schematic illustration of the study design and analytical workflow. Abbreviations: ACEI, angiotensin-converting enzyme inhibitor; ARB, angiotensin receptor blocker; AUC, area under the receiver operating characteristic curve; FDR, false discovery rate; KNN, k-nearest neighbors; LightGBM, Light Gradient Boosting Machine; MIMIC, Medical Information Mart for Intensive Care; OW, overlap weighting; RFE, recursive feature elimination; SHAP, SHapley Additive exPlanations; sIPTW, stabilized inverse probability of treatment weighting; TabPFN, Tabular Prior-Data Fitted Network.

The detailed patient selection process was as follows. First, patients hospitalized with heart failure were identified using International Classification of Diseases, Ninth/10th Revision (ICD-9/ICD-10) codes. The complete ICD-9-CM and ICD-10-CM code lists used for heart failure identification and prespecified disease exclusions are provided in Supplementary Table S1. Left ventricular ejection fraction (LVEF) values were extracted from echocardiography reports documented in the electronic medical record. Patients were eligible if they had an LVEF of at least 50% and met the prespecified heart failure coding criteria. For the MIMIC-IV derivation cohort, records were retained only when they represented both the patient’s first hospital stay and first ICU stay. Patients were excluded if they met any of the following criteria: (1) LVEF <50% or missing LVEF data; (2) concomitant diagnoses of infiltrative/restrictive cardiomyopathy, hypertrophic cardiomyopathy, acute myocardial infarction, congenital heart disease, constrictive pericarditis, or history of heart transplantation; (3) age <18 years; and (4) ICU stay shorter than 24 h. Comparator-qualified expressions were interpreted conservatively: upper-bound statements were not accepted as evidence of LVEF ≥50%, whereas exact values and lower-bound statements were eligible only when the stated threshold was at least 50%.

### 2.2 Clinical variables and study endpoint

#### 2.2.1 Baseline data

Clinical variables used for phenotyping were collected during the first 24 h after ICU admission in all three cohorts. For repeatedly timestamped vital signs, laboratory measurements, blood-cell indices, and echocardiographic measurements, the first qualifying value recorded within 24 h after ICU admission was used. Weight was obtained from the official MIMIC first-day derived view. For medication-association analyses, variables from this early ICU window were treated as candidate covariates for confounding adjustment; the timing of covariate measurement relative to medication administration is further addressed in the medication-exposure and sensitivity analyses. Demographic and anthropometric characteristics included age, sex, and weight. Continuous vital signs were recorded, comprising heart rate (HR), systolic blood pressure (SBP), mean blood pressure (MBP), respiratory rate (RR), temperature, and peripheral oxygen saturation (SpO_2_). Cardiac function was assessed via ejection fraction (EF). Laboratory assessments covered hematological markers, specifically hematocrit, mean corpuscular hemoglobin concentration (MCHC), mean corpuscular volume (MCV), platelet count, red cell distribution width (RDW), and white blood cell count (WBC). Biochemical and electrolyte profiles included glucose, anion gap, blood urea nitrogen (BUN), calcium, chloride, creatinine, sodium, and potassium, while coagulation function was measured by prothrombin time (PT) and partial thromboplastin time (PTT). Comorbidities were identified based on ICD codes and included myocardial infarction, peripheral vascular disease, cerebrovascular disease, chronic pulmonary disease, diabetes, liver disease, renal disease, malignant cancer, hypertension, and cardiac arrhythmias. Mechanical ventilation during the first 24 h was also recorded. Laboratory units were harmonized across cohorts before analysis; the diagnosis-code lists used for cohort inclusion and exclusion are provided in Supplementary Table S1.

#### 2.2.2 Medication exposure

The index time was ICU admission. Medication exposure was assessed during the first 24 h after ICU admission. The three medication classes of interest were diuretics, angiotensin-converting enzyme inhibitors or angiotensin receptor blockers (ACEI/ARB), and beta-blockers. For each medication class, patients who received at least one administration during this period were classified as exposed. Patients could be exposed to more than one medication class; each medication class was analyzed separately. In the 24-h landmark analysis, patients who died before 24 h were excluded, and follow-up began 24 h after ICU admission.

#### 2.2.3 Study endpoint

The primary study endpoint was all-cause mortality within 365 days after ICU admission. For MIMIC-III and MIMIC-IV, dates of death were obtained from the mortality records available in the respective databases; for the external application cohort, vital status and dates of death were ascertained through telephone follow-up. For the 24-h landmark analysis, patients who died within the first 24 h after ICU admission were excluded, and follow-up began 24 h after ICU admission. Follow-up ended at death, 365 days after ICU admission, or the last available follow-up, whichever occurred first. Patients without an observed death during follow-up were censored at day 365 or their last available follow-up date.

### 2.3 Data processing and analysis

#### 2.3.1 Data preprocessing

A standardized preprocessing protocol was applied across cohorts. Variables with more than 30% missing values were excluded; missing-data proportions and patterns among retained variables are shown in Supplementary Figure S1. Before imputation, missingness was summarized separately by cohort for the 14 phenotype-classifier variables, and distribution differences between the derivation and external application cohorts were quantified using absolute standardized mean differences (Supplementary Table S6). Continuous variables were winsorized at the 1st and 99th percentiles before K-nearest-neighbor imputation with k = 10. Variables with near-zero variance were screened, and highly correlated variables were removed using a correlation threshold of 0.80.

#### 2.3.2 Unsupervised clustering and phenotype identification

Phenotypic clustering was performed using the K-prototypes algorithm. Prior to clustering, continuous variables were Z-score standardized and rescaled to the range [-1, 1], and categorical features were converted to factors. Candidate solutions from K = 2 to K = 10 were evaluated using the total within-cluster sum of squares and average silhouette width. For candidate solutions from K = 2 to K = 10, cluster stability was evaluated using 50 repeated subsampling iterations. In each iteration, 80% of patients were sampled without replacement, a K-prototypes model was refitted using 15 random initializations, and the resulting prototypes were used to assign cluster labels to the complete derivation cohort. Agreement between these reassigned labels and the corresponding full-sample solution was quantified using the adjusted Rand index (ARI), which was summarized using the median and interquartile range across iterations.

The cluster-number decision rule was specified before examination of the medication–outcome associations and integrated statistical separation, resampling stability, avoidance of very small clusters, and clinical interpretability based on baseline phenotypic profiles. Medication exposure and survival outcomes were not used as clustering inputs. The statistical indices did not identify K = 3 as a uniquely optimal solution: K = 2 showed modestly greater separation, whereas both solutions showed high resampling stability. K = 3 was retained as the main exploratory representation because it provided greater clinical granularity, while K = 2 was treated as an equally plausible lower-resolution sensitivity solution.

The final three-cluster solution was estimated with 100 maximum iterations and 25 random starts (random seed = 0406). Uniform Manifold Approximation and Projection (UMAP) was applied for two-dimensional visualization only. As a sensitivity analysis, the complete K-prototypes procedure was repeated with K = 2. Baseline phenotypic characteristics, 365-day survival patterns, and the principal propensity-score-weighted medication analyses were repeated under the two-cluster solution using the same variable definitions, medication exposures, outcomes, and analytical specifications as in the primary three-cluster analysis.

#### 2.3.3 Associations between early medication exposure and 365-day mortality across phenotypes

We explored associations between early exposure to diuretics, ACEI/ARB, or beta-blockers and 365-day mortality across the identified phenotypes using a 24-h landmark design. These medication–phenotype analyses were exploratory and were not intended to estimate causal treatment effects. Kaplan–Meier curves and unadjusted Cox proportional hazards models were used for descriptive analyses. For each medication in the derivation and internal validation cohorts, propensity scores were estimated using logistic regression including cluster membership, 14 prespecified covariates, and all cluster-by-covariate interaction terms. The covariates were age, sex, weight, heart rate, systolic blood pressure, peripheral oxygen saturation, creatinine, potassium, blood urea nitrogen, myocardial infarction, chronic pulmonary disease, renal disease, hypertension, and cardiac arrhythmias; creatinine and blood urea nitrogen were log-transformed in the propensity-score models. Because the fully interacted specification could not be estimated reliably in the smaller external cohort, cohort-level weighted estimates without cluster interactions were retained only as descriptive summaries alongside balance diagnostics and were not used for adjusted cluster-specific inference.

Within the exploratory derivation and internal validation analyses, overlap weighting was the primary weighted approach, and stabilized inverse probability of treatment weighting, truncated at the 1st and 99th percentiles, was used as a sensitivity analysis. Covariate balance was evaluated both overall and within each cluster using absolute standardized mean differences, with values below 0.10 considered acceptable. Propensity-score overlap, exposed and unexposed sample sizes, mortality-event counts, and effective sample sizes were assessed within each medication–cluster–cohort combination. Formal weighted cluster-specific associations and medication-by-cluster interactions were interpreted only in the derivation and internal validation cohorts. The proportional-hazards assumption was assessed using scaled Schoenfeld residuals. In these two cohorts, diagnostics were computed from the full medication-by-cluster Cox model using the same survival time, event indicator, complete-case analysis set, and analytical weights as the corresponding analysis; term-level tests were reported for the medication main effect, cluster main effect, and medication-by-cluster interaction, together with a global test (Supplementary Table S9). Within each cohort and analytical model, the Benjamini–Hochberg procedure was applied to the 12 medication-coefficient tests; interaction *P* values were reported separately. Additional details are provided in the Supplementary Methods. These weighting and diagnostic choices follow established guidance for propensity-score-weighted observational analyses (Desai and Franklin, 2019).

#### 2.3.4 Phenotype-assignment model development

A phenotype-assignment model was developed to reproduce the K-prototypes-derived phenotype labels rather than to predict mortality or treatment response. Recursive feature elimination using a random forest classifier, 10-fold cross-validation, and 100 bootstrap iterations evaluated candidate feature-set sizes of 8, 10, 12, 14, and 37 predictors. The 37-predictor model achieved the highest absolute cross-validated performance. Among the deliberately reduced-input configurations (8–14 predictors), the 14-predictor model achieved the best accuracy and Kappa. Because the intended research interface requires manual entry, we selected the 14-predictor configuration as a pragmatic performance–input-burden compromise; it is not presented as the globally performance-optimal subset. The final TabPFN v2.2.1 classifier was trained on the complete MIMIC-IV derivation cohort using these variables, with the derivation phenotype identities retained as the fixed supervised-learning target classes. The derivation sample size (n = 2,511) and the 14-feature input dimension were within the standard operating range of TabPFN v2.2.1; therefore, no additional row-wise subsampling was required. All benchmark models used the same 14 predictors, preprocessing steps, cross-validation folds, training/validation partitions, and evaluation metrics as TabPFN; only the learning algorithm and algorithm-specific hyperparameters differed. Feature attribution was examined using SHAP values from an auxiliary LightGBM classifier trained on the same features and phenotype labels. The model architecture follows the published TabPFN framework for small-to-medium tabular datasets (Hollmann et al., 2025).

Internal validation was performed in MIMIC-III by comparing predictions from the fixed MIMIC-IV-trained classifier with phenotype labels generated through independent *de novo* K-prototypes clustering. Before classifier evaluation, a one-to-one correspondence between the MIMIC-III clusters and MIMIC-IV phenotype identities was determined with the Hungarian algorithm by minimizing a prespecified cluster-profile distance across the clinical variables shared by the two clustering workflows. Continuous cluster summaries were standardized using derivation-cohort location and scale, categorical summaries were represented by cluster-specific prevalences, and feature contributions to the distance were equally weighted. The mapping was fixed before performance metrics were calculated; classifier predictions, survival outcomes, and medication exposures were not used to define the correspondence. The classifier was not refitted in MIMIC-III. Performance was evaluated using one-versus-rest AUCs, confusion matrices, calibration assessments, single-feature ablation, and representative-case analyses. In the external application cohort, where independently generated reference labels were unavailable, the fixed classifier was used for phenotype assignment and the resulting groups were evaluated descriptively rather than by classification accuracy. Detailed model-development and evaluation procedures are provided in the Supplementary Methods. The resulting web application was intended to support reproducible research phenotype assignment. The distinction among model development, internal validation, and external application is consistent with current prediction-model reporting principles (Collins et al., 2024).

#### 2.3.5 Statistical analysis

Continuous variables were summarized as median (interquartile range), and categorical variables were reported as frequency (percentage). Phenotypic clustering was performed using the K-prototypes algorithm, and survival outcomes were assessed using Kaplan–Meier curves and compared via the log-rank test. Hazard ratios and corresponding *P* values for medication associations were estimated using the Wald test from Cox proportional hazards models, applying robust sandwich variance estimators to account for propensity score weighting. OW was used as the primary confounding-adjustment approach, with sIPTW as a sensitivity analysis; stabilized weights were truncated at the 1st and 99th percentiles. Medication associations were reported as hazard ratios (HRs) with 95% confidence intervals (CIs). Model discrimination was evaluated using the one-versus-rest area under the receiver operating characteristic curve (AUC). Classification performance was additionally summarized using accuracy, precision, recall, F1 score, precision–recall curves, and confusion matrices. Probability calibration was evaluated using phenotype-specific Brier scores, calibration curves, and 10-bin multiclass ECE. All tests were two-sided, and subgroup and interaction analyses were considered exploratory because of multiple comparisons. Analyses were performed using R version 4.4.2 and Python version 3.11.

## 3 Results

### 3.1 Patient baseline characteristics

A total of 2,511 patients with HFpEF were included in the MIMIC-IV derivation cohort, of whom 768 (30.6%) died within 365 days after ICU admission. In the MIMIC-III internal validation cohort, 1,524 patients satisfied the inclusion criteria and 472 (31.0%) died within 365 days. The external application cohort consisted of 340 patients, among whom 127 (37.4%) experienced 365-day all-cause mortality.

### 3.2 Clustering results

Candidate K-prototypes solutions were compared using within-cluster sum of squares, average silhouette width, resampling stability, cluster-size distribution, and clinical interpretability. The two-cluster solution had a higher average silhouette width than the three-cluster solution (0.083 versus 0.060). Both solutions nevertheless showed high resampling stability: the median ARI was 0.940 (IQR, 0.260) for K = 2 and 0.924 (IQR, 0.175) for K = 3. Thus, the statistical indices did not demonstrate superiority of the three-cluster solution, and the low silhouette widths indicated partially overlapping rather than sharply separated phenotypes.

The three-cluster solution was retained as the main exploratory phenotyping representation because it separated three clinically recognizable baseline profiles, whereas the two-cluster solution combined these patterns into two broader groups. This choice reflected descriptive clinical granularity and did not establish statistical or biological superiority over K = 2. The K = 3 solution included 733 (29.2%), 922 (36.7%), and 856 (34.1%) patients and showed a graded pattern of observed 365-day mortality across Clusters 1–3 (38.3%, 31.5%, and 23.0%, respectively; *P* < 0.001). The complete K-selection and stability results and the concise K = 2 versus K = 3 interpretability assessment are presented in Supplementary Figure S2 and Supplementary Table S5. Cluster-defining characteristics for the main three-cluster solution are summarized in Figure 2 and Table 1.

**FIGURE 2:**
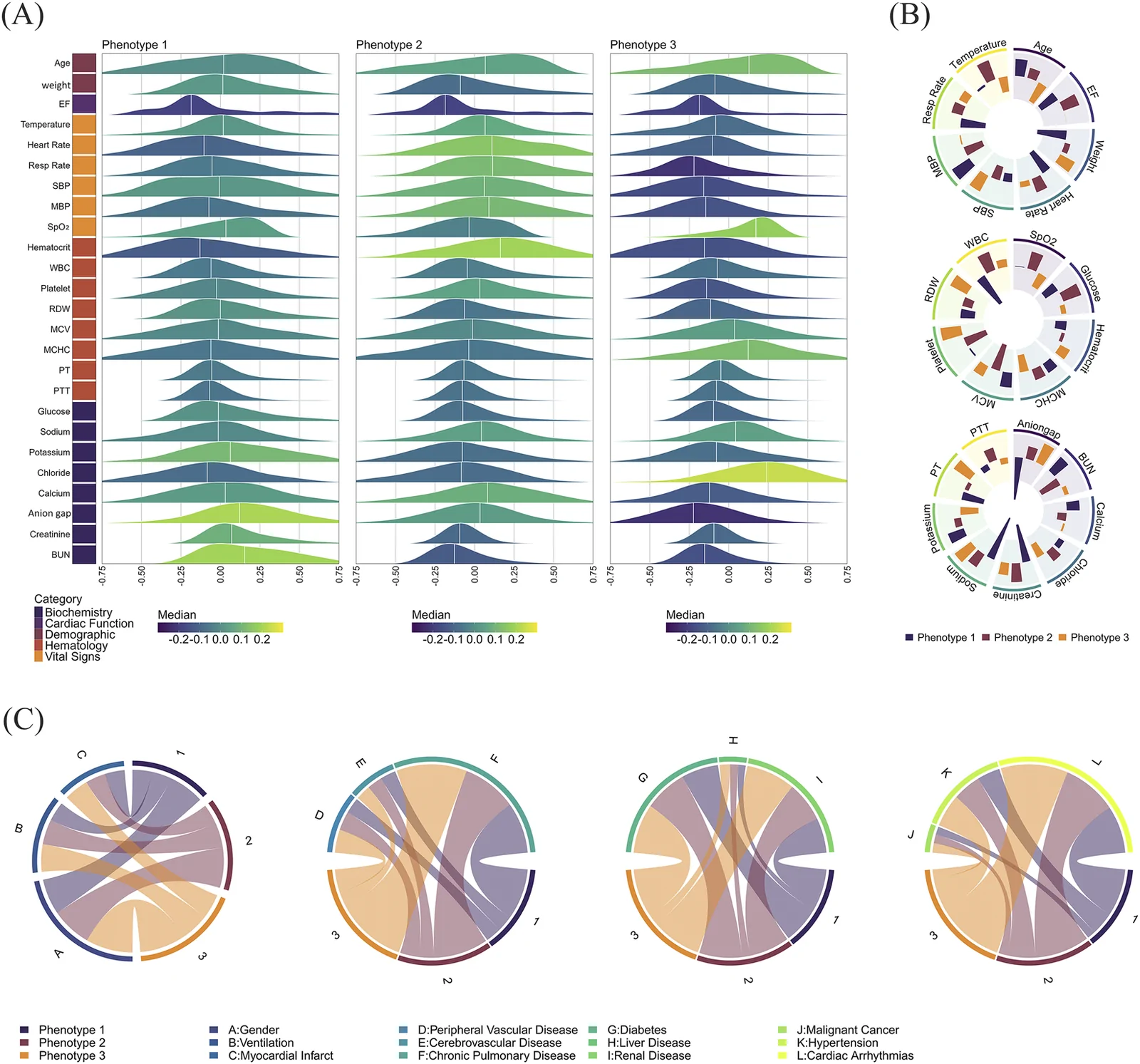
Clinical characteristics of the derivation cohort stratified by phenotypic clusters. **(A)** Mountain plots showing the distribution of continuous clinical variables across the three clusters. Variables are color-coded by clinical domain, as indicated on the left. **(B)** Circular bar plot displaying cluster-specific mean standardized values after rescaling to the range [−1, 1]. **(C)** Chord diagram visualizing associations between phenotypic clusters and key comorbidities. Abbreviations: AG, anion gap; BUN, blood urea nitrogen; EF, ejection fraction; HR, heart rate; MBP, mean blood pressure; MCHC, mean corpuscular hemoglobin concentration; MCV, mean corpuscular volume; PT, prothrombin time; PTT, partial thromboplastin time; RDW, red cell distribution width; RR, respiratory rate; SBP, systolic blood pressure; SpO_2_, peripheral oxygen saturation; WBC, white blood cell count.

**TABLE 1:** Baseline characteristics of the derivation cohort according to the primary three-cluster solution.

Variables	Phenotypes			*P*-value
	Cluster 1 (N = 733)	Cluster 2 (N = 922)	Cluster 3 (N = 856)	
Sex, n (%)	​	​	​	<0.001
Female	275 (38)	630 (68)	524 (61)	​
Male	458 (62)	292 (32)	332 (39)	​
Age, years	75.76 (65.89–83.62)	77.58 (67.39–85.94)	80.16 (70.65–86.54)	<0.001
EF, %	55.00 (55.00–60.00)	55.00 (55.00–65.00)	55.00 (55.00–55.00)	<0.001
Weight, kg	88.00 (73.50–105.50)	78.00 (64.20–99.50)	77.90 (65.15–93.55)	<0.001
HR, beats/min	80.00 (69.00–92.00)	94.00 (81.00–110.00)	80.00 (70.00–87.00)	<0.001
SBP, mmHg	128.00 (107.00–146.00)	133.00 (117.00–149.00)	115.00 (102.00–129.00)	<0.001
MBP, mmHg	78.00 (68.00–90.00)	88.00 (77.00–101.00)	74.00 (66.00–83.00)	<0.001
RR, breaths/min	19.00 (16.00–23.00)	22.00 (18.00–26.00)	16.00 (14.00–19.00)	<0.001
Temperature, °C	36.67 (36.39–37.00)	36.78 (36.50–37.11)	36.44 (36.06–36.78)	<0.001
SpO_2_, %	97.00 (95.00–100.00)	96.00 (93.00–98.00)	99.00 (97.00–100.00)	<0.001
Glucose, mg/dL	154.00 (113.00–212.00)	130.00 (108.00–168.00)	124.00 (104.00–153.00)	<0.001
Hematocrit, %	29.40 (26.10–33.60)	34.69 (30.70–38.50)	28.95 (25.30–32.47)	<0.001
MCHC, g/dL	32.10 (31.00–33.10)	32.10 (31.10–33.20)	32.90 (32.00–33.80)	<0.001
MCV, fL	91.00 (86.00–96.00)	91.00 (87.00–95.00)	92.00 (88.00–96.00)	0.004
Platelet, K/µL	199.00 (147.00–253.00)	220.00 (177.00–283.00)	161.50 (123.00–206.00)	<0.001
RDW, %	15.60 (14.50–17.10)	15.00 (14.00–16.50)	14.70 (13.70–15.90)	<0.001
WBC, K/µL	10.30 (7.60–13.60)	10.63 (7.90–14.70)	9.94 (7.40–13.74)	0.002
Anion gap, mEq/L	17.00 (15.00–19.00)	15.00 (13.00–17.00)	12.00 (11.00–14.00)	<0.001
BUN, mg/dL	49.00 (32.00–70.00)	23.00 (16.00–31.00)	21.00 (16.00–29.00)	<0.001
Calcium, mg/dL	8.50 (8.10–9.00)	8.60 (8.20–9.09)	8.20 (7.90–8.50)	<0.001
Chloride, mEq/L	101.00 (97.00–105.00)	101.00 (97.00–105.00)	107.00 (103.00–110.00)	<0.001
Creatinine, mg/dL	2.20 (1.60–3.60)	1.00 (0.80–1.30)	1.00 (0.80–1.30)	<0.001
Sodium, mEq/L	138.00 (135.00–140.00)	139.00 (136.00–141.00)	139.00 (137.00–141.00)	<0.001
Potassium, mEq/L	4.50 (4.10–5.00)	4.10 (3.70–4.50)	4.10 (3.80–4.50)	<0.001
PT, s	15.00 (12.80–18.66)	14.57 (12.81–18.04)	15.20 (13.40–17.40)	0.170
PTT, s	33.40 (28.70–42.30)	32.80 (28.20–41.68)	32.63 (28.40–40.02)	0.200
Ventilation, n (%)	​	​	​	<0.001
NO	578 (79)	763 (83)	535 (63)	​
YES	155 (21)	159 (17)	321 (38)	​
Myocardial infarction, n (%)	​	​	​	<0.001
NO	525 (72)	757 (82)	663 (77)	​
YES	208 (28)	165 (18)	193 (23)	​
Peripheral vascular disease, n (%)	​	​	​	0.023
NO	611 (83)	809 (88)	720 (84)	​
YES	122 (17)	113 (12)	136 (16)	​
Cerebrovascular disease, n (%)	​	​	​	0.900
NO	651 (89)	814 (88)	754 (88)	​
YES	82 (11)	108 (12)	102 (12)	​
Chronic pulmonary disease, n (%)	​	​	​	<0.001
NO	462 (63)	340 (37)	576 (67)	​
YES	271 (37)	582 (63)	280 (33)	​
Diabetes, n (%)	​	​	​	<0.001
NO	194 (26)	614 (67)	617 (72)	​
YES	539 (74)	308 (33)	239 (28)	​
Liver disease, n (%)	​	​	​	<0.001
NO	636 (87)	866 (94)	792 (93)	​
YES	97 (13)	56 (6.1)	64 (7.5)	​
Renal disease, n (%)	​	​	​	<0.001
NO	119 (16)	723 (78)	627 (73)	​
YES	614 (84)	199 (22)	229 (27)	​
Malignant cancer, n (%)	​	​	​	0.100
NO	649 (89)	823 (89)	784 (92)	​
YES	84 (11)	99 (11)	72 (8.4)	​
Hypertension, n (%)	​	​	​	<0.001
NO	652 (89)	526 (57)	516 (60)	​
YES	81 (11)	396 (43)	340 (40)	​
Cardiac arrhythmias, n (%)	​	​	​	<0.001
NO	283 (39)	323 (35)	255 (30)	​
YES	450 (61)	599 (65)	601 (70)	​
Diuretics, n (%)	​	​	​	0.041
NO	358 (49)	411 (45)	431 (50)	​
YES	375 (51)	511 (55)	425 (50)	​
Beta-blockers, n (%)	​	​	​	0.018
NO	434 (59)	511 (55)	531 (62)	​
YES	299 (41)	411 (45)	325 (38)	​
ACEI/ARB, n (%)	​	​	​	<0.001
NO	665 (91)	771 (84)	785 (92)	​
YES	68 (9.3)	151 (16)	71 (8.3)	​
Status 365, n (%)	281 (38)	290 (31)	197 (23)	<0.001

Data are presented as n (%) for categorical variables and as median (interquartile range) for continuous variables. Group comparisons were performed using Pearson’s chi-squared test for categorical variables and Kruskal–Wallis rank sum test for continuous variables. A two-sided *P* value <0.05 was considered statistically significant.

Abbreviations: BUN, blood urea nitrogen; EF, ejection fraction; HR, heart rate; MBP, mean blood pressure; MCHC, mean corpuscular hemoglobin concentration; MCV, mean corpuscular volume; PT, prothrombin time; PTT, partial thromboplastin time; RDW, red cell distribution width; RR, respiratory rate; SBP, systolic blood pressure; SpO_2_, peripheral oxygen saturation; WBC, white blood cell count.

Comorbidities were defined using ICD, codes. Status 365 denotes all-cause mortality by day 365 after ICU, admission; under the 24-h landmark design, entry into the risk set occurred at 24 h (excluding patients who died within the first 24 h), and the maximum post-landmark follow-up was 364 days.

For descriptive interpretation of the primary three-cluster solution, Cluster 1 (n = 733), designated as the “Cardiorenal-Metabolic Phenotype,” had the highest observed 365-day mortality (38%). Patients in this cluster were characterized by impaired renal and metabolic profiles, including higher creatinine (median, 2.2 mg/dL), higher glucose (154 mg/dL), and lower hematocrit (29.4%). Diabetes and renal disease were present in 74% and 84% of patients, respectively, indicating a prominent cardiorenal-metabolic burden. Cluster 2 (n = 922), designated as the “Hypertensive and Pulmonary Phenotype,” was characterized primarily by chronic pulmonary disease (63%) and hypertension (43%) and had an intermediate observed 365-day mortality risk. This group showed a distinct constellation of chronic cardiopulmonary disease. Cluster 3 (n = 856), designated as the “Low-Risk, Low Blood Pressure and Arrhythmia Phenotype,” had the lowest observed 365-day mortality (23%), the highest prevalence of arrhythmias (70%), and the lowest systolic (115 mmHg) and mean blood pressures (74 mmHg). The term “Low-Risk” refers specifically to the observed 365-day mortality proportion rather than overall acute clinical severity.

In the two-cluster sensitivity analysis, Cluster one included 1,040 patients (41.4%) and represented a broader high-burden group characterized by higher prevalences of diabetes, renal disease, and chronic pulmonary disease together with worse renal and metabolic markers. Cluster two included 1,471 patients (58.6%) and comprised an older group with lower renal–metabolic burden and higher prevalences of hypertension and cardiac arrhythmias. Observed 365-day mortality was 36.0% in Cluster 1 and 26.8% in Cluster 2 (*P* < 0.001). Compared with the primary three-cluster model, the two-cluster solution maintained the baseline higher-versus lower-mortality dichotomy, while merging the cardiorenal-metabolic and hypertensive-pulmonary profiles into a single group.

### 3.3 Survival outcomes across phenotypes

Kaplan-Meier analysis showed that the estimated survival probabilities differed significantly across the three phenotypic clusters (*P* < 0.0001). As shown in Figure 3, Cluster one had the lowest survival probability, Cluster two had an intermediate survival probability, and Cluster three had the highest survival probability. The observed 365-day mortality proportions were 38.3% (281/733) in Cluster 1, 31.5% (290/922) in Cluster 2, and 23.0% (197/856) in Cluster 3.

**FIGURE 3:**
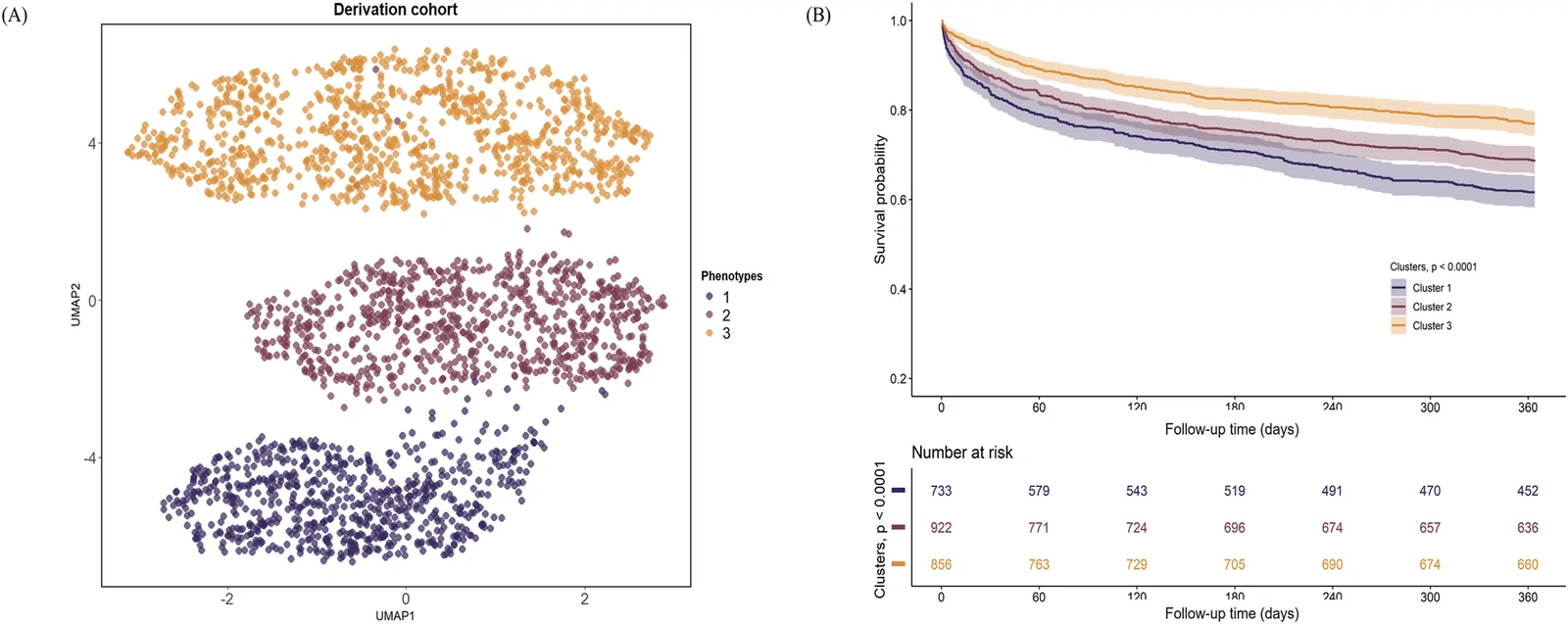
Phenotypic clustering and associated survival outcomes in the derivation cohort. **(A)** Uniform Manifold Approximation and Projection (UMAP) visualization of three assigned phenotypic clusters in the derivation cohort. **(B)** 365-day Kaplan–Meier survival curves for the three clusters, indicating a statistically significant difference in survival probability (log-rank test, *P* < 0.0001).

In the two-cluster sensitivity analysis, the observed 365-day mortality proportions were 36.0% (374/1,040) in Cluster 1 and 26.8% (394/1,471) in Cluster 2 (*P* < 0.001). The corresponding Kaplan–Meier curves are presented in Supplementary Figure S13A.

### 3.4 Exploratory cluster-specific medication associations

In the overall derivation cohort, diuretic exposure was not associated with 365-day mortality in the unadjusted, sIPTW, or OW analyses (Figures 4A–C). The unadjusted association for ACEI/ARB was attenuated after weighting but retained a raw *P* value below 0.05 under both sIPTW and OW; neither weighted estimate remained statistically significant after FDR correction. The unadjusted association for beta-blocker exposure was attenuated after weighting, with raw *P* values above 0.05 in both weighted analyses (Figures 4D–I).

**FIGURE 4:**
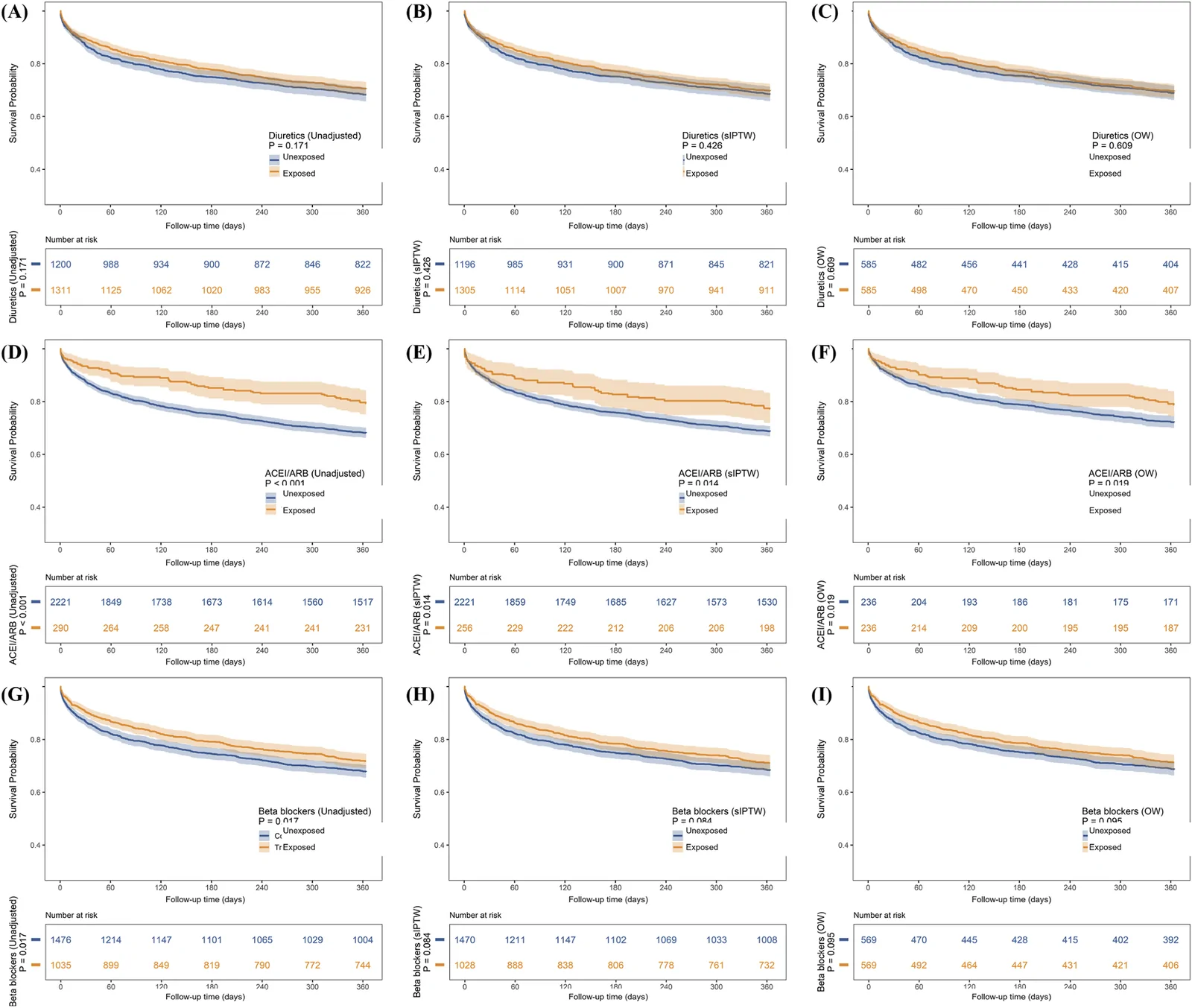
Associations between early medication exposure and 365-day all-cause mortality in the derivation cohort. KM curves illustrating the associations between early medication exposure and 365-day all-cause mortality under three analytical approaches: unadjusted **(A, D, G)**, sIPTW-adjusted **(B, E, H)**, and OW-adjusted **(C, F, I)**. *P* values in the unadjusted panels were calculated using the log-rank test, whereas *P* values in the weighted panels were derived from Wald tests of the corresponding weighted Cox proportional hazards models with robust variance estimators. ACEI, angiotensin-converting enzyme inhibitor; ARB, angiotensin receptor blocker; sIPTW, stabilized inverse probability of treatment weighting; OW, overlap weighting.

Cluster-specific estimates are presented in Figure 5. Unadjusted analyses were included as descriptive comparisons, OW was used as the primary weighted analysis, and sIPTW was used as a sensitivity analysis. In the OW analysis, the ACEI/ARB estimate in Cluster one had a raw *P* value below 0.05 (HR = 0.46, 95% CI, 0.26–0.82; *P* = 0.008), but did not remain statistically significant after FDR correction (FDR-adjusted *P* = 0.098). None of the other cluster-specific OW estimates for diuretics, ACEI/ARB, or beta-blockers had a raw *P* value below 0.05.

**FIGURE 5:**
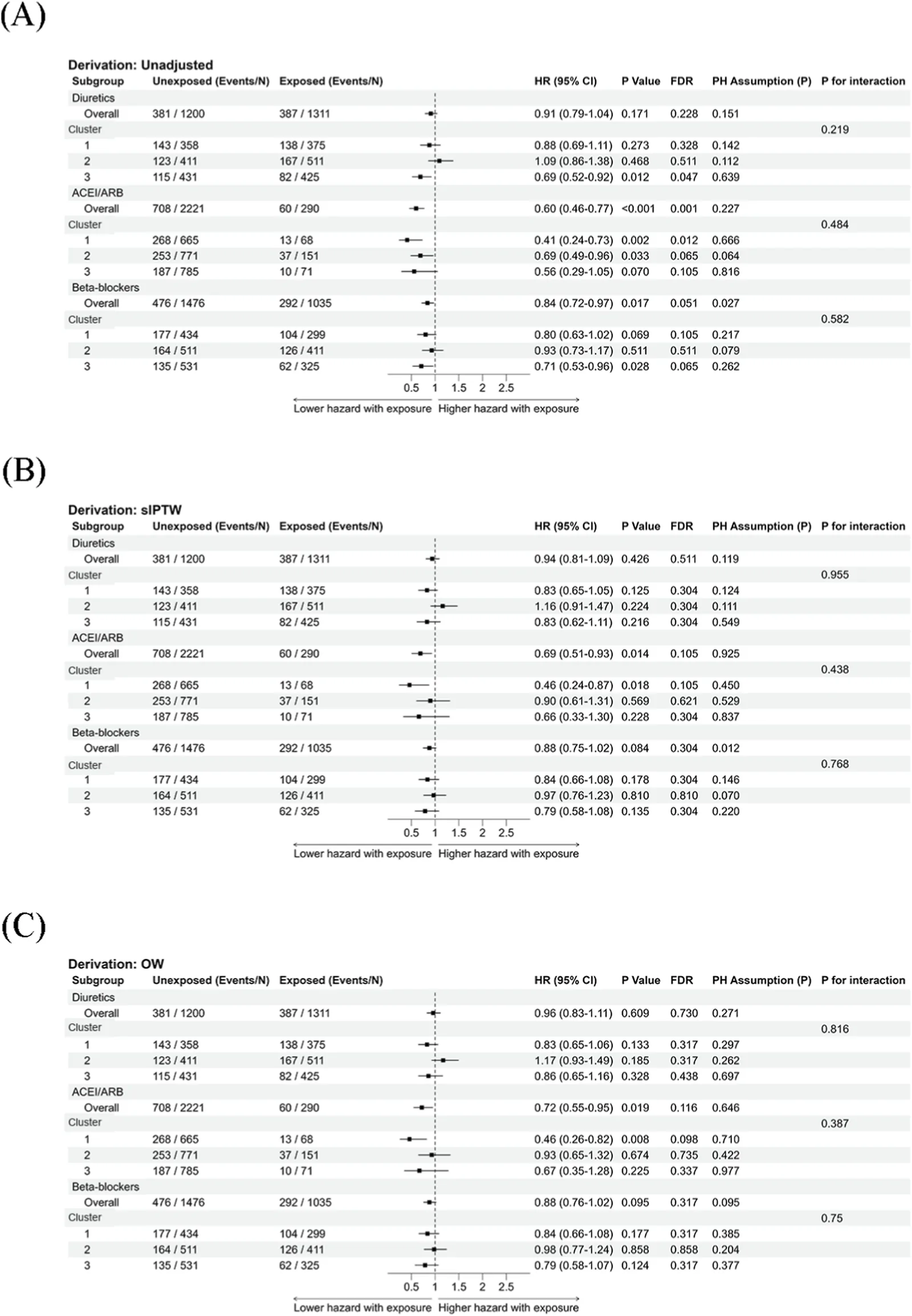
Cluster-specific associations between medication exposure and 365-day mortality in the derivation cohort. Forest plots show hazard ratios (HRs) and 95% confidence intervals (CIs) for medication exposure and 365-day mortality from **(A)** unadjusted, **(B)** stabilized inverse probability of treatment weighting (sIPTW), and **(C)** overlap weighting (OW) analyses. All medication–phenotype analyses were exploratory. Unadjusted estimates are presented descriptively; within the exploratory weighted analyses, OW was the primary approach and sIPTW was the sensitivity analysis. Results are shown overall and within each of the three phenotypic clusters for diuretics, ACEI/ARB, and beta-blockers. The tabulated columns report event/sample counts for the unexposed and exposed groups, HRs with 95% CIs, raw *P* values, Benjamini–Hochberg FDR-adjusted *P* values, proportional-hazards test *P* values from the corresponding overall or cluster-specific medication models, and medication-by-cluster interaction *P* values. Full interaction-model term-specific and global proportional-hazards diagnostics are reported in Supplementary Table S9.

In the sIPTW analysis, the ACEI/ARB estimate in Cluster one also had a raw *P* value below 0.05 (HR = 0.46, 95% CI, 0.24–0.87; *P* = 0.018), but its FDR-adjusted *P* value was 0.105. None of the other cluster-specific sIPTW estimates had a raw *P* value below 0.05. Across both weighted analyses, no cluster-specific estimate had an FDR-adjusted *P* value below 0.05. Medication-by-cluster interaction tests were nonsignificant for all three medication classes under both OW and sIPTW (all interaction *P* ≥ 0.387 under OW and ≥0.438 under sIPTW).

Propensity-score overlap, event counts, effective sample sizes, and covariate-balance diagnostics are summarized in Supplementary Table S4 and Supplementary Figure S16. All OW comparisons met the prespecified ASMD threshold, whereas residual covariate imbalance was present in selected sIPTW analyses of ACEI/ARB exposure. Complete term-specific and global proportional-hazards diagnostics are reported in Supplementary Table S9. In the primary OW models, no test rejected the proportional-hazards assumption (all *P* ≥ 0.095). In the sIPTW beta-blocker model, the medication main-effect term, conditional on the reference cluster, yielded *P* = 0.013, whereas the cluster main effect, medication-by-cluster interaction, and global tests yielded *P* = 0.192, *P* = 0.057, and *P* = 0.081, respectively. This term-specific non-proportionality signal occurred only in the sensitivity model; because the corresponding primary OW model and the sIPTW interaction and global tests did not reject proportional hazards, the constant sIPTW beta-blocker coefficient was not used as confirmatory evidence and should be interpreted cautiously.

In the two-cluster sensitivity analysis, none of the medication-by-cluster interaction tests was statistically significant, and no weighted cluster-specific estimate remained statistically significant after FDR correction (Supplementary Figure S14).

### 3.5 Internal validation of phenotypes and medication–outcome associations

In the MIMIC-III internal validation cohort (n = 1,524), *de novo* K-prototypes clustering assigned 425, 530, and 569 patients to Clusters 1, 2, and 3, respectively (Supplementary Figure S3A). The corresponding numbers of deaths within 365 days were 149 (35.1%), 162 (30.6%), and 161 (28.3%). Kaplan–Meier curves showed the lowest estimated survival in Cluster one and the highest estimated survival in Cluster 3, although the overall between-cluster difference did not reach statistical significance (log-rank *P* = 0.072; Supplementary Figure S3B). Cluster-level clinical characteristics are presented in Supplementary Figure S5 and Supplementary Table S2. Based on these characteristics, the validation-cohort clusters were aligned with the Cardiorenal-Metabolic Phenotype, Hypertensive and Pulmonary Phenotype, and Low-Risk, Low Blood Pressure and Arrhythmia Phenotype defined in the derivation cohort.

In the overall unadjusted medication analyses, raw *P* values were below 0.05 for diuretics, ACEI/ARB, and beta-blockers; after FDR correction, only the overall ACEI/ARB estimate had an adjusted *P* value below 0.05. In the weighted analyses, the overall ACEI/ARB estimate had a raw *P* value below 0.05 under sIPTW (HR = 0.64, 95% CI, 0.43–0.95; *P* = 0.027; FDR-adjusted *P* = 0.318), but not under OW (HR = 0.73, 95% CI, 0.52–1.04; *P* = 0.081; FDR-adjusted *P* = 0.729). The overall weighted estimates for diuretics and beta-blockers had raw *P* values above 0.05 under both weighting approaches (Supplementary Figure S4).

In the primary cluster-specific OW analysis, none of the estimates for diuretics, ACEI/ARB, or beta-blockers had a raw *P* value below 0.05. For diuretic exposure in Cluster 3, the HR was 0.83 (95% CI, 0.59–1.15; *P* = 0.258; FDR-adjusted *P* = 0.729). The ACEI/ARB estimate in Cluster one was 0.76 (95% CI, 0.40–1.46; *P* = 0.413; FDR-adjusted *P* = 0.729). No cluster-specific beta-blocker estimate had a raw *P* value below 0.05. The OW medication-by-cluster interaction *P* values were 0.192 for diuretics, 0.567 for ACEI/ARB, and 0.565 for beta-blockers.

Similarly, none of the cluster-specific estimates had a raw *P* value below 0.05 in the sIPTW analysis. The HR for diuretic exposure in Cluster three was 0.81 (95% CI, 0.58–1.13; *P* = 0.209; FDR-adjusted *P* = 0.627), and the ACEI/ARB estimate in Cluster one was 0.52 (95% CI, 0.23–1.16; *P* = 0.108; FDR-adjusted *P* = 0.433). The sIPTW medication-by-cluster interaction *P* values were 0.192, 0.916, and 0.594 for diuretics, ACEI/ARB, and beta-blockers, respectively. Across both weighted analyses, no overall or cluster-specific estimate had an FDR-adjusted *P* value below 0.05 (Supplementary Figure S6).

All OW comparisons in the internal validation cohort met the prespecified ASMD threshold. Under sIPTW, residual covariate imbalance was present in the overall, Cluster 1, and Cluster 3 ACEI/ARB analyses, whereas the diuretic and beta-blocker analyses met the balance threshold. No term-specific or global proportional-hazards test had a *P* value below 0.05 in the weighted models (minimum *P* = 0.190; Supplementary Table S9). The complete event-count, overlap, effective-sample-size, and balance diagnostics are reported in Supplementary Table S4 and Supplementary Figure S16.

In the two-cluster sensitivity analysis, the internal validation cohort was divided into groups of 638 and 886 patients, with a difference in survival between the groups (log-rank *P* < 0.0001; Supplementary Figure S13B). In contrast, the K = 3 internal-validation comparison yielded log-rank *P* = 0.072, indicating that prognostic separation was sensitive to clustering resolution. The medication-related conclusion was unchanged: none of the weighted cluster-specific estimates had a raw *P* value below 0.05 or remained statistically significant after FDR correction, and none of the medication-by-cluster interaction tests was statistically significant under OW or sIPTW (Supplementary Figure S15).

### 3.6 Phenotype classification model development and internal validation

To construct a research tool for assigning the three predefined HFpEF labels, we developed a phenotype-classification model based on TabPFN. The classifier was designed to reproduce the machine-derived phenotype labels rather than to predict mortality or treatment response. MIMIC-IV served as the derivation cohort, and MIMIC-III was used for internal validation against labels obtained by *de novo* reclustering. The 37-predictor model achieved the highest absolute performance in the RFE comparison; among the reduced-input configurations, the 14-predictor model performed best and was retained to limit manual data-entry burden. The selected variables were anion gap, creatinine, BUN, chloride, respiratory rate, glucose, renal disease, SpO_2_, heart rate, platelet count, mean blood pressure, calcium, hematocrit, and systolic blood pressure (Supplementary Figure S7).

For internal validation, the three phenotypes were first generated independently in MIMIC-III by applying the same K-prototypes workflow *de novo*. Before classifier evaluation, the one-to-one correspondence with the MIMIC-IV phenotype identities was determined by Hungarian assignment that minimized the prespecified cluster-profile distance described in the Methods, independently of classifier predictions, survival outcomes, and medication exposures. The resulting correspondence was Cluster one to Cluster 1, Cluster two to Cluster 2, and Cluster three to Cluster 3. The TabPFN model trained exclusively in MIMIC-IV was not refitted in MIMIC-III. Instead, the 14 clinical variables from each MIMIC-III patient were entered into the fixed classifier, and the resulting phenotype-specific prediction probabilities were compared with the independently reclustered and Hungarian-aligned MIMIC-III reference labels.

In the internal validation cohort, the one-versus-rest AUC was 0.969 (95% CI, 0.961–0.976) for Cluster 1, 0.961 (95% CI, 0.952–0.969) for Cluster 2, and 0.951 (95% CI, 0.941–0.961) for Cluster 3 (Figure 6). The corresponding derivation-cohort AUCs were 0.976 (95% CI, 0.971–0.981), 0.966 (95% CI, 0.960–0.972), and 0.973 (95% CI, 0.968–0.978), respectively. After the prespecified Hungarian alignment of the internal-validation reference clusters, the calibration result was 10-bin multiclass ECE = 0.028 (Supplementary Figure S20D). Additional benchmarking, confusion-matrix, precision–recall, and phenotype-specific calibration results are presented in Supplementary Figures S18–S19.

**FIGURE 6:**
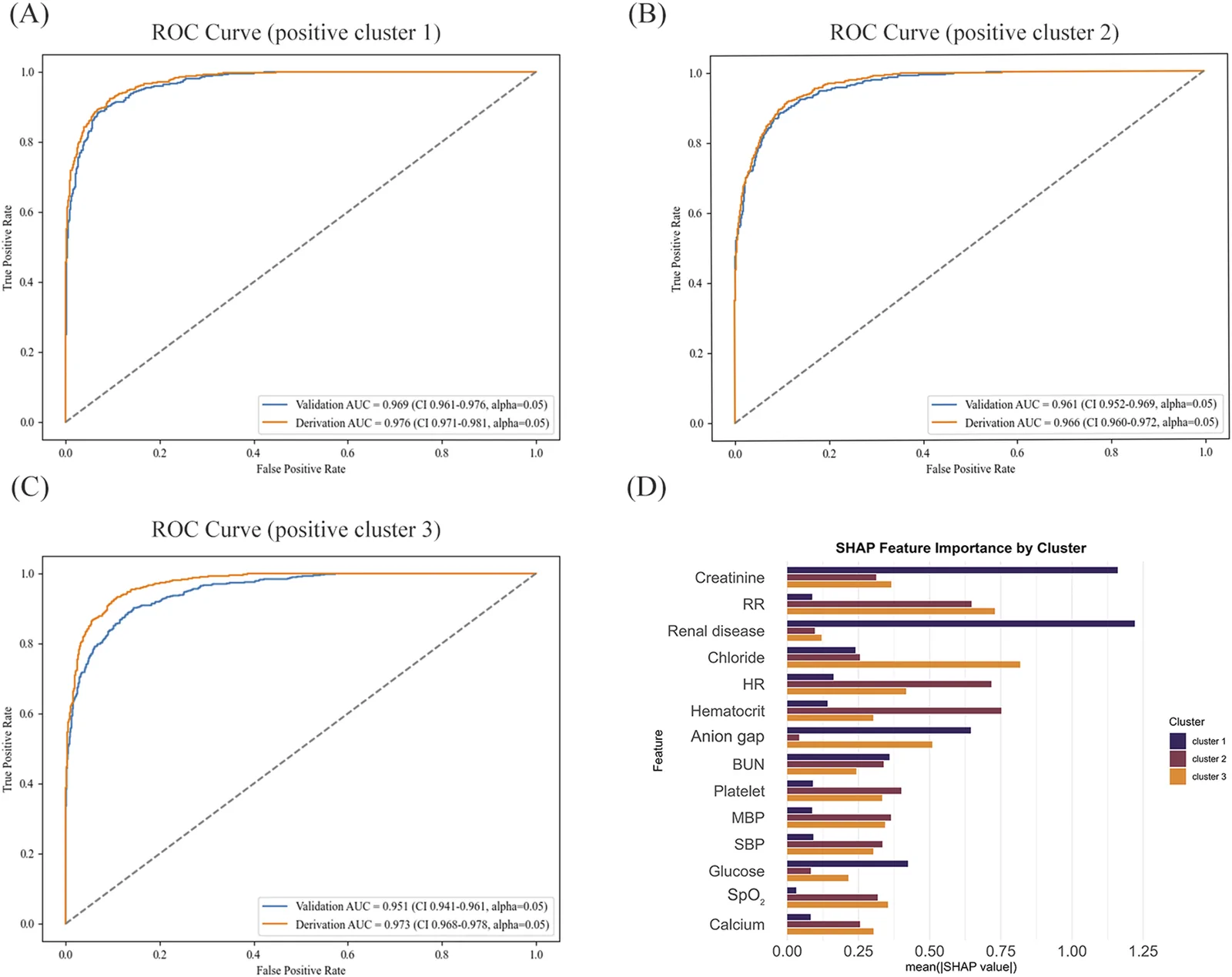
Phenotype-label discrimination and feature attribution of the HFpEF phenotype classifier. **(A–C)** One-vs-rest receiver operating characteristic curves for Clusters 1, 2, and 3 in the derivation and internal validation cohorts. AUC values are annotated for both cohorts. **(D)** Mean absolute SHAP values from an auxiliary LightGBM classifier trained using the same 14 features and phenotype labels summarize cluster-specific feature-attribution patterns. Abbreviations: BUN, blood urea nitrogen; HR, heart rate; MBP, mean blood pressure; RR, respiratory rate; SBP, systolic blood pressure; SpO_2_, peripheral oxygen saturation.

Single-feature ablation and representative case analyses were additionally performed in the internal validation cohort. Single-feature ablation showed that classifier performance was most sensitive to the removal of hematocrit, respiratory rate, glucose, and renal disease, which produced the largest reductions in macro-F1 score (Δmacro-F1 = 0.0320, 0.0253, 0.0240, and 0.0232, respectively; Supplementary Table S7). Predicted phenotype probabilities for representative correctly classified, misclassified, and low-confidence cases are reported in Supplementary Table S8.

SHAP analysis was performed using an auxiliary LightGBM classifier trained on the same 14 features and phenotype labels (Figure 6). Based on mean absolute SHAP values, the highest-ranked features included renal disease, creatinine, and anion gap for Cluster 1; hematocrit and HR for Cluster 2; and chloride and RR for Cluster 3. To facilitate phenotype assignment for research purposes, the TabPFN model has been deployed as an interactive, web-based phenotype classification interface, which is publicly accessible at: https://hfpefphenotype-2palxheyhzal63qswgto2n.streamlit.app (Supplementary Figure S8).

### 3.7 External application of the phenotype classifier and medication–outcome associations

The fixed MIMIC-IV-trained TabPFN classifier was applied without refitting to the external application cohort (n = 340) and assigned each patient to one of the three predefined phenotype labels. The classifier-assigned groups comprised 101, 116, and 123 patients in Clusters 1, 2, and 3, respectively. Their UMAP visualization and clinical characteristics are reported in Supplementary Figures S9, S11 and Supplementary Table S3. Independently generated reference phenotype labels were unavailable; therefore, external classification accuracy was not calculated. Among the 14 classifier variables, pre-imputation external-cohort missingness ranged from 0% to 22.35%, and several variables showed meaningful distribution differences from the derivation cohort (maximum ASMD, approximately 0.55; Supplementary Table S6). The distribution of maximum predicted probability had a median of 0.791 (IQR, 0.624–0.941); 77 of 340 assignments (22.6%) had a maximum probability below the descriptive 0.60 threshold. The median normalized entropy was 0.509 (IQR, 0.228–0.700; Supplementary Figure S20A-C).

The observed 365-day mortality rates were 43%, 38%, and 33% in Clusters 1, 2, and 3, respectively, with no statistically significant overall difference (*P* = 0.300; Supplementary Table S3). The corresponding Kaplan–Meier curves did not differ significantly across the classifier-assigned groups (log-rank *P* = 0.37; Supplementary Figure S9B).

For descriptive context in the external application cohort, the unadjusted overall diuretic estimate was HR = 0.51 (95% CI, 0.34–0.78; *P* = 0.002; FDR-adjusted *P* = 0.025). The descriptive OW and sIPTW estimates were both 0.55 (OW: 95% CI, 0.35–0.85; *P* = 0.007; FDR-adjusted *P* = 0.082; sIPTW: 95% CI, 0.35–0.86; *P* = 0.009; FDR-adjusted *P* = 0.105). The overall and cluster-specific ACEI/ARB and beta-blocker estimates did not have raw *P* values below 0.05 in either weighted summary (Supplementary Figures S10, S12). Because adequate covariate balance was not achieved within classifier-assigned groups, none of these external estimates was used for adjusted, causal, or confirmatory inference.

The external cluster-specific medication estimates were also retained only as descriptive summaries. None had a raw or FDR-adjusted *P* value below 0.05. For diuretic exposure in Cluster 3, the HR was 0.54 in the OW summary (95% CI, 0.25–1.18; *P* = 0.126; FDR-adjusted *P* = 0.372) and 0.59 in the sIPTW summary (95% CI, 0.27–1.29; *P* = 0.186; FDR-adjusted *P* = 0.339). The displayed medication-by-cluster interaction *P* values and proportional-hazards *P* values are provided for descriptive completeness and were not interpreted as formal evidence of homogeneity, treatment association, or model validity in the external cohort.

Across medication-specific overall and cluster strata, propensity-score overlap ranged from 80.2% to 97.3%. Because the external cohort-level propensity-score models did not include cluster interactions, all nine cluster-specific comparisons retained residual imbalance after weighting: maximum post-OW ASMDs ranged from 0.220 to 0.489, with 4–11 covariates exceeding 0.10. The sparsest comparison included 16 ACEI/ARB-exposed patients and four exposed events in Cluster 3. Consequently, the external medication results were treated as descriptive and were excluded from confirmatory interpretation. Complete event-count, overlap, effective-sample-size, summary balance, and covariate-specific ASMD diagnostics are reported in Supplementary Table S4 and Supplementary Figure S17.

## 4 Discussion

In this study, clustering of routinely available clinical variables collected during the first 24 h of ICU admission identified three clinically interpretable but substantially overlapping groups among patients with HFpEF. These groups differed principally in their cardiorenal and metabolic burden, hypertensive and pulmonary characteristics, and the combination of lower blood pressure and arrhythmias. Although a graded difference in 365-day mortality was observed in the derivation cohort, the between-cluster separation was attenuated and did not reach statistical significance in the internal validation or external application cohort. In the exploratory medication analyses, no weighted cluster-specific association remained statistically significant after correction for multiple comparisons, and no formal medication-by-cluster interaction was statistically significant in the derivation or internal validation cohort. External medication estimates were descriptive because within-group balance was inadequate. The K = 2 solution yielded the same medication-related conclusion, but prognostic separation differed between K = 2 and K = 3. In parallel, the TabPFN classifier showed high discrimination for the clustering-derived labels in internal validation and was subsequently applied to an external application cohort. Taken together, these findings support exploratory HFpEF phenotype characterization and reproducible assignment of machine-derived labels, but they do not establish discrete biological subtypes, stable prognostic categories, or phenotype-specific treatment effects.

The number of clusters was selected by considering quantitative clustering indices together with stability and clinical interpretability rather than by relying on a single statistical criterion. The two-cluster solution had the highest average silhouette width, whereas the average silhouette width for the three-cluster solution was low at 0.06, and the elbow analysis did not identify an unequivocal optimum at three clusters. K = 3 was therefore not considered statistically superior to K = 2. It was retained as the main exploratory representation because it separated three clinically recognizable combinations of characteristics without generating very small groups, while K = 2 was treated as an equally plausible lower-resolution sensitivity solution. The K = 2 analysis supported the same narrow medication-related conclusion—no FDR-significant weighted cluster-specific association and no significant medication-by-cluster interaction—but its stronger internal-validation survival separation showed that prognostic stratification depended on the selected resolution. The low silhouette widths indicate substantial overlap and are consistent with continuously distributed clinical characteristics rather than sharply separated categories. Accordingly, K = 3 should be interpreted as a clinically oriented summary of heterogeneity, not as evidence of three mutually exclusive biological forms of HFpEF or superiority over alternative cluster structures.

The clinical characteristics of the three groups showed descriptive similarities to phenotypes reported in previous HFpEF phenomapping studies, although differences in study populations, input variables, and clustering methods preclude direct equivalence. Cluster 1, designated the Cardiorenal-Metabolic Phenotype, was characterized by a greater burden of renal dysfunction, diabetes, and other metabolic comorbidities and shared selected characteristics with previously described multimorbid, obese/diabetic, and renal-impairment phenotypes (Shah et al., 2015; Kao et al., 2015). Cluster 2, the Hypertensive and Pulmonary Phenotype, combined hypertension and pulmonary comorbidity with respiratory and hemodynamic features that partially resembled previously reported load-associated or mechanical-load groups (Shah et al., 2015; Hedman et al., 2020). Cluster 3, designated the Low-Risk, Low Blood Pressure and Arrhythmia Phenotype on the basis of its derivation-cohort profile, included older patients with lower blood pressure, a greater prevalence of arrhythmias, and comparatively lower cardiorenal and metabolic burden, with selected similarities to previously reported older or atrial-fibrillation–predominant phenotypes. These parallels provide clinical context for the observed group profiles but do not demonstrate shared pathophysiological mechanisms. Moreover, the observed mortality gradient in the derivation cohort was not reproduced as a statistically significant between-cluster difference in the internal validation or external cohorts. Differences in sample size, patient acuity, comorbidity distributions, healthcare settings, and LVEF distribution, including the higher and more continuously distributed LVEF in the external cohort, may have contributed to variation in cluster expression and prognostic separation. The use of routine first-24-h ICU variables, rather than detailed echocardiographic, imaging, congestion, or biomarker measurements, may also have limited the resolution of the derived groups. Thus, selected clinical characteristics appeared transportable across cohorts, whereas cluster prevalence and prognostic separation remained dependent on the population and setting in which the phenotyping framework was applied. The broader rationale for phenotype-oriented interpretation is also consistent with multiorgan and diastolic-function frameworks of HFpEF (Shah et al., 2016; Obokata et al., 2020). The pulmonary component of Cluster two is clinically plausible given evidence that COPD is common in HFpEF, complicates diagnosis and treatment, and is associated with adverse outcomes (Mooney et al., 2021; Xu et al., 2025; Canepa et al., 2019).

The exploratory medication analyses did not provide statistical evidence that associations of diuretics, ACEI/ARB, or beta-blockers with 365-day mortality differed among the identified phenotypes. Within the derivation and internal validation cohorts, unadjusted estimates were descriptive, OW was the primary weighted approach, and sIPTW was a sensitivity analysis. Selected estimates had raw *P* values below 0.05, but none of the cluster-specific estimates remained statistically significant after FDR correction, and no medication-by-cluster interaction was significant. The K = 2 analysis supported this same limited medication-related conclusion. In the external application cohort, all medication estimates were reported only descriptively because adequate within-group covariate balance was not achieved; therefore, their *P* values were not interpreted as confirmatory evidence. The term-specific PH signal for the beta-blocker coefficient in the derivation-cohort sIPTW sensitivity model further precluded interpreting that coefficient as a constant effect over follow-up; the corresponding primary OW model did not reject PH, and the sIPTW coefficient was not used as confirmatory evidence. Failure to detect a statistically significant interaction does not demonstrate identical medication associations across phenotypes, particularly with limited subgroup sample sizes and events. These analyses do not support a claim of phenotype-specific treatment benefit or provide a basis for medication selection by cluster membership. These null subgroup findings should also be interpreted against the heterogeneous clinical indications for these drug classes. Diuretics are used primarily to relieve congestion, and changes in renal function during decongestion do not necessarily reflect tubular injury (Felker et al., 2020; Mullens et al., 2019; Ahmad et al., 2018). ACEI/ARB use is influenced by renal function and hyperkalemia risk (Georgianos and Agarwal, 2018). Evidence for beta-blockers in HFpEF remains inconsistent and is strongly conditioned by rhythm, ejection fraction, and comorbid indications (Cleland et al., 2018; Silverman et al., 2019; Arnold et al., 2023); concomitant COPD further complicates prescribing and interpretation (Dransfield et al., 2019; Lipworth et al., 2016).

The phenotype classifier addressed a different objective from the medication analyses: it was developed to reproduce clustering-derived phenotype labels rather than to predict mortality, treatment response, or clinical benefit. The 14-variable configuration was the best-performing reduced-input option, although the 37-variable model had the highest absolute cross-validated performance; the smaller configuration was retained as an explicit performance–input-burden compromise for the research interface. Using these 14 routinely available variables, the fixed TabPFN classifier achieved one-versus-rest AUCs ranging from 0.951 to 0.969 in internal validation. After prespecified Hungarian alignment of the independently reclustered MIMIC-III groups on cluster-profile distances, the 10-bin multiclass expected calibration error was 0.028. The alignment was defined without classifier predictions, survival outcomes, or medication exposures. Feature-ablation and surrogate-model SHAP analyses improved transparency but did not identify causal phenotype determinants or directly explain TabPFN predictions. In the external application cohort, independently generated reference labels were unavailable; therefore, the analysis focused on classifier application, feature-distribution shift, missingness, prediction uncertainty, and descriptive transportability rather than independent validation of the clustering structure or external classification accuracy. The web interface should be regarded as a research tool for standardized phenotype-label assignment, not as a clinical decision-support system or treatment-selection method.

Recent cardiovascular and cross-domain biomedical machine-learning literature has likewise highlighted predictive evaluation, feature integration, interpretability, and rigorous validation as recurring methodological priorities (Lekadir et al., 2025; Ghassemi et al., 2021; Banerjee and Pacal, 2025; Pacal and Banerjee, 2026; Banerjee, 2026). In the present study, classifier discrimination, calibration, uncertainty, and SHAP outputs were therefore interpreted as model-assessment results rather than evidence of clinical readiness.

Several limitations should be considered when interpreting these findings. First, the retrospective observational design precludes causal interpretation of the exploratory medication–mortality associations. Although OW and sIPTW reduced imbalance in measured baseline variables in the derivation and internal validation cohorts, residual confounding, confounding by indication, treatment contraindications, and unmeasured severity differences may remain. Exposure during the first 24 h did not capture indication, route, dose, duration, discontinuation, discharge prescriptions, adherence, or subsequent treatment changes. Second, the cluster structure may be sensitive to variable selection, missing-data handling, cohort composition, and algorithmic choices. The low silhouette widths indicate substantial overlap; the stronger internal-validation survival separation under K = 2 than K = 3 demonstrates that prognostic stratification was resolution-dependent. The cluster labels should not be treated as biological ground truth. Multiple medication–cluster comparisons, limited subgroup events, and wide confidence intervals reduced precision. In the external application cohort, fully interacted propensity-score and simplified doubly robust models could not be estimated reliably because of sparse medication–cluster strata, and all nine cluster-specific weighted comparisons retained residual imbalance. External medication estimates were therefore descriptive and were not used for causal, adjusted, or confirmatory inference. Third, the classifier reproduced data-derived labels from related clinical variables; high internal discrimination does not establish biological validity, prognostic utility, or treatment-response prediction. The external cohort lacked independent reference labels, and differences in distributions, missingness, acuity, LVEF, comorbidities, and healthcare settings may affect assignments and confidence. SHAP values came from a LightGBM surrogate and do not directly explain TabPFN. Finally, the ICU-based populations limit generalizability. Prospective multicenter studies should independently derive and compare phenotype structures, incorporate richer imaging, congestion, biomarker, and longitudinal-treatment data, evaluate phenotype stability over time, and test treatment hypotheses using prespecified phenotype-stratified designs before clinical use is considered.

## 5 Conclusion

This study provides an exploratory framework for characterizing clinical heterogeneity among ICU patients with HFpEF using routinely collected data and for reproducibly assigning machine-derived phenotype labels. The identified phenotypes were overlapping and their prognostic expression varied across cohorts, while the analyses did not establish phenotype-specific medication effects. The classifier and web interface should therefore be regarded as research tools, and prospective multicenter validation is required before the framework is used for prognostic stratification or treatment guidance.

## Data Availability

The datasets presented in this article are not readily available because the clinical data from the Second Hospital of Hebei Medical University are subject to institutional privacy regulations and ethical restrictions. Therefore, these data are not publicly available. However, they can be made available from the corresponding authors on reasonable request and strictly with the prior permission of the Research Ethics Committee of the Second Hospital of Hebei Medical University. The public datasets (MIMIC-III and MIMIC-IV) analyzed in this study are available through PhysioNet. Requests to access the datasets should be directed to BX, xiaobing@hebmu.edu.cn.

## Glossary

ACEI: Angiotensin-converting enzyme inhibitor
AG: Anion gap
ARB: Angiotensin receptor blocker
ARI: Adjusted Rand index
ASMD: Absolute standardized mean difference
AUC: Area under the receiver operating characteristic curve
BUN: Blood urea nitrogen
CI: Confidence interval
EF: Ejection fraction
ECE: Expected calibration error
FDR: False discovery rate
HFpEF: Heart failure with preserved ejection fraction
HR: Hazard ratio
ICD: International Classification of Diseases
ICU: Intensive care unit
IQR: Interquartile range
KM: Kaplan–Meier
KNN: K-nearest neighbors
LVEF: Left ventricular ejection fraction
MBP: Mean blood pressure
MCHC: Mean corpuscular hemoglobin concentration
MCV: Mean corpuscular volume
MIMIC: Medical Information Mart for Intensive Care
OW: Overlap weighting
PH: Proportional hazards
PT: Prothrombin time
PTT: Partial thromboplastin time
RDW: Red cell distribution width
RFE: Recursive feature elimination
ROC: Receiver operating characteristic
RR: Respiratory rate
SBP: Systolic blood pressure
SHAP: SHapley Additive exPlanations
sIPTW: Stabilized inverse probability of treatment weighting
**SpO**_**2**_: Peripheral oxygen saturation
TabPFN: Tabular Prior-Data Fitted Network
UMAP: Uniform Manifold Approximation and Projection
WBC: White blood cell count

## References

[B1] Ahmad T. Jackson K. Rao V. S. Tang W. H. W. Brisco-Bacik M. A. Chen H. H. (2018). Worsening renal function in patients with acute heart failure undergoing aggressive diuresis is not associated with tubular injury. Circulation 137 (19), 2016–2028. 10.1161/CIRCULATIONAHA.117.030112 29352071 PMC6066176

[B2] Arnold S. V. Silverman D. N. Gosch K. Nassif M. E. Infeld M. Litwin S. (2023). Beta-Blocker use and heart failure outcomes in mildly reduced and preserved ejection fraction. JACC Heart Fail 11 (8 Pt 1), 893–900. 10.1016/j.jchf.2023.03.017 37140513

[B3] Banerjee T. (2026). Comparing bipartite convoluted and attention-driven methods for skin cancer detection: a review of explainable AI and transfer learning strategies. Arch. Comput. Methods Eng. 33, 2579–2603. 10.1007/s11831-025-10379-5

[B4] Banerjee T. Pacal I. (2025). A systematic review of machine learning in heart disease prediction. Turk J. Biol. 49 (5), 600–634. 10.55730/1300-0152.2766 41246228 PMC12614364

[B5] Borlaug B. A. Sharma K. Shah S. J. Ho J. E. (2023). Heart failure with preserved ejection fraction: JACC scientific statement. J. Am. Coll. Cardiol. 81 (18), 1810–1834. 10.1016/j.jacc.2023.01.049 37137592

[B6] Campbell P. Rutten F. H. Lee M. M. Hawkins N. M. Petrie M. C. (2024). Heart failure with preserved ejection fraction: everything the clinician needs to know. Lancet 403 (10431), 1083–1092. 10.1016/s0140-6736(23)02756-3 38367642

[B7] Canepa M. Franssen F. M. E. Olschewski H. Lainscak M. Böhm M. Tavazzi L. (2019). Diagnostic and therapeutic gaps in patients with heart failure and chronic obstructive pulmonary disease. JACC Heart Fail 7 (10), 823–833. 10.1016/j.jchf.2019.05.009 31521680

[B8] Cleland J. G. F. Bunting K. V. Flather M. D. Altman D. G. Holmes J. Coats A. J. S. (2018). Beta-blockers for heart failure with reduced, mid-range, and preserved ejection fraction: an individual patient-level analysis of double-blind randomized trials. Eur. Heart J. 39 (1), 26–35. 10.1093/eurheartj/ehx564 29040525 PMC5837435

[B9] Collins G. S. Moons K. G. M. Dhiman P. Riley R. D. Beam A. L. Van Calster B. (2024). TRIPOD+AI statement: updated guidance for reporting clinical prediction models that use regression or machine learning methods. BMJ 385, e078378. 10.1136/bmj-2023-078378 38626948 PMC11019967

[B10] Desai R. J. Franklin J. M. (2019). Alternative approaches for confounding adjustment in observational studies using weighting based on the propensity score: a primer for practitioners. BMJ 367, l5657. 10.1136/bmj.l5657 31645336

[B11] Dransfield M. T. Voelker H. Bhatt S. P. Brenner K. Casaburi R. Come C. E. (2019). Metoprolol for the prevention of acute exacerbations of COPD. N. Engl. J. Med. 381 (24), 2304–2314. 10.1056/NEJMoa1908142 31633896 PMC7416529

[B12] Felker G. M. Ellison D. H. Mullens W. Cox Z. L. Testani J. M. (2020). Diuretic therapy for patients with heart failure: JACC state-of-the-art review. J. Am. Coll. Cardiol. 75 (10), 1178–1195. 10.1016/j.jacc.2019.12.059 32164892

[B13] Georgianos P. I. Agarwal R. (2018). Revisiting RAAS blockade in CKD with newer potassium-binding drugs. Kidney Int. 93 (2), 325–334. 10.1016/j.kint.2017.08.038 29276100 PMC5794635

[B14] Ghassemi M. Oakden-Rayner L. Beam A. L. (2021). The false hope of current approaches to explainable artificial intelligence in health care. Lancet Digit. Health 3 (11), e745–e750. 10.1016/S2589-7500(21)00208-9 34711379

[B15] Hedman Å. K. Hage C. Sharma A. Brosnan M. J. Buckbinder L. Gan L. M. (2020). Identification of novel pheno-groups in heart failure with preserved ejection fraction using machine learning. Heart 106 (5), 342–349. 10.1136/heartjnl-2019-315481 31911501

[B16] Heidenreich P. A. Bozkurt B. Aguilar D. Allen L. A. Byun J. J. Colvin M. M. (2022). AHA/ACC/HFSA Guideline for the management of heart failure: a report of the American college of Cardiology/American heart Association Joint Committee on clinical practice Guidelines. Circulation, 145(18): p. e895-e1032. 10.1161/CIR.0000000000001063 35363499

[B17] Hollmann N. Müller S. Purucker L. Krishnakumar A. Körfer M. Hoo S. B. (2025). Accurate predictions on small data with a tabular foundation model. Nature 637, 319–326. 10.1038/s41586-024-08328-6 39780007 PMC11711098

[B18] Johnson A. E. W. Pollard T. J. Shen L. Lehman L. W. H. Feng M. Ghassemi M. (2016). MIMIC-III, a freely accessible critical care database. Sci. Data 3, 160035. 10.1038/sdata.2016.35 27219127 PMC4878278

[B19] Johnson A. E. W. Bulgarelli L. Shen L. Gayles A. Shammout A. Horng S. (2023). MIMIC-IV, a freely accessible electronic health record dataset. Sci. Data 10 (1), 1. 10.1038/s41597-022-01899-x 36596836 PMC9810617

[B20] Kao D. P. Lewsey J. D. Anand I. S. Massie B. M. Zile M. R. Carson P. E. (2015). Characterization of subgroups of heart failure patients with preserved ejection fraction with possible implications for prognosis and treatment response. Eur. J. Heart Fail 17 (9), 925–935. 10.1002/ejhf.327 26250359 PMC4654630

[B21] Lekadir K. Frangi A. F. Porras A. R. Glocker B. Cintas C. Langlotz C. P. (2025). FUTURE-AI: international consensus guideline for trustworthy and deployable artificial intelligence in healthcare. BMJ 388, e081554. 10.1136/bmj-2024-081554 39909534 PMC11795397

[B22] Lipworth B. Wedzicha J. Devereux G. Vestbo J. Dransfield M. T. (2016). Beta-blockers in COPD: time for reappraisal. Eur. Respir. J. 48 (3), 880–888. 10.1183/13993003.01847-2015 27390282

[B23] Metra M. Tomasoni D. Adamo M. Amir O. Anker S. D. Bayes-Genis A. (2025). SGLT2 inhibitors for the prevention and treatment of heart failure: a scientific statement of the HFA and the HFAI. Esc. Heart Fail 12 (6), 3806–3825. 10.1002/ehf2.15408 40968746 PMC12719827

[B24] Mooney L. Hawkins N. M. Jhund P. S. Redfield M. M. Vaduganathan M. Desai A. S. (2021). Impact of chronic obstructive pulmonary disease in patients with heart failure with preserved ejection fraction: insights from PARAGON-HF. J. Am. Heart Assoc. 10 (23), e021494. 10.1161/JAHA.121.021494 34796742 PMC9075384

[B25] Mullens W. Damman K. Harjola V. P. Mebazaa A. Brunner-La Rocca H. P. Martens P. (2019). The use of diuretics in heart failure with congestion - a position statement from the Heart Failure Association of the European Society of Cardiology. Eur. J. Heart Fail 21 (2), 137–155. 10.1002/ejhf.1369 30600580

[B26] Obokata M. Reddy Y. N. V. Borlaug B. A. (2020). Diastolic dysfunction and heart failure with preserved ejection fraction: understanding mechanisms by using noninvasive methods. JACC Cardiovasc Imaging 13 (1 Pt 2), 245–257. 10.1016/j.jcmg.2018.12.034 31202759 PMC6899218

[B27] Pacal I. Banerjee T. (2026). Towards accurate and interpretable brain tumor diagnosis: t-fspannet with Tri-Attribute and pyramidal attention-based feature fusion. Biomed. Signal Process Control 113 (Pt A), 108852. 10.1016/j.bspc.2025.108852

[B28] Peikert A. Bart B. A. Vaduganathan M. Claggett B. L. Kulac I. J. Kosiborod M. N. (2024). Contemporary use and implications of beta-blockers in patients with HFmrEF or HFpEF: the DELIVER trial. JACC Heart Fail 12 (4), 631–644. 10.1016/j.jchf.2023.09.007 37767674

[B29] Redfield M. M. (2016). Heart failure with preserved ejection fraction. N. Engl. J. Med. 375 (19), 1868–1877. 10.1056/nejmcp1511175 27959663

[B30] Savarese G. Becher P. M. Lund L. H. Seferovic P. Rosano G. M. C. Coats A. J. S. (2023). Global burden of heart failure: a comprehensive and updated review of epidemiology. Cardiovasc Res. 118 (17), 3272–3287. 10.1093/cvr/cvac013 35150240

[B31] Segar M. W. Patel K. V. Ayers C. Basit M. Tang W. H. W. Willett D. (2020). Phenomapping of patients with heart failure with preserved ejection fraction using machine learning-based unsupervised cluster analysis. Eur. J. Heart Fail 22 (1), 148–158. 10.1002/ejhf.1621 31637815

[B32] Shah S. J. Katz D. H. Selvaraj S. Burke M. A. Yancy C. W. Gheorghiade M. (2015). Phenomapping for novel classification of heart failure with preserved ejection fraction. Circulation 131 (3), 269–279. 10.1161/circulationaha.114.010637 25398313 PMC4302027

[B33] Shah S. J. Kitzman D. W. Borlaug B. A. van Heerebeek L. Zile M. R. Kass D. A. (2016). Phenotype-Specific treatment of heart failure with preserved ejection fraction: a Multiorgan roadmap. Circulation 134 (1), 73–90. 10.1161/CIRCULATIONAHA.116.021884 27358439 PMC4930115

[B34] Silverman D. N. Plante T. B. Infeld M. Callas P. W. Juraschek S. P. Dougherty G. B. (2019). Association of β-Blocker use with heart failure hospitalizations and cardiovascular disease mortality among patients with heart failure with a preserved ejection fraction: a secondary analysis of the TOPCAT trial. JAMA Netw. Open 2 (12), e1916598. 10.1001/jamanetworkopen.2019.16598 31800067 PMC6902757

[B35] Solomon S. D. McMurray J. J. V. Vaduganathan M. Claggett B. L. Jhund P. S. Desai A. S. (2024). Finerenone in heart failure with mildly reduced or preserved ejection fraction. N. Engl. J. Med. 391 (16), 1475–1485. 10.1056/NEJMoa2407107 39225278

[B36] Vaduganathan M. Docherty K. F. Claggett B. L. Jhund P. S. de Boer R. A. Hernandez A. F. (2022). SGLT-2 inhibitors in patients with heart failure: a comprehensive meta-analysis of five randomised controlled trials. Lancet 400 (10354), 757–767. 10.1016/S0140-6736(22)01429-5 36041474

[B37] Vilaro J. R. (2023). Heart failure with preserved ejection fraction: important things to know about the stiff heart. Cardiovasc Innov. Appl. 8 (1), e954. 10.15212/CVIA.2023.0058

[B38] Wei J. Du J. (2023). Mechanisms of sodium-glucose cotransporter 2 inhibitors in heart failure. Cardiovasc Innov. Appl. 8 (1), e987. 10.15212/CVIA.2023.0028

[B39] Xu S. Gu Z. Zhu W. Feng S. (2025). Association of COPD with adverse outcomes in heart failure patients with preserved ejection fraction. Esc. Heart Fail 12 (2), 799–808. 10.1002/ehf2.14958 38993173 PMC11911644

[B40] Zafeiropoulos S. Farmakis I. T. Milioglou I. Doundoulakis I. Gorodeski E. Z. Konstantinides S. V. (2024). Pharmacological treatments in heart failure with mildly reduced and preserved ejection fraction: systematic review and network meta-analysis. JACC Heart Fail 12 (4), 616–627. 10.1016/j.jchf.2023.07.014 37656079

