# Supplementary material for: Machine-learning phenotyping and exploratory medication-outcome associations in heart failure with preserved ejection fraction: a multicohort retrospective study
Source: Front Pharmacol. 2026 Sep 28;17:1838338. doi: 10.3389/fphar.2026.1838338 (PMC13654846; doi:10.3389/fphar.2026.1838338)
Supplement: Supplementary file 1 [file DataSheet1.pdf]

Supplementary materials

Supplementary Methods

1. Cross-Cohort Variable Harmonization and Outcome Ascertainment

To ensure comparability across cohorts, variable definitions and measurement units were harmonized before data preprocessing and statistical analysis. The measurement units used in the MIMIC cohorts were adopted as the reference units. In the external application cohort, glucose was converted from mmol/L to mg/dL using the formula mmol/L × 18.02; serum creatinine was converted from µmol/L to mg/dL using µmol/L ÷ 88.4; blood urea nitrogen was converted from mmol/L to mg/dL using mmol/L ÷ 0.357; and total calcium was converted from mmol/L to mg/dL using mmol/L ÷ 0.25. The converted values were used consistently in phenotypic clustering, baseline comparisons, propensity-score analyses, and phenotype-classification analyses. The SQL parser retained explicit comparison operators: upper-bound expressions (< or ≤) were excluded from establishing LVEF ≥50%, whereas exact values and lower-bound expressions (> or ≥) were eligible only when the stated threshold was at least 50%.

For the MIMIC cohorts, mortality dates were obtained from the death records available in the corresponding databases. In the external application cohort, mortality status and date of death were ascertained through telephone follow-up. The variable status_365 was defined as a binary indicator of all-cause mortality within 365 days after ICU admission: patients who died within 365 days were coded as 1, whereas patients without a documented death within this period were coded as 0. For time-to-event analyses, a 24-hour landmark design was applied to prevent immortal time bias; therefore, follow-up time was calculated starting from 24 hours after ICU admission to death; patients without a documented death were censored at 365 days or at the date of last available follow-up, whichever occurred first. The same endpoint definition was applied across all three cohorts.

2. Cluster Selection, Stability Assessment, K = 2 Sensitivity Analysis, and Prevention of Information Leakage

Phenotypic clustering was performed using the K-prototypes algorithm implemented in the clustMixType package in R. Candidate solutions from K = 2 to K = 10 were evaluated using total within-cluster sum of squares and average silhouette width calculated from a Gower distance matrix. Candidate solutions from K = 2 to K = 6 were additionally evaluated for resampling stability and cluster-size distribution. The cluster-number decision integrated statistical separation, resampling stability, avoidance of very small clusters, and clinical interpretability of baseline profiles; medication exposure and survival outcomes were not used for this decision. The three-cluster solution was retained as the primary exploratory framework, and K = 2 was retained as a prespecified sensitivity analysis.

Cluster stability was assessed using 50 repeated subsampling iterations for each candidate solution from K = 2 to K = 6. In each iteration, 80% of patients were sampled without replacement, K-prototypes was refitted using 15 random initializations, and the resulting prototypes were used to reassign the complete derivation cohort. Agreement with the corresponding full-sample solution was quantified using the adjusted Rand index and summarized by the median and interquartile range. The final three-cluster model was fitted with 25 random starts,

a maximum of 100 iterations, and random seed 0406. K = 3 was retained as the main exploratory representation for clinical granularity, not because it was statistically superior to K = 2. The full clustering, baseline-profile, survival, and weighted medication analyses were repeated under the equally plausible lower-resolution K = 2 solution using the same specifications (Supplementary Figure 2 and Supplementary Table 5).

To prevent information leakage, the clustering feature set was restricted to clinical variables collected during the first 24 hours after ICU admission, as defined in the main Methods. Medication exposure variables, mortality status, survival time, and other follow-up information were not included in the clustering input, cluster-number selection, or stability assessment. UMAP was used only for two-dimensional visualization after clustering and did not contribute to cluster assignment.

3. Propensity-score specification, within-cluster diagnostics, and weighted survival models

For each medication class in the derivation and internal validation cohorts, a separate logistic propensity-score model was fitted using cluster membership, 14 prespecified covariates, and all cluster-by-covariate interaction terms. The covariates were age, sex, weight, heart rate, systolic blood pressure (SBP), peripheral oxygen saturation (SpO_2), creatinine, potassium, blood urea nitrogen (BUN), myocardial infarction, chronic pulmonary disease, renal disease, hypertension, and cardiac arrhythmias. Creatinine and BUN were log-transformed in the propensity-score models and balance diagnostics. This specification generated one set of medication-specific cohort weights while allowing the exposure model to vary by phenotype. Because the fully interacted specification could not be estimated reliably in the smaller external application cohort, the external analysis used cohort-level propensity-score models without cluster interactions only to provide descriptive weighted summaries and balance diagnostics; these estimates were not used for adjusted cluster-specific inference. Stabilized inverse probability of treatment weights were calculated from treatment prevalence and individual propensity scores and truncated at the empirical 1st and 99th percentiles. Overlap weights were calculated as one minus the propensity score for exposed patients and as the propensity score for unexposed patients; overlap weights were not truncated.

Covariate balance was evaluated before and after weighting both overall and within each cluster using absolute standardized mean differences, with ASMD < 0.10 considered acceptable. Empirical positivity was evaluated using raw propensity-score distributions and the proportion of extreme scores. Exposed and unexposed sample sizes, mortality-event counts, and effective sample sizes were summarized for every medication–cluster–cohort combination. The unified diagnostics are reported in Supplementary Table 4, with cluster-facetted propensity-score distributions for the derivation and internal validation cohorts in Supplementary Figure 16 and external overall-cohort distributions in Supplementary Figure 17. Because the external within-group balance criterion was not met in any of the nine cluster-specific comparisons, the corresponding medication estimates were treated as descriptive only.

All medication–phenotype analyses were exploratory. Overall and cluster-specific medication–mortality associations were estimated using Cox proportional hazards models. In the derivation and internal validation cohorts, cluster-specific hazard ratios and formal medication-by-cluster interaction *P* values were obtained from full-cohort interaction models using robust sandwich variance estimators. Unadjusted models were descriptive,

OW was the primary exploratory weighted approach, and sIPTW was a sensitivity analysis. Scaled Schoenfeld residual diagnostics for the corresponding full interaction models are reported in Supplementary Table 9. Because the interaction was retained, the medication main-effect term is conditional on the reference cluster. Benjamini–Hochberg correction was applied to the 12 medication coefficients within each cohort and model; interaction *P* values were reported separately. The same specifications were repeated under K = 2. In the external application cohort, weighted medication estimates, interaction *P* values, and model diagnostics were retained only for descriptive completeness. A simplified doubly robust external sensitivity analysis could not be estimated reliably because of sparse medication–cluster strata; no external medication result was used for causal or confirmatory inference.

4. TabPFN Classifier Development and Evaluation

4.1 Feature Selection and TabPFN Model Fitting

All feature-selection procedures were restricted to the MIMIC-IV derivation cohort to prevent information leakage. Recursive feature elimination (RFE) using a random forest classifier and 10-fold cross-validation was repeated across 100 bootstrap iterations. Candidate feature-set sizes of 8, 10, 12, 14, and 37 were evaluated. The 37-predictor model achieved the highest absolute cross-validated accuracy and Kappa. Among the deliberately reduced-input configurations (8–14 predictors), the 14-predictor model achieved the best performance. Because the intended research interface requires manual data entry, the 14-predictor configuration was selected as a pragmatic performance–input-burden compromise rather than as the globally performance-optimal subset. The retained predictors were anion gap, creatinine, blood urea nitrogen, chloride, respiratory rate, glucose, renal disease, peripheral oxygen saturation, heart rate, platelet count, mean blood pressure, calcium, hematocrit, and systolic blood pressure. The final phenotype-assignment classifier was constructed using TabPFN version 2.2.1 and trained on the complete derivation cohort (n = 2,511), with the derivation phenotype identities retained as the fixed target classes. The model was designed to reproduce the machine-derived labels rather than to predict mortality or treatment response.

4.2 Internal Validation and Phenotype-Label Alignment

For internal validation, the preprocessing and K-prototypes workflow was applied de novo to MIMIC-III (n = 1,524), independently of the fitted TabPFN classifier. Before classifier evaluation, the three MIMIC-III clusters were mapped one-to-one to the MIMIC-IV phenotype identities with the Hungarian algorithm. The assignment minimized a prespecified cluster-profile distance across the clinical variables shared by both clustering workflows: continuous cluster summaries were standardized using derivation-cohort location and scale, categorical summaries were represented by cluster-specific prevalences, and feature contributions were equally weighted. Classifier predictions, survival outcomes, and medication exposures were not components of the distance or assignment. The mapping was fixed before performance was calculated and yielded Cluster 1 to Cluster 1, Cluster 2 to Cluster 2, and Cluster 3 to Cluster 3. The fixed MIMIC-IV-trained classifier was not refitted in MIMIC-III; predicted probabilities were compared with the independently reclustered and Hungarian-aligned reference labels.

4.3 Classifier Evaluation and Robustness Analyses

Classifier performance was evaluated using complementary discrimination, classification, and calibration measures. Benchmarking included 10-fold cross-validation estimates with 95% confidence intervals for TabPFN, random forest, XGBoost, and LightGBM. All benchmark algorithms used the same 14 predictors, preprocessing steps, cross-validation folds, training/validation partitions, and evaluation metrics as TabPFN; only the learning algorithm and algorithm-specific hyperparameters differed. In internal validation, the fixed TabPFN classifier was evaluated against the independently reclustered and prespecified Hungarian-aligned reference labels using phenotype-specific one-versus-rest AUCs, confusion matrices, precision–recall curves, calibration curves, phenotype-specific Brier scores, and a 10-bin multiclass expected calibration error (ECE). For each patient, confidence was the maximum predicted class probability and correctness indicated whether the predicted class matched the aligned reference label. Predictions were partitioned into 10 equal-width confidence bins. ECE was calculated as $\Sigma(n\_b/N) \times |acc(b) - conf(b)|$, summed over $b = 1, \ldots, 10$, where n_b is the number of observations in bin b, acc(b) is the proportion correctly classified in that bin, and conf(b) is the mean maximum predicted probability in that bin. Single-feature ablation quantified changes in macro-F1 and phenotype-specific AUCs after removal of each input variable. Representative correct, incorrect, and low-confidence assignments were summarized with predicted probabilities and raw feature profiles. SHAP values were obtained from an auxiliary LightGBM classifier trained on the same variables and labels and therefore describe the surrogate model rather than direct TabPFN explanations.

4.4 Application to the External Cohort

Following cross-cohort harmonization and preprocessing, the external application cohort (n = 340) was evaluated using the fixed MIMIC-IV-trained TabPFN classifier. No de novo K-prototypes clustering, label alignment, retraining, or model updating was performed. Each patient was assigned to the label with the largest predicted probability. Because independently generated reference labels were unavailable, external classification AUC, accuracy, and related supervised metrics were not estimated. The external cohort was used primarily to explore classifier application, feature-distribution shift, missingness, prediction uncertainty, and the descriptive transportability of classifier-assigned phenotype profiles. Medication–mortality estimates were included only as secondary descriptive summaries and were not used to establish external treatment associations. Prediction uncertainty was summarized using the maximum predicted class probability and normalized entropy, defined as $-\Sigma p_k \log(p_k)/\log(3)$, where values closer to 0 indicate greater certainty and values closer to 1 indicate greater uncertainty. A maximum probability below 0.60 was displayed as a descriptive low-confidence threshold and was not treated as a validated rejection rule.

Supplementary Figures

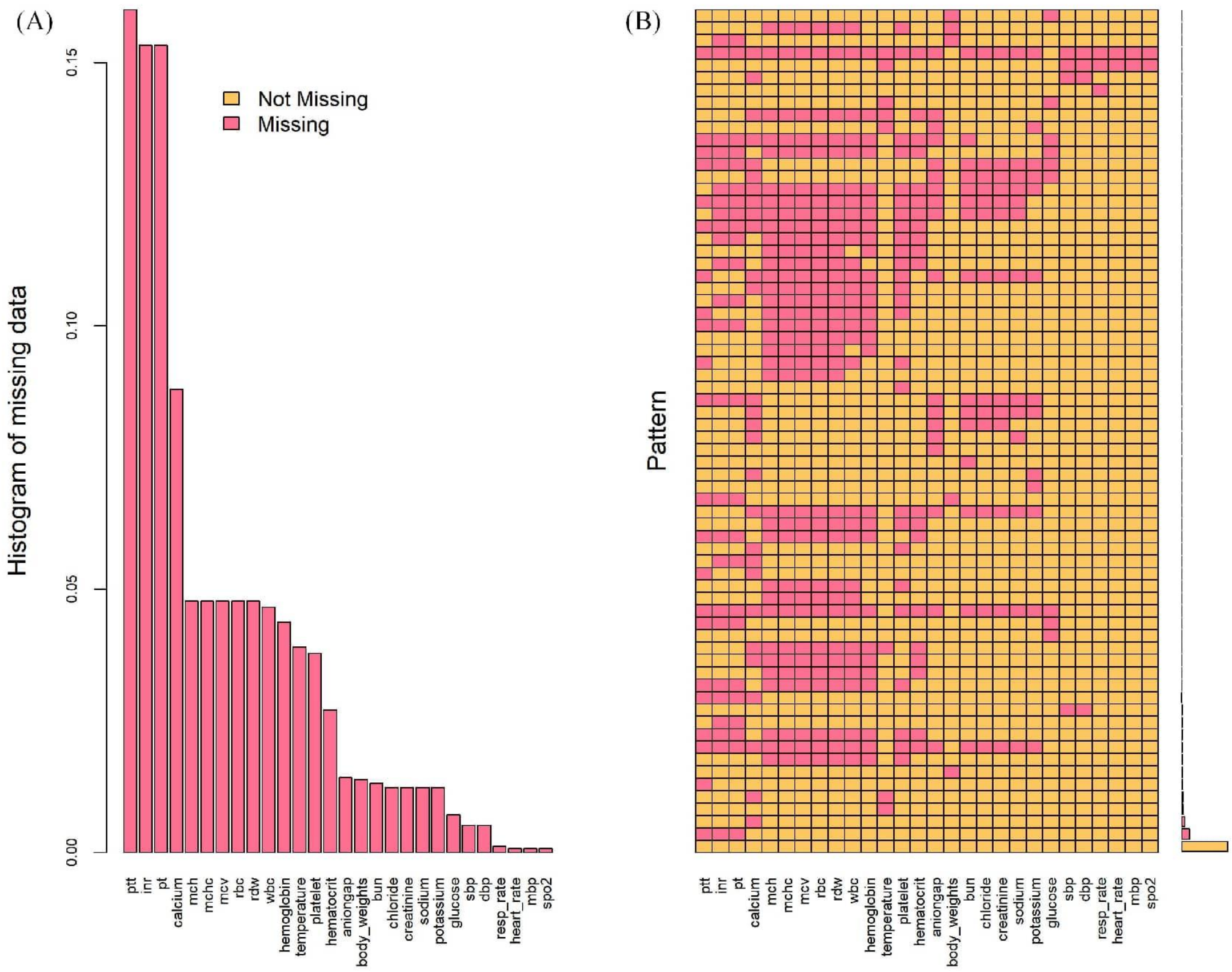

Supplementary Figure 1. Missing-data proportions and patterns among variables retained for analysis

(A) Variable-specific proportions of missing data among retained variables. (B) Observed missingness patterns across patients and variables. Variables with more than 30% missingness were excluded before imputation.

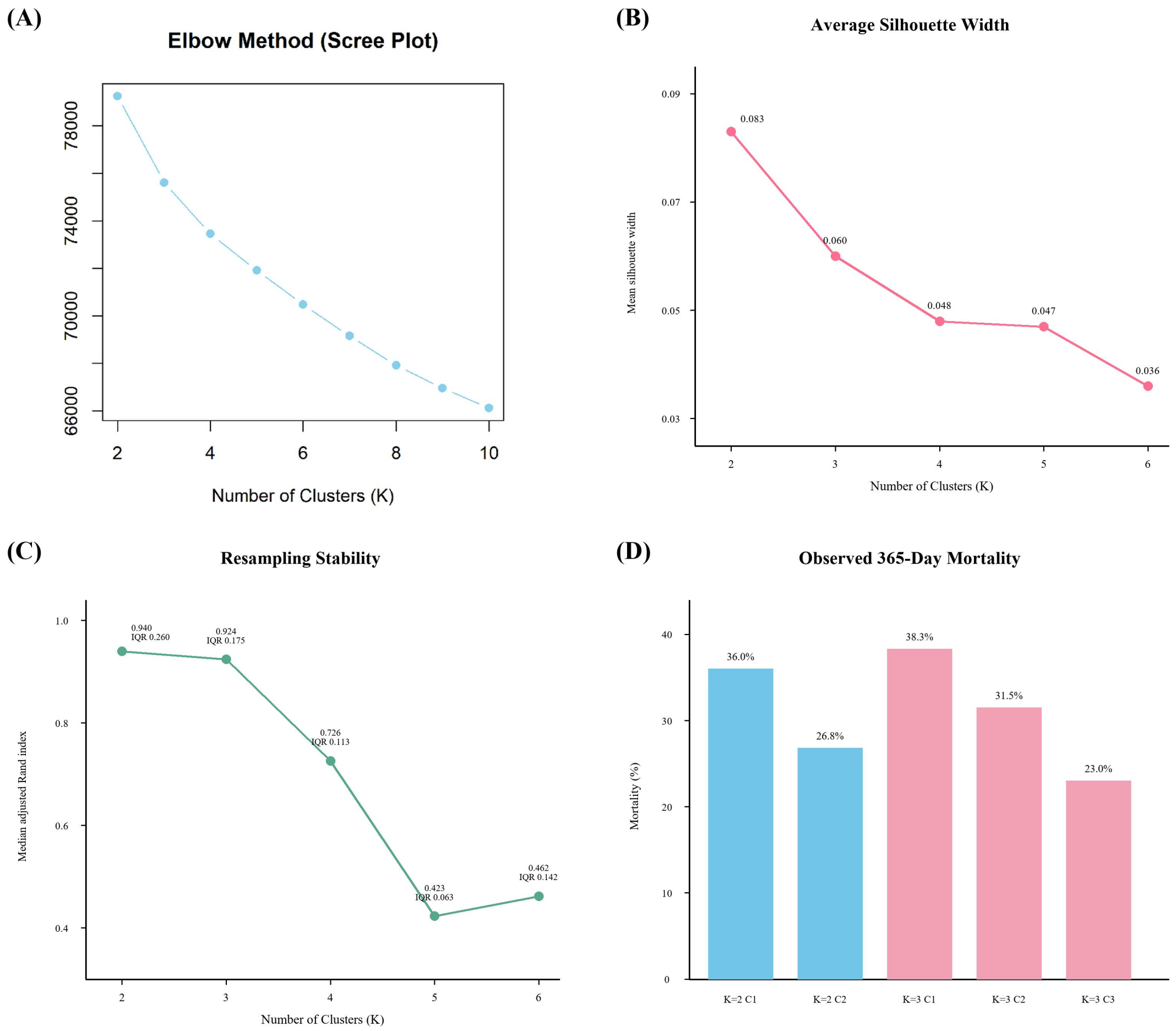

Supplementary Figure 2. Selection, stability, and descriptive resolution of candidate K-prototypes solutions

(A) Elbow plot of total within-cluster sum of squares across K = 2–10. (B) Mean silhouette width for K = 2–6 using the latest clustering evaluation. (C) Median adjusted Rand index and corresponding interquartile range across 50 subsampling iterations for K = 2–6. (D) Observed 365-day mortality proportions under the K = 2 and K = 3 solutions. K = 3 was retained as the main exploratory representation for clinical granularity, while K = 2 was treated as an equally plausible lower-resolution sensitivity solution; K = 3 was not considered statistically superior.

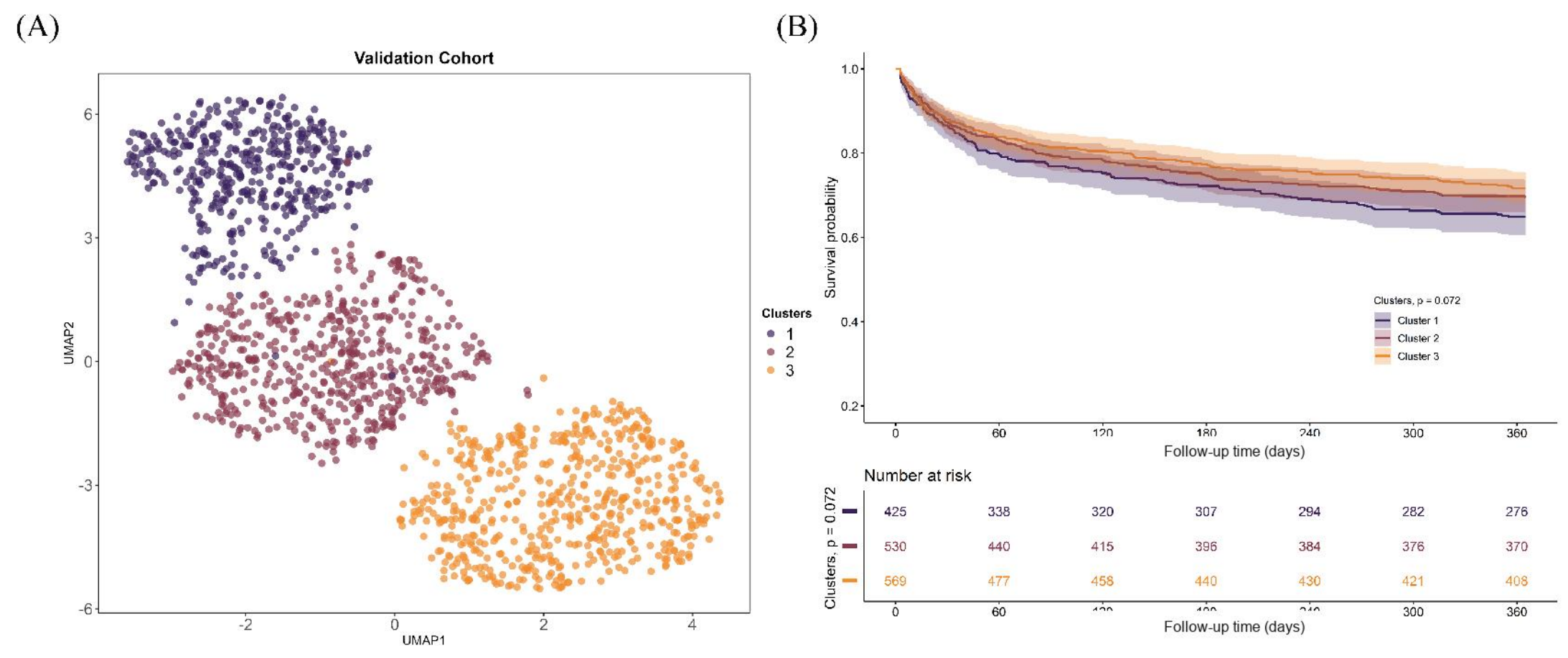

Supplementary Figure 3. Phenotypic clustering and associated survival outcomes in the internal validation cohort

(A) Uniform Manifold Approximation and Projection (UMAP) visualization showing the three phenotypic clusters in the internal validation cohort. (B) 365-day Kaplan–Meier survival curves showing a similar ordering of survival without a statistically significant overall between-cluster difference (log-rank *P* = 0.072).

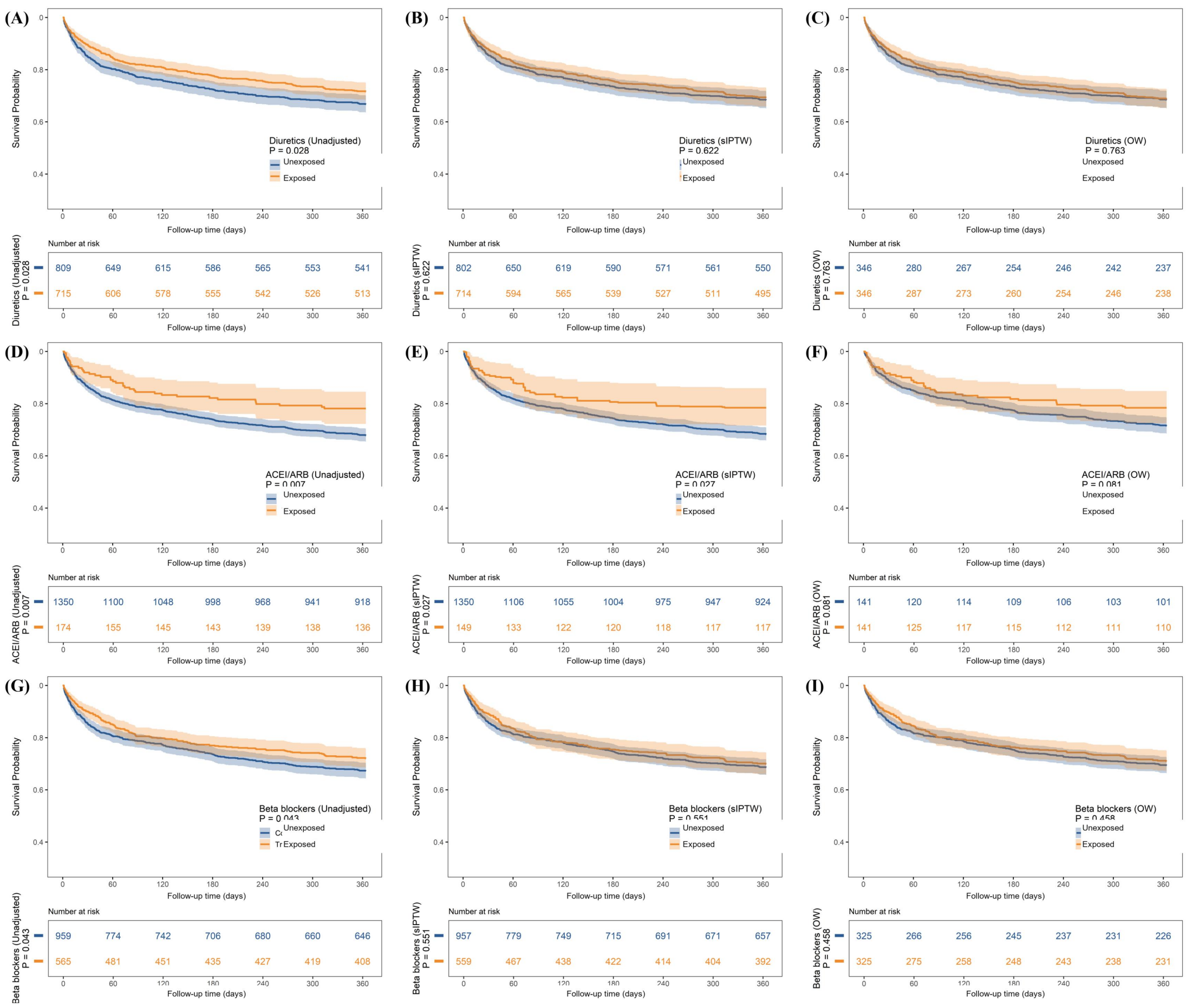

Supplementary Figure 4. Associations between early medication exposure and 365-day all-cause mortality in the internal validation cohort

KM curves illustrating the associations between early medication exposure and 365-day all-cause mortality under three analytical approaches: unadjusted (A, D, and G), sIPTW-adjusted (B, E, and H), and OW-adjusted (C, F, and I). *P* values in the unadjusted panels were calculated using the log-rank test, whereas *P* values in the weighted panels were derived from Wald tests of the corresponding weighted Cox proportional hazards models with robust variance estimators.

ACEI, angiotensin-converting enzyme inhibitor; ARB, angiotensin receptor blocker; sIPTW, stabilized inverse probability of treatment weighting; OW, overlap weighting.

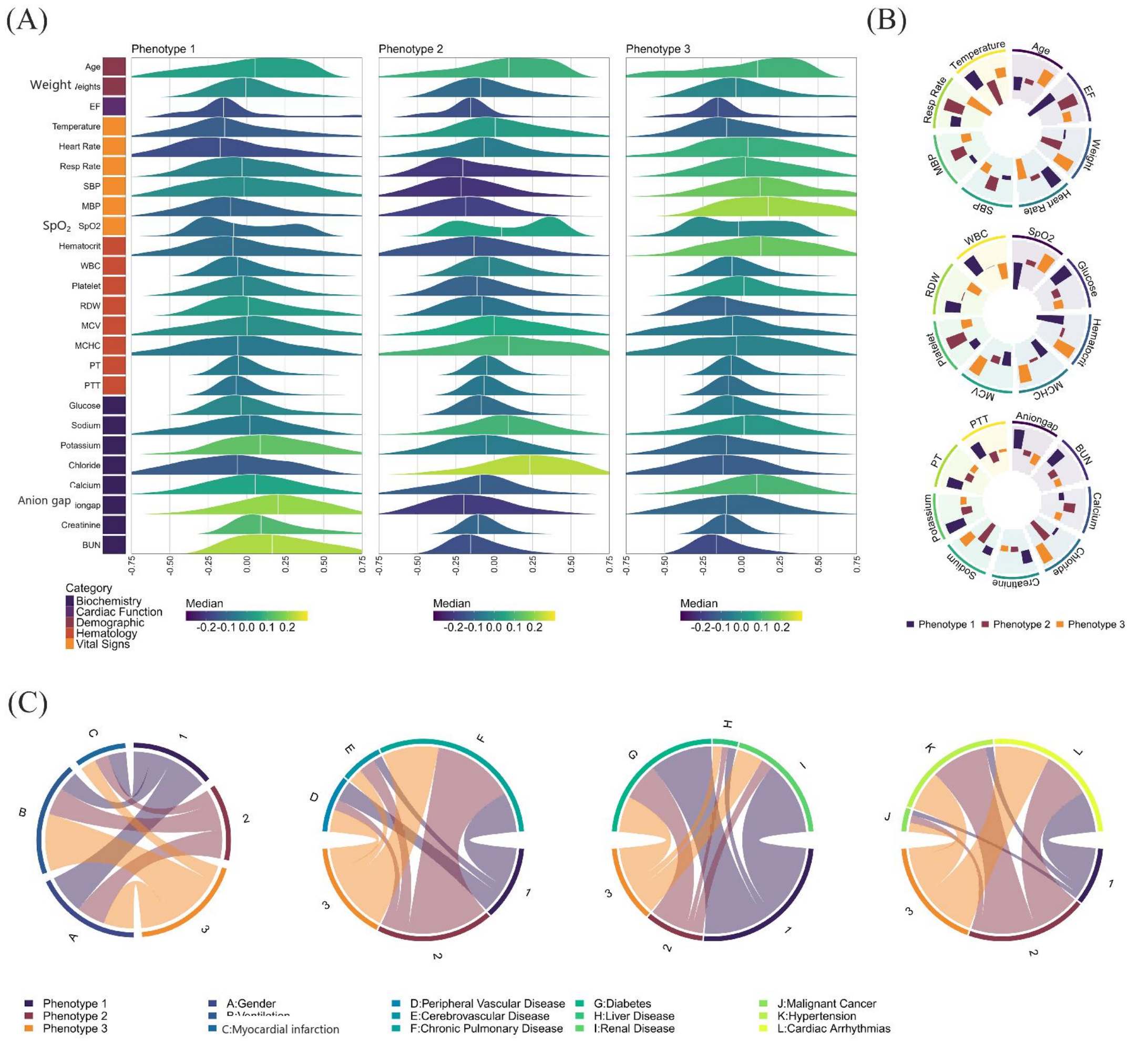

Supplementary Figure 5. Clinical characteristics of the internal validation cohort stratified by aligned phenotypic clusters

(A) Mountain plots showing the distribution of continuous clinical variables across the three clusters. Variables are color-coded by clinical domain, as indicated on the left. (B) Circular bar plot displaying cluster-specific mean standardized values after rescaling to the range [−1, 1]. (C) Chord diagram visualizing associations between phenotypic clusters and key comorbidities.

Abbreviations: BUN, blood urea nitrogen; SBP, systolic blood pressure; EF, ejection fraction; HR, heart rate; MBP, mean blood pressure; MCHC, mean corpuscular hemoglobin concentration; MCV, mean corpuscular volume; PT, prothrombin time; PTT, partial thromboplastin time; RDW, red cell distribution width; RR, respiratory rate; SpO_2, peripheral oxygen saturation; WBC, white blood cell count.

(A)

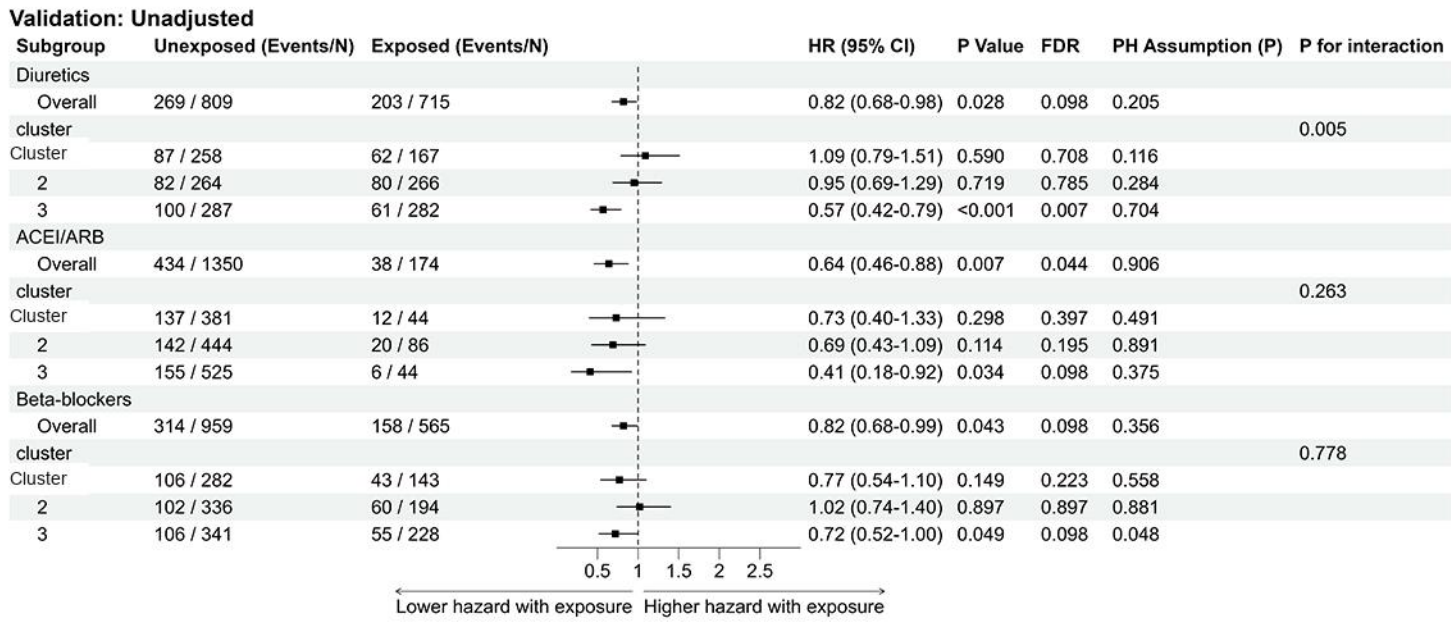

(B)

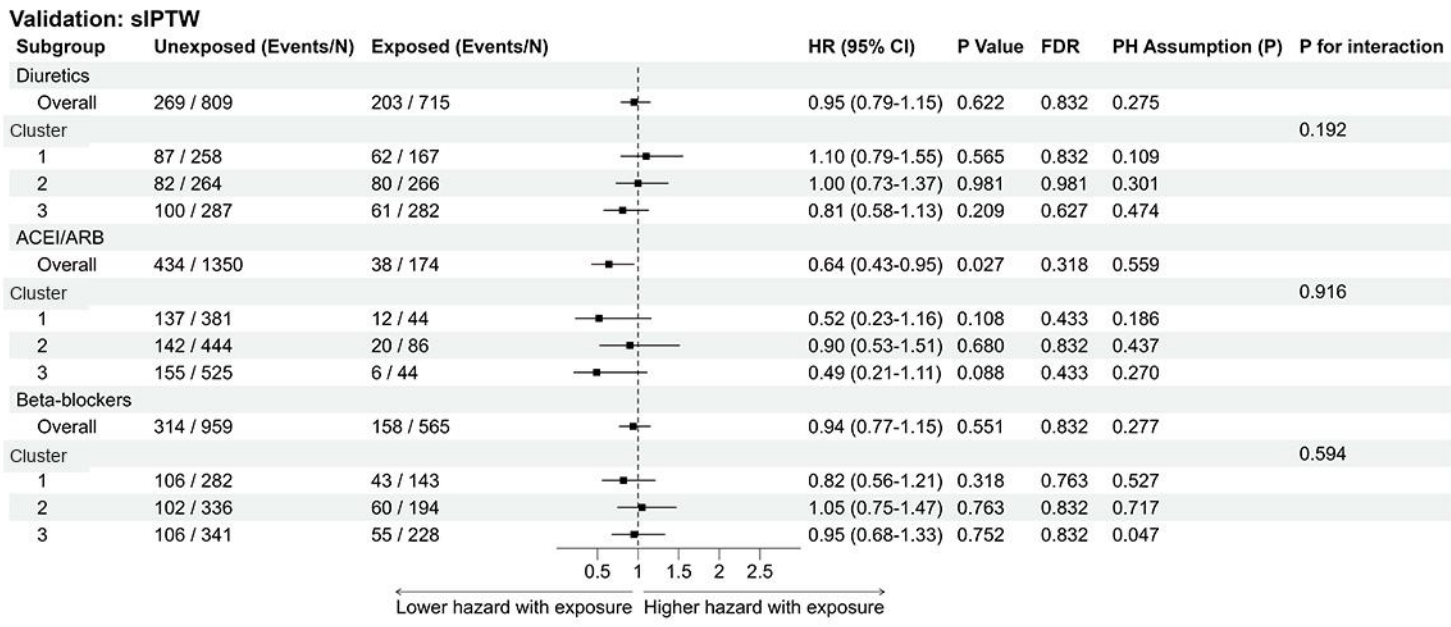

(C)

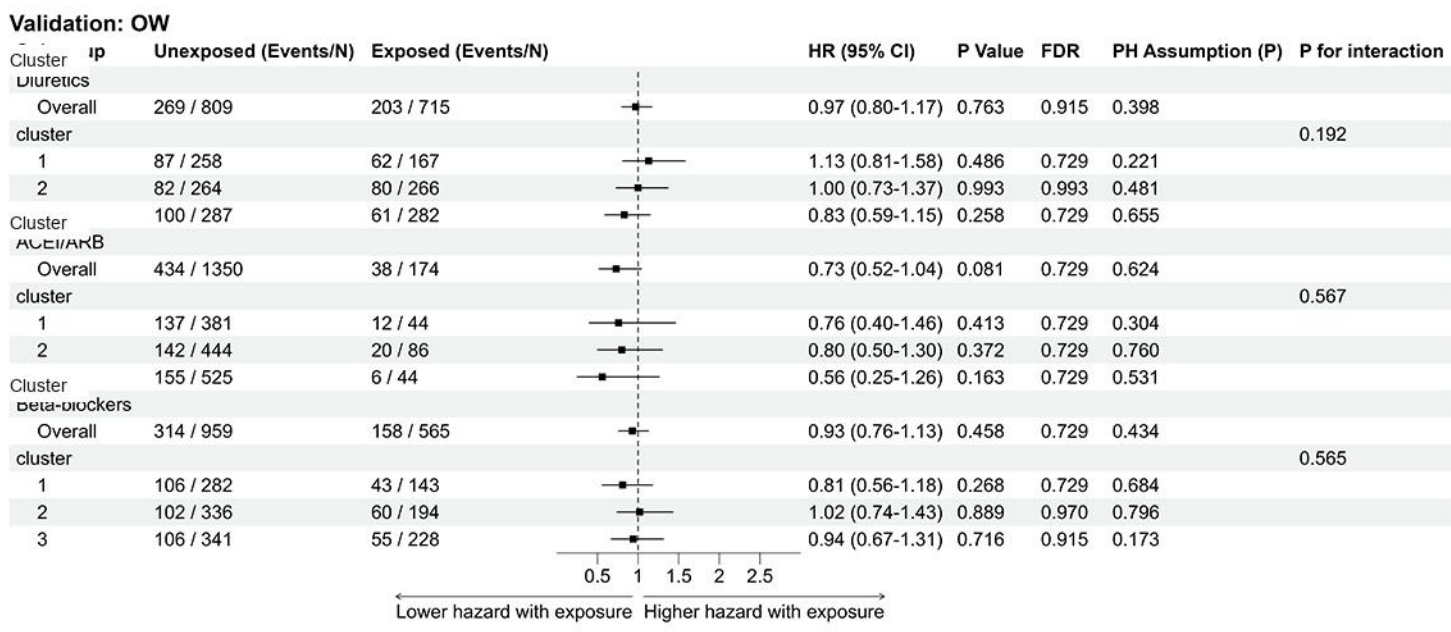

Supplementary Figure 6. Cluster-specific associations between medication exposure and 365-day mortality in the internal validation cohort

Forest plots show hazard ratios (HRs) and 95% confidence intervals (CIs) from (A) unadjusted, (B) stabilized inverse probability of treatment weighting (sIPTW), and (C) overlap weighting (OW) models. All medication–phenotype analyses were exploratory. Results are shown overall and within each phenotype for diuretics, ACEI/ARB, and beta-blockers. Columns report unexposed and exposed event/sample counts, HRs with 95% CIs, raw *P* values, Benjamini–Hochberg FDR-adjusted *P* values, proportional-hazards test *P* values from the corresponding overall or cluster-specific medication models, and medication-by-cluster interaction *P* values. Full interaction-model term-specific and global proportional-hazards diagnostics are reported in Supplementary Table 9.

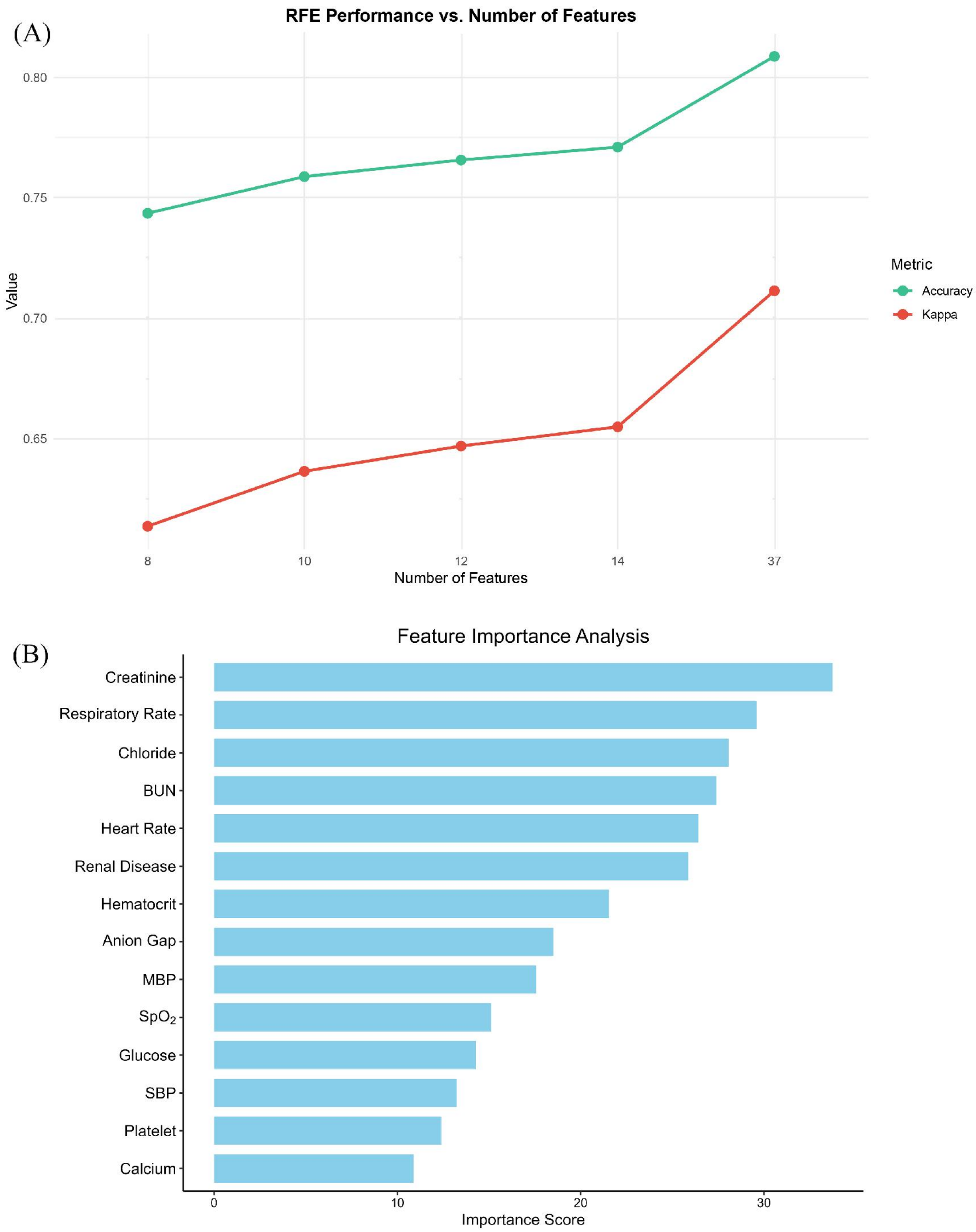

Supplementary Figure 7. Recursive feature elimination performance and the selected reduced-input configuration

(A) Cross-validated accuracy and Kappa across candidate feature-set sizes. The 37-variable model achieved the highest absolute performance; among the reduced-input configurations, 14 variables performed best and were retained to reduce manual input burden. This choice represents a pragmatic performance–input-burden compromise, not a claim that 14 variables were globally performance-optimal. (B) Random-forest feature-importance ranking for the 14 retained predictors.

Abbreviations: BUN, blood urea nitrogen; SpO_2, peripheral oxygen saturation; SBP, systolic blood pressure; MBP, mean blood pressure.

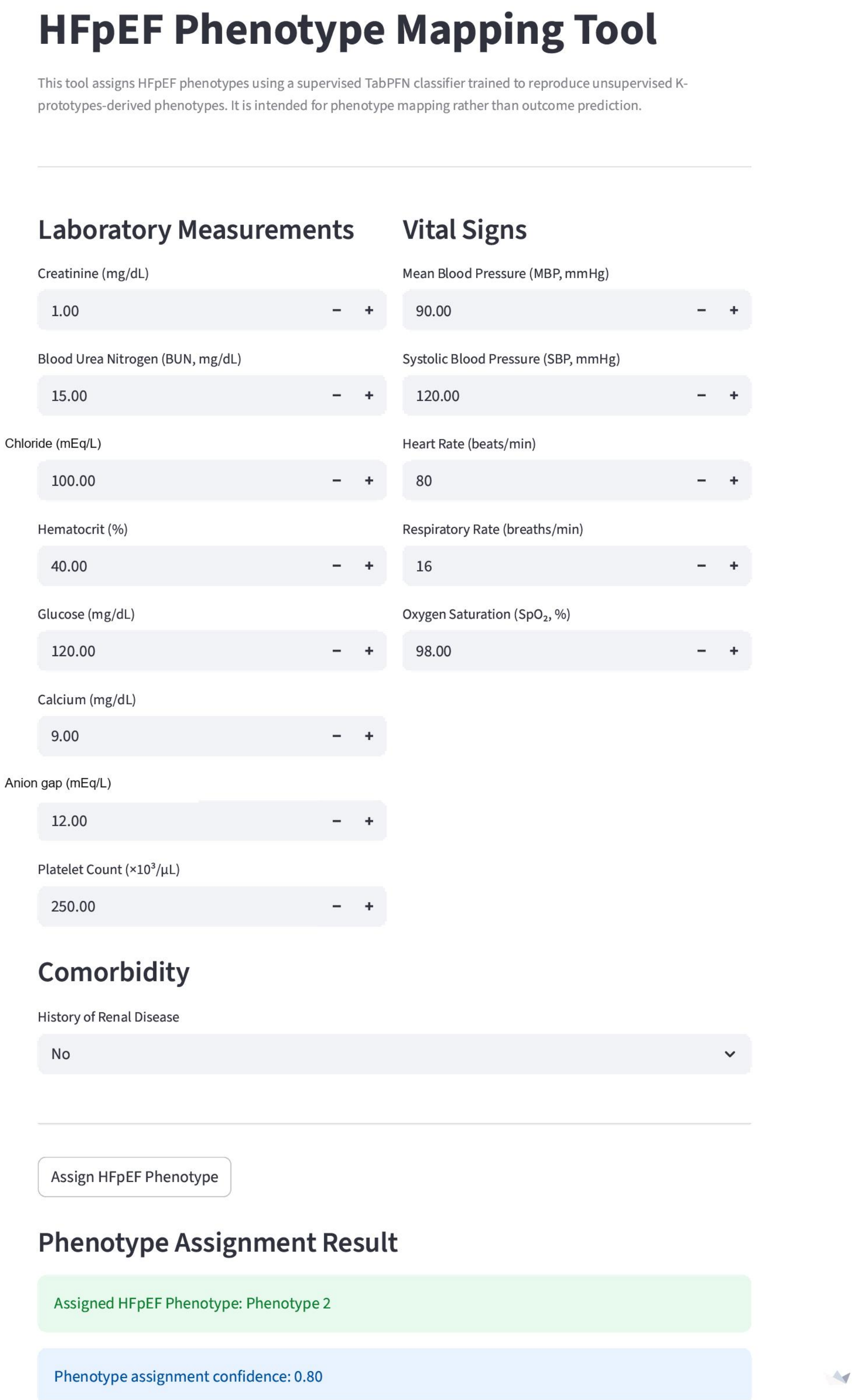

Supplementary Figure 8. Web-based HFpEF phenotype-assignment interface

The screenshot illustrates the research interface that applies the TabPFN classifier to assign patients to one of the three machine-derived HFpEF phenotypes. The tool is intended for research phenotype assignment and not for clinical decision support.

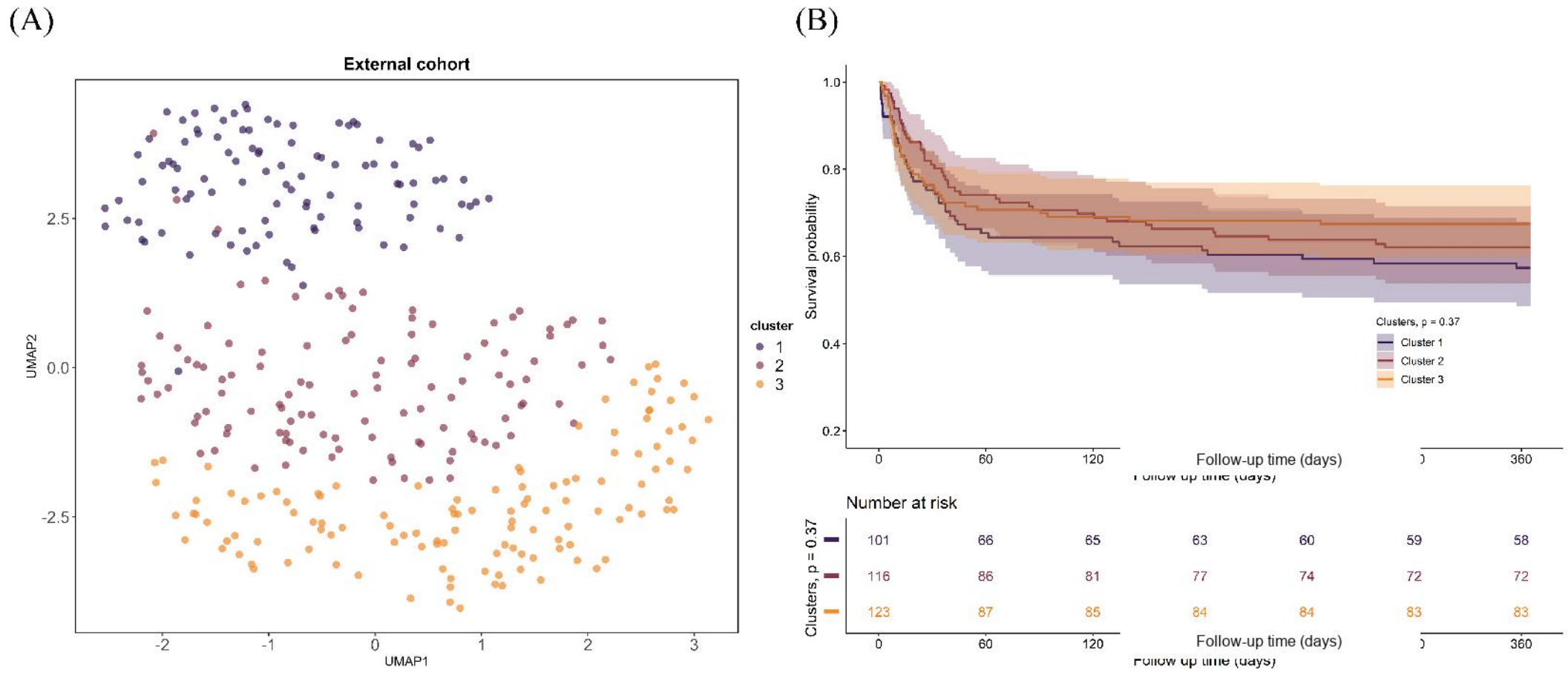

Supplementary Figure 9. Classifier-assigned phenotypes and associated survival outcomes in the external application cohort

(A) Uniform Manifold Approximation and Projection (UMAP) visualization of the classifier-assigned phenotypes in the external application cohort. (B) 365-day Kaplan–Meier survival curves showing a similar ordering of survival without significant between-cluster separation (log-rank *P* = 0.37).

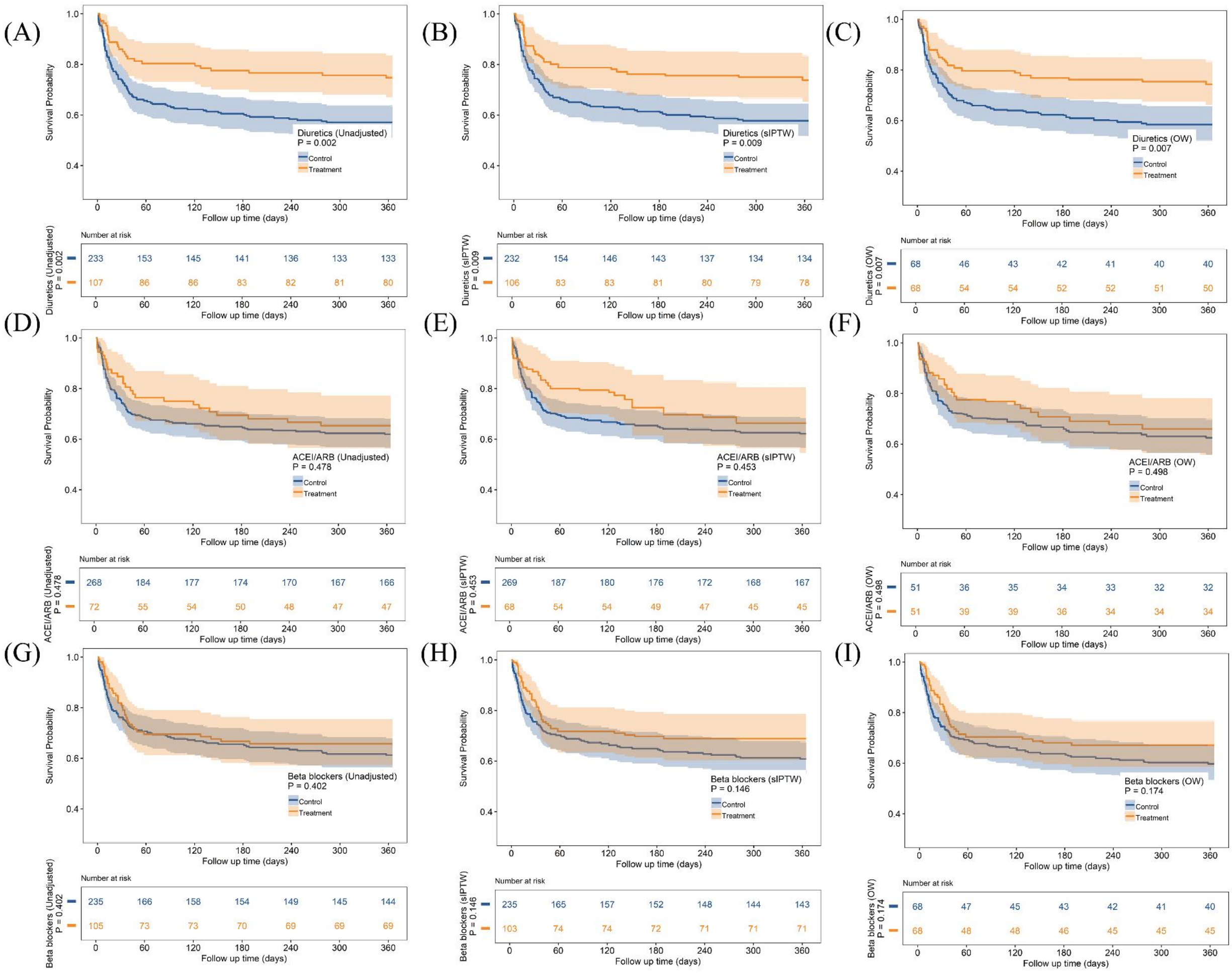

Supplementary Figure 10. Associations between early medication exposure and 365-day all-cause mortality in the external application cohort

KM curves illustrate descriptive associations between early medication exposure and 365-day all-cause mortality under three approaches: unadjusted, stabilized inverse probability of treatment weighting (sIPTW), and overlap weighting (OW). In this legacy low-resolution composite, the plot labels “Control” and “Treatment” denote the unexposed and exposed groups, respectively.

ACEI, angiotensin-converting enzyme inhibitor; ARB, angiotensin receptor blocker; sIPTW, stabilized inverse probability of treatment weighting; OW, overlap weighting.

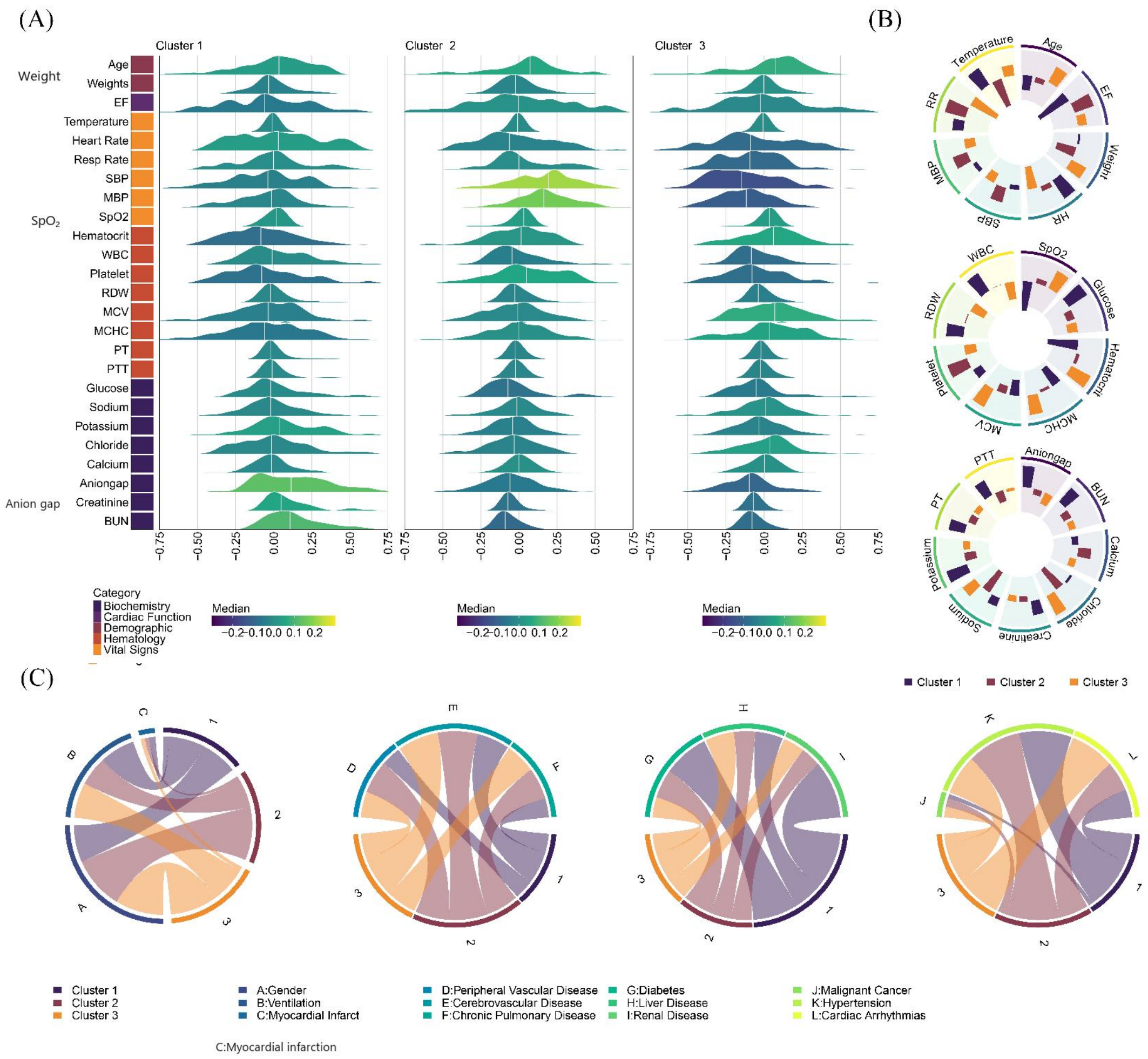

Supplementary Figure 11. Clinical characteristics of the external application cohort stratified by classifier-assigned phenotypes

(A) Mountain plots showing the distribution of continuous clinical variables across the three clusters. Variables are color-coded by clinical domain, as indicated on the left. (B) Circular bar plot displaying cluster-specific mean standardized values after rescaling to the range [−1, 1]. (C) Chord diagram visualizing associations between phenotypic clusters and key comorbidities.

Abbreviations: BUN, blood urea nitrogen; SBP, systolic blood pressure; EF, ejection fraction; HR, heart rate; MBP, mean blood pressure; MCHC, mean corpuscular hemoglobin concentration; MCV, mean corpuscular volume; PT, prothrombin time; PTT, partial thromboplastin time; RDW, red cell distribution width; RR, respiratory rate; SpO_2, peripheral oxygen saturation; WBC, white blood cell count.

(A)

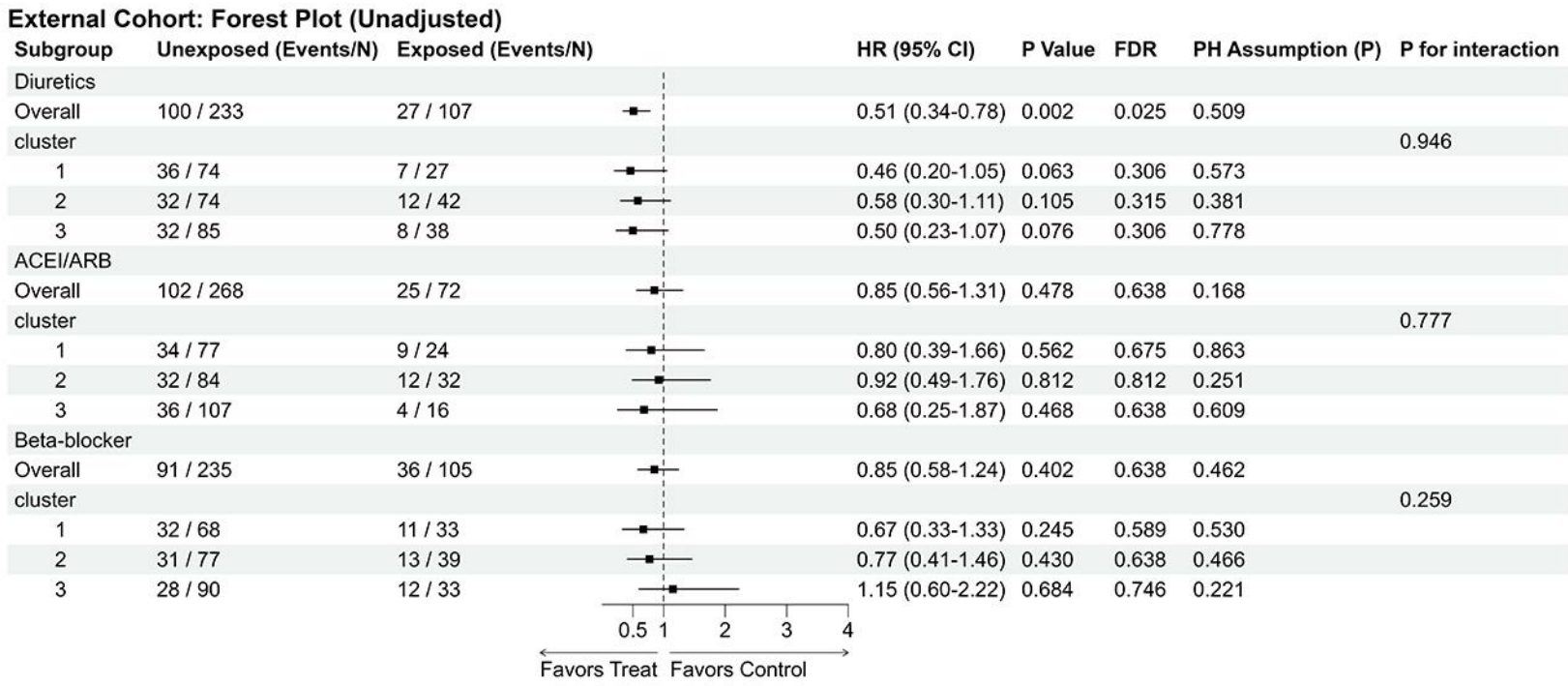

(B)

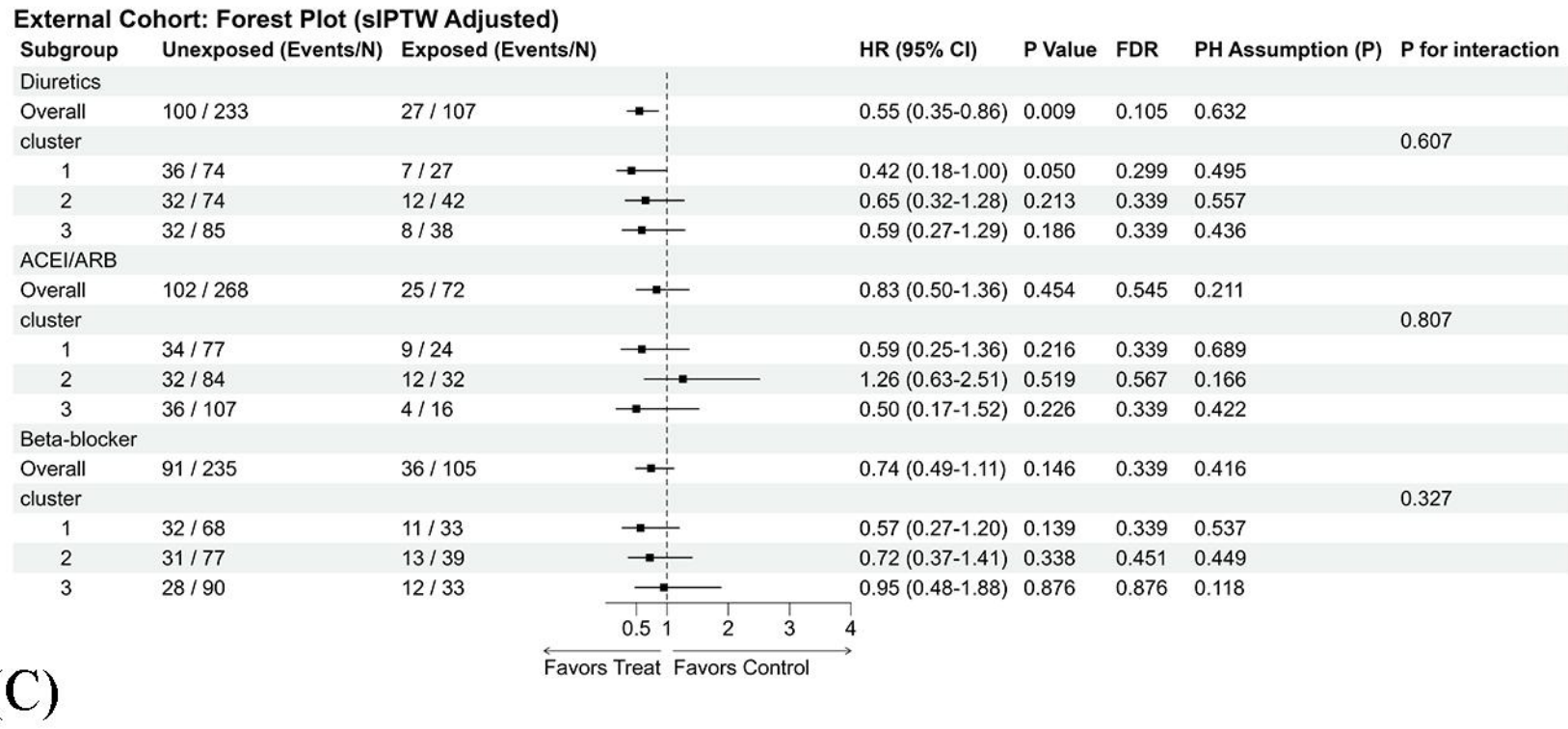

(C)

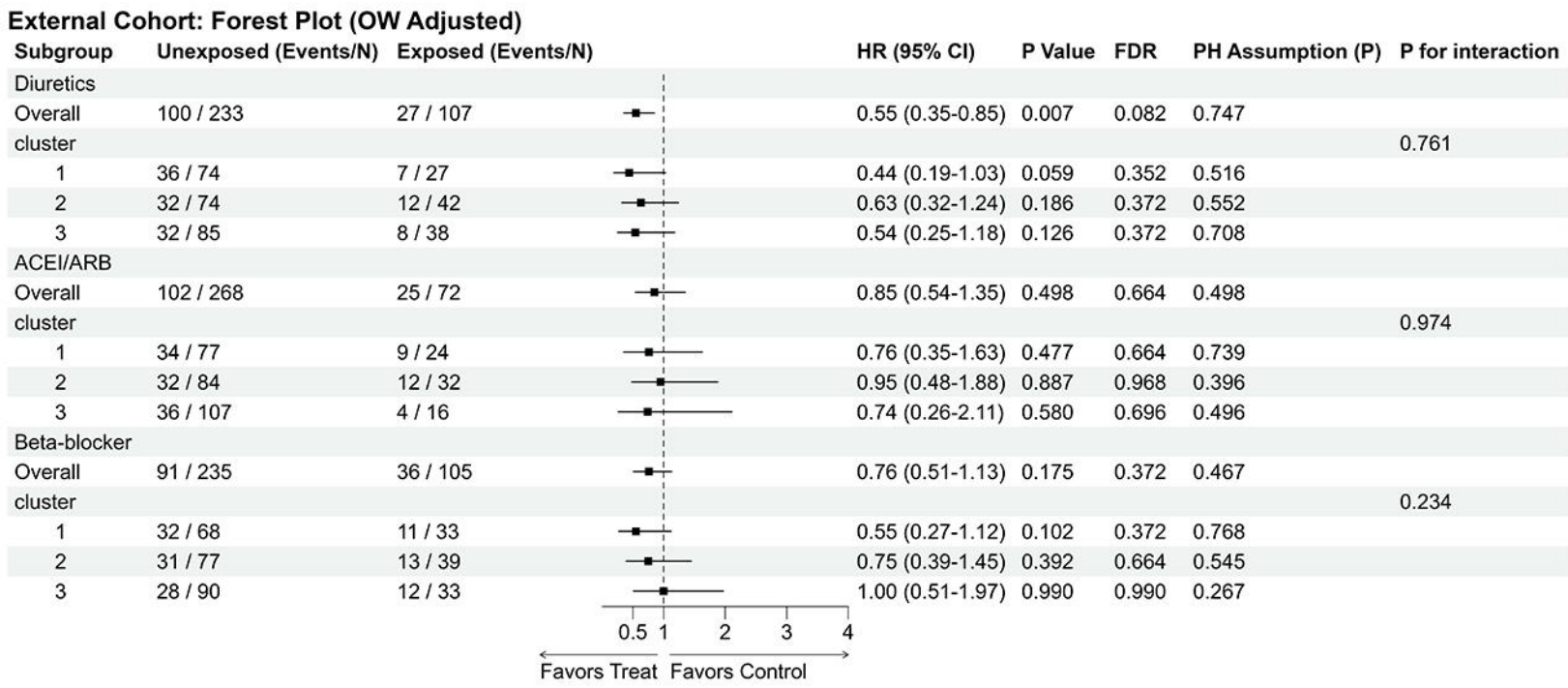

Supplementary Figure 12. Cluster-specific associations between medication exposure and 365-day mortality in the external application cohort

Forest plots show hazard ratios (HRs) and 95% confidence intervals (CIs) from (A) unadjusted, (B) stabilized inverse probability of treatment weighting (sIPTW), and (C) overlap weighting (OW) models. Results are shown overall and within each classifier-assigned phenotype. Columns report unexposed and exposed event/sample counts, HRs with 95% CIs, raw *P* values, Benjamini–Hochberg FDR-adjusted *P* values, proportional-hazards test *P* values, and medication-by-cluster interaction *P* values. External weights were estimated from cohort-level propensity-score models without cluster interactions, and all nine cluster-specific comparisons retained residual imbalance. These external estimates and tests are therefore presented only as exploratory descriptive summaries and were not used for adjusted, causal, or confirmatory inference.

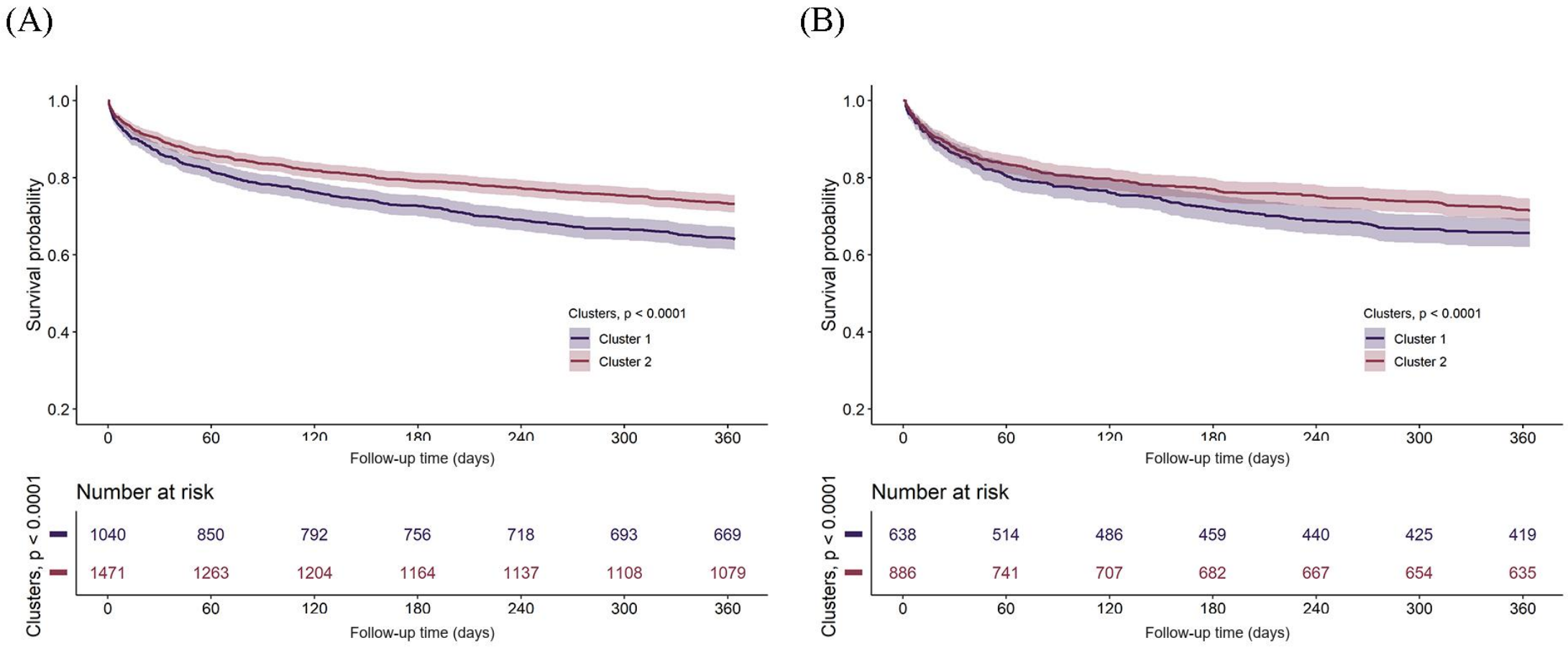

Supplementary Figure 13. Survival outcomes under the two-cluster sensitivity solution

(A) Kaplan–Meier survival curves for the two-cluster solution in the MIMIC-IV derivation cohort (n = 2,511; log-rank *P* < 0.0001). (B) Kaplan–Meier survival curves for the two-cluster solution in the MIMIC-III internal validation cohort (n = 1,524; log-rank *P* < 0.0001). Risk tables are shown beneath each panel.

(A)

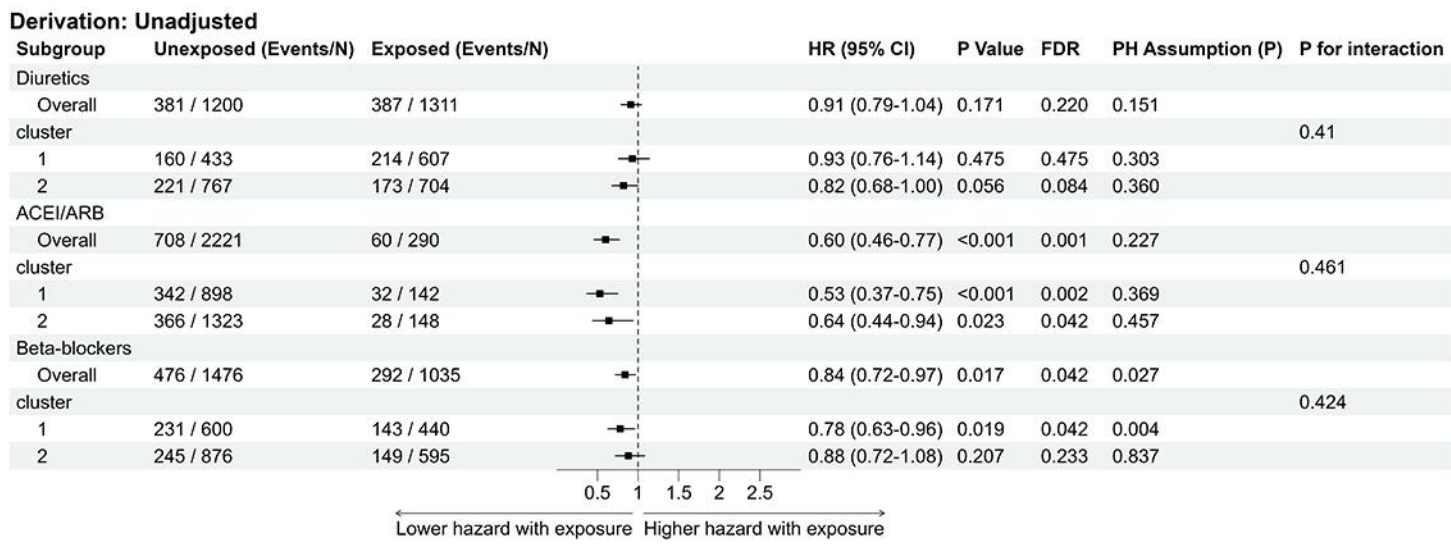

(B)

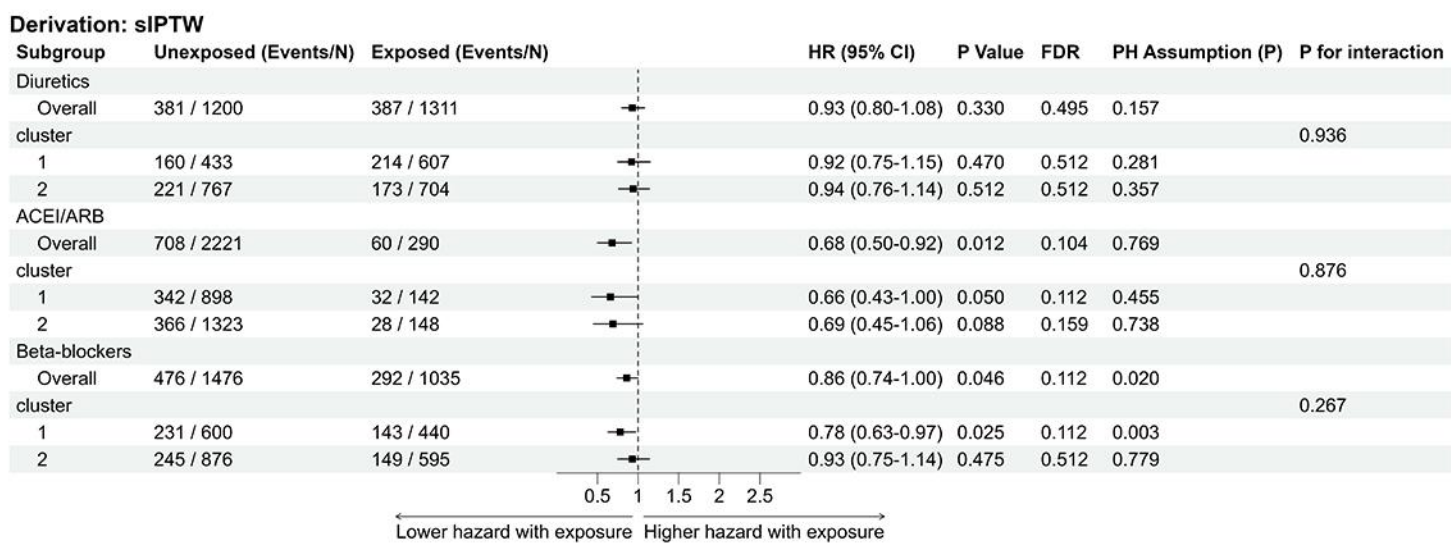

(C)

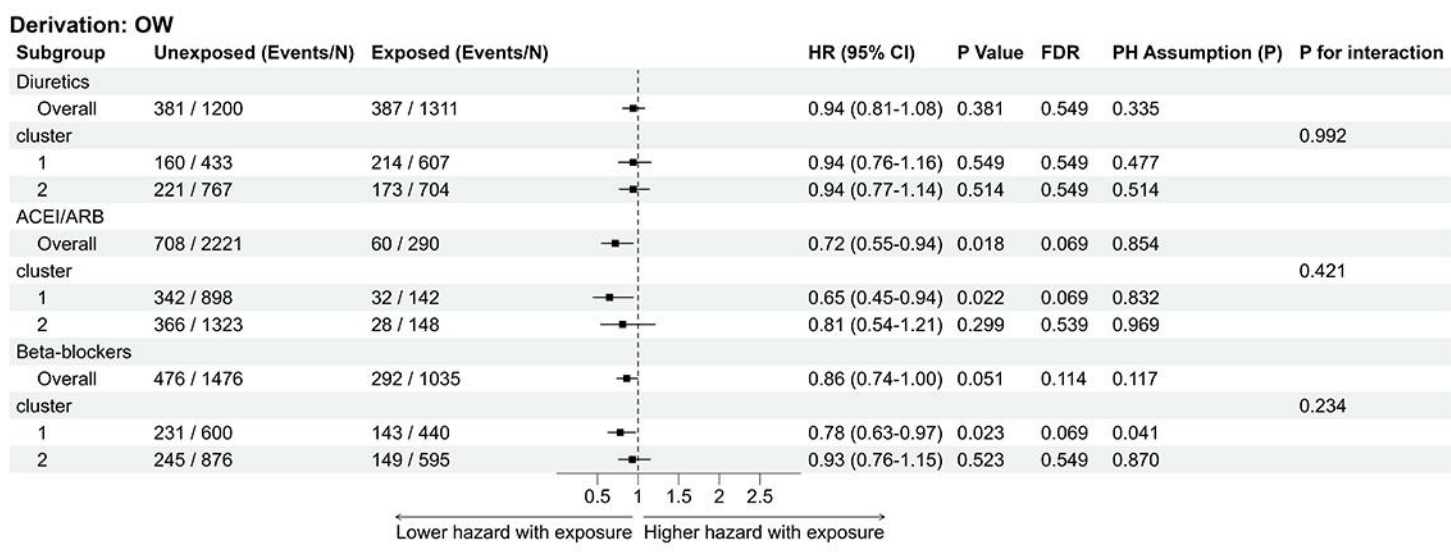

Supplementary Figure 14. Medication–mortality associations under the two-cluster solution in the derivation cohort

Forest plots show (A) unadjusted, (B) sIPTW, and (C) OW estimates overall and within the two sensitivity-analysis clusters. All medication–phenotype analyses were exploratory. Columns report event/sample counts, HRs with 95% CIs, raw and FDR-adjusted *P* values, proportional-hazards test *P* values, and medication-by-cluster interaction *P* values. Unadjusted estimates are descriptive; within the exploratory weighted analyses, OW is the primary approach and sIPTW is a sensitivity analysis.

ACEI, angiotensin-converting enzyme inhibitor; ARB, angiotensin receptor blocker; CI, confidence interval; FDR, false discovery rate; HR, hazard ratio; OW, overlap weighting; sIPTW, stabilized inverse probability of treatment weighting.

(A)

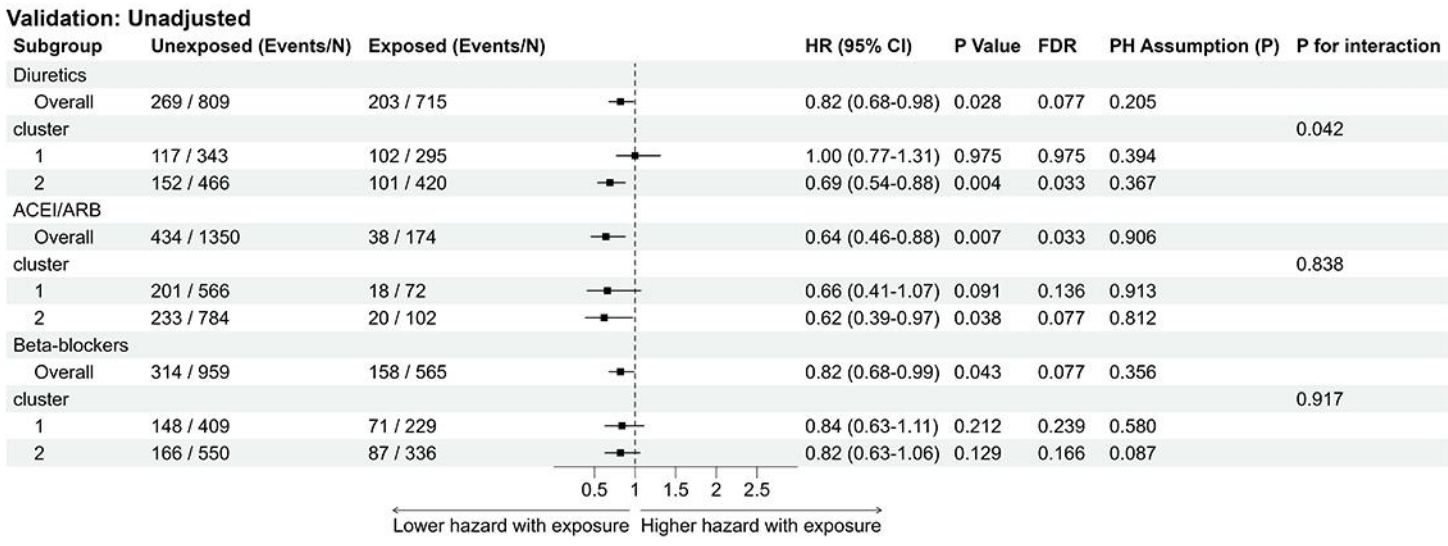

(B)

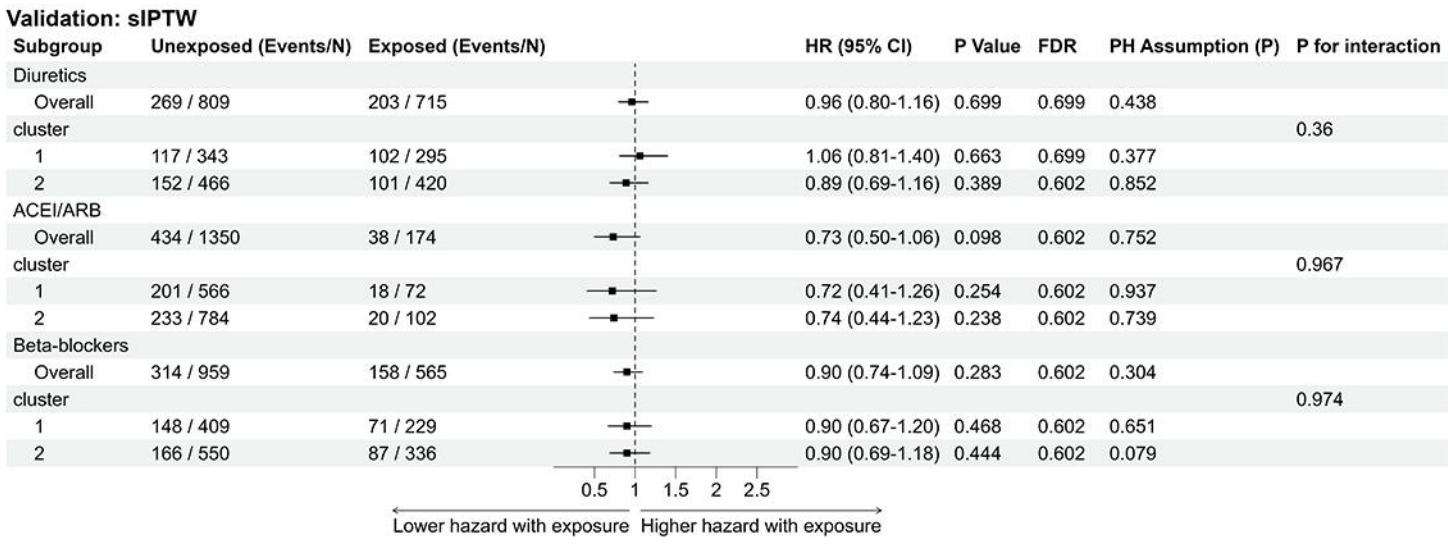

(C)

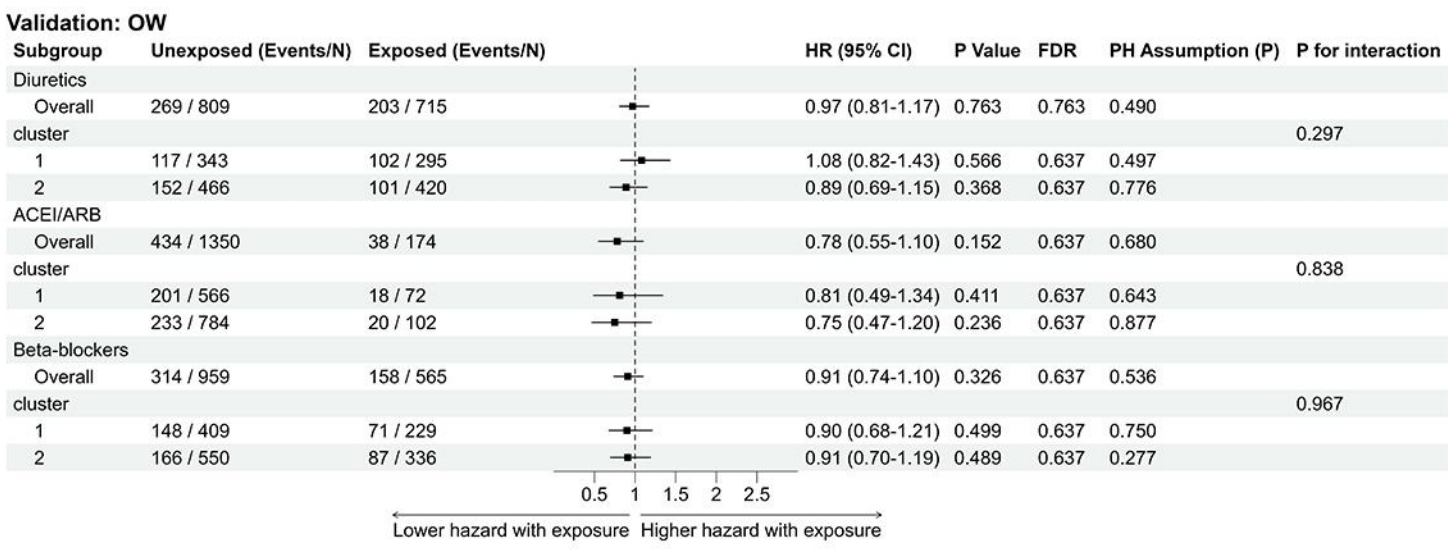

Supplementary Figure 15. Medication–mortality associations under the two-cluster solution in the internal validation cohort

Forest plots show (A) unadjusted, (B) sIPTW, and (C) OW estimates overall and within the two sensitivity-analysis clusters. All medication–phenotype analyses were exploratory. Columns report event/sample counts, HRs with 95% CIs, raw and FDR-adjusted *P* values, proportional-hazards test *P* values, and medication-by-cluster interaction *P* values. Within the exploratory weighted analyses, OW is the primary approach and sIPTW is a sensitivity analysis.

ACEI, angiotensin-converting enzyme inhibitor; ARB, angiotensin receptor blocker; CI, confidence interval; FDR, false discovery rate; HR, hazard ratio; OW, overlap weighting; sIPTW, stabilized inverse probability of treatment weighting.

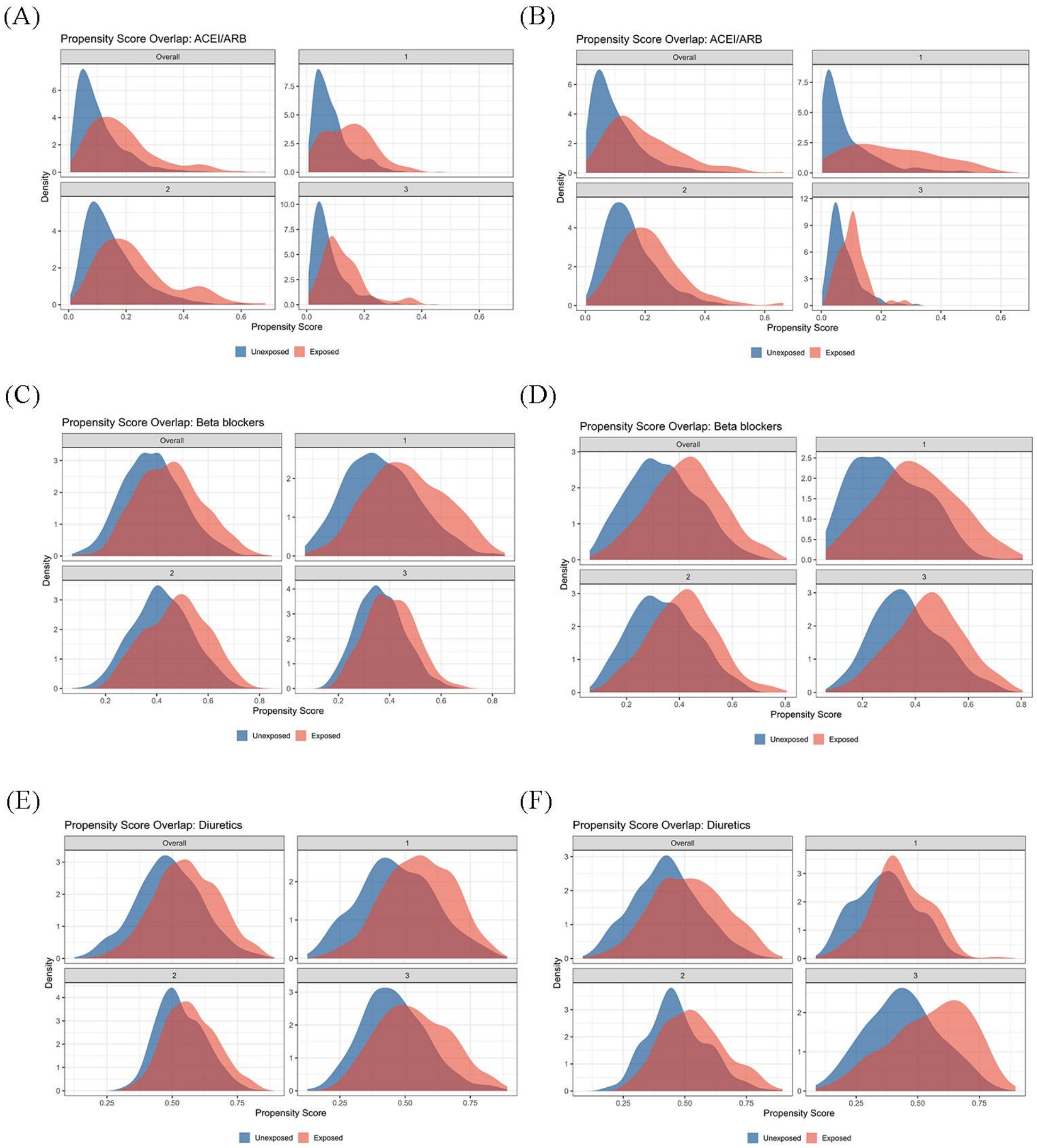

Supplementary Figure 16. Overall and within-cluster propensity-score overlap in the derivation and internal validation cohorts

Kernel density plots show raw propensity-score distributions for unexposed and exposed patients overall and within Clusters 1–3. Panels A, C, and E show the derivation cohort for ACEI/ARB, beta-blockers, and diuretics, respectively; panels B, D, and F show the corresponding internal validation analyses. Propensity-score models included cluster membership and cluster-by-covariate interaction terms.

ACEI, angiotensin-converting enzyme inhibitor; ARB, angiotensin receptor blocker.

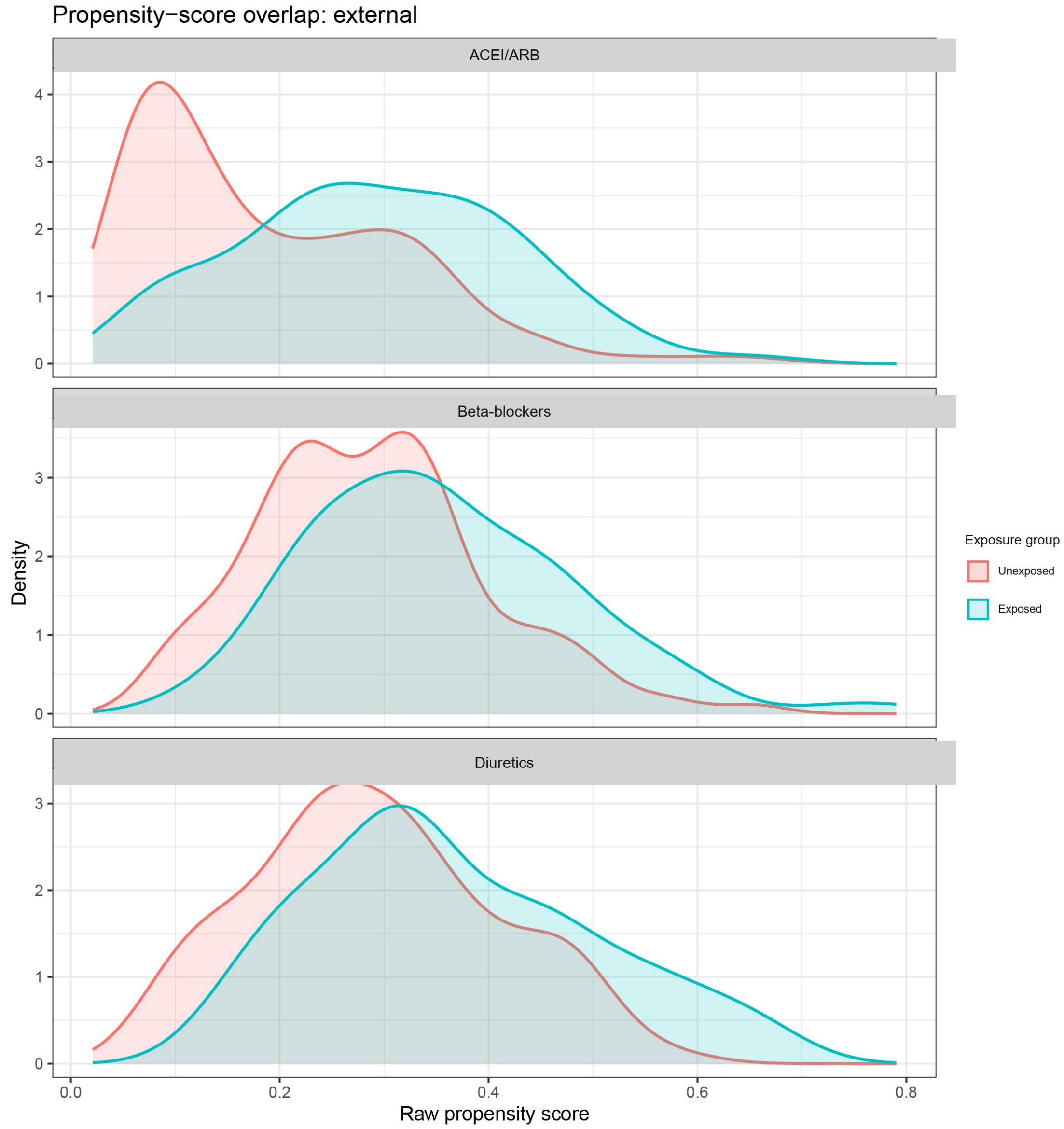

Supplementary Figure 17. Overall-cohort propensity-score overlap in the external application cohort

Kernel density plots show raw propensity-score distributions for unexposed (red) and exposed (teal) patients for ACEI/ARB, beta-blockers, and diuretics. External propensity scores were estimated using cohort-level medication-specific models without cluster interactions; within-cluster balance is summarized in Supplementary Table 4. These plots assess overall common support only. Because all nine external cluster-specific comparisons retained residual imbalance, the corresponding medication estimates were treated as descriptive and were not used for confirmatory inference.

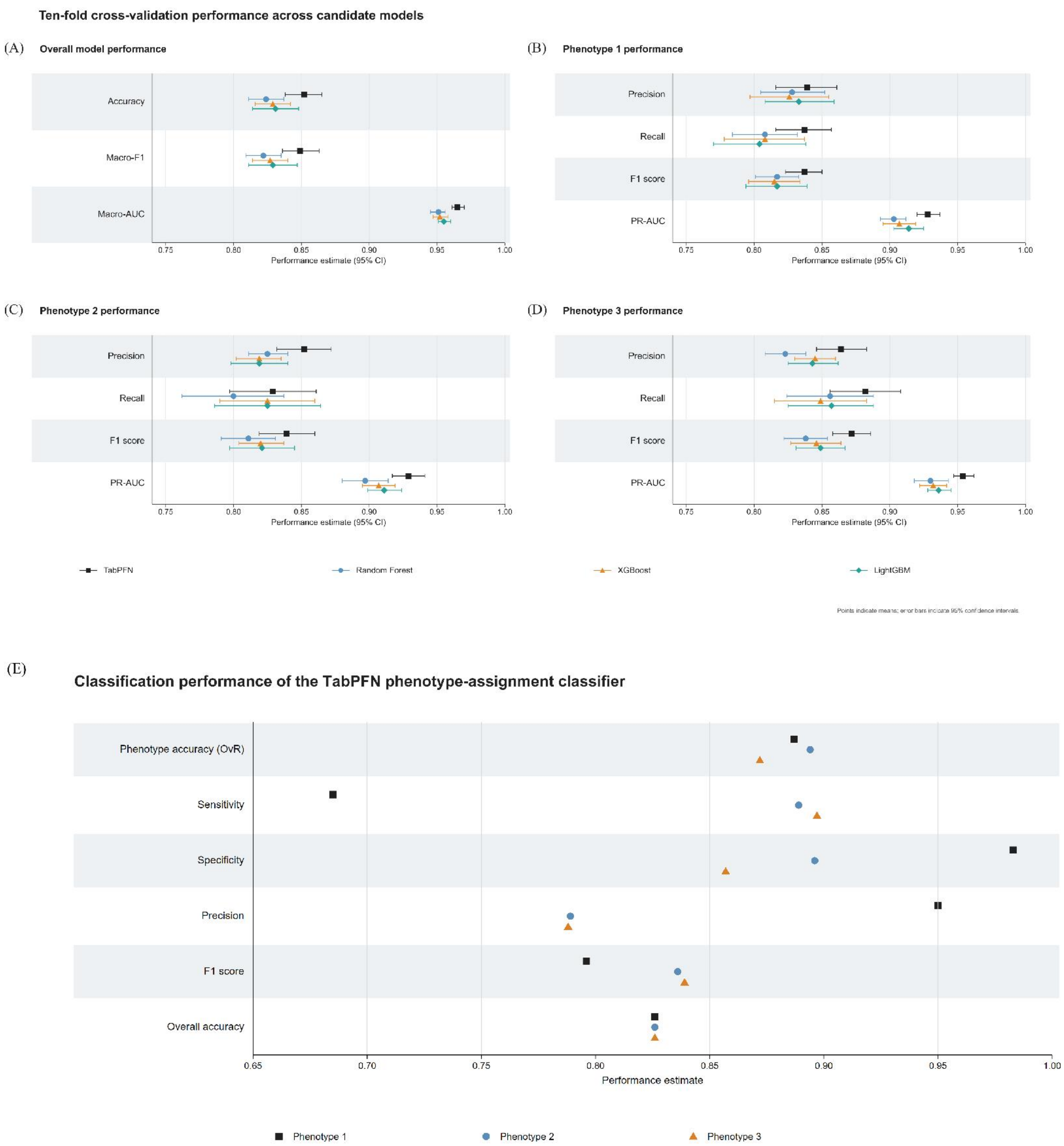

Supplementary Figure 18. Model benchmarking and phenotype-specific classification performance

(A–D) Ten-fold cross-validation estimates with 95% confidence intervals for TabPFN, random forest, XGBoost, and LightGBM: (A) overall accuracy, macro-F1 score, and macro-AUC; (B–D) precision, recall, F1 score, and PR-AUC for Phenotypes 1–3, respectively. All algorithms used the same 14 predictors, preprocessing steps, cross-validation folds, training/validation partitions, and evaluation metrics. (E) Internal-validation performance of the TabPFN classifier by phenotype, including one-versus-rest phenotype accuracy, sensitivity, specificity, precision, F1 score, and overall accuracy. AUC, area under the receiver operating characteristic curve; PR-AUC, area under the precision–recall curve; TabPFN, Tabular Prior-Data Fitted Network.

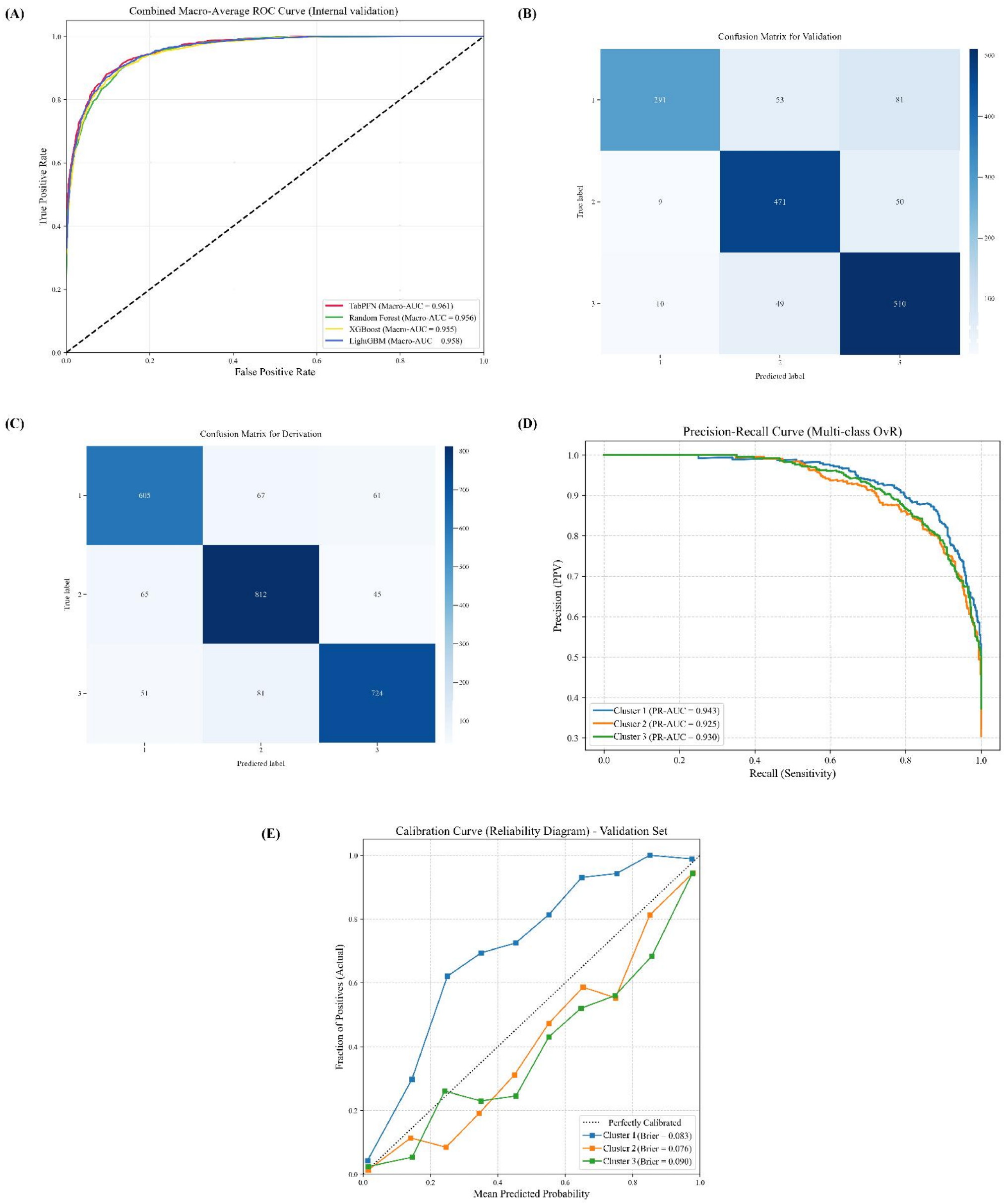

Supplementary Figure 19. Classification performance of the TabPFN phenotype classifier

(A) Macro-average receiver operating characteristic curves in the internal validation cohort for TabPFN, random forest, XGBoost, and LightGBM. (B) Confusion matrix in the internal validation cohort after prespecified Hungarian alignment of the independently reclustered reference labels on cluster-profile distances. (C) Confusion matrix in the derivation cohort. Rows indicate reference phenotypes and columns indicate predicted phenotypes; cell values are patient counts. (D) One-vs-rest precision–recall curves for Clusters 1–3 in internal validation. (E) Phenotype-specific calibration curves and Brier scores in internal validation. The 10-bin multiclass ECE is shown separately in Supplementary Figure 20D.

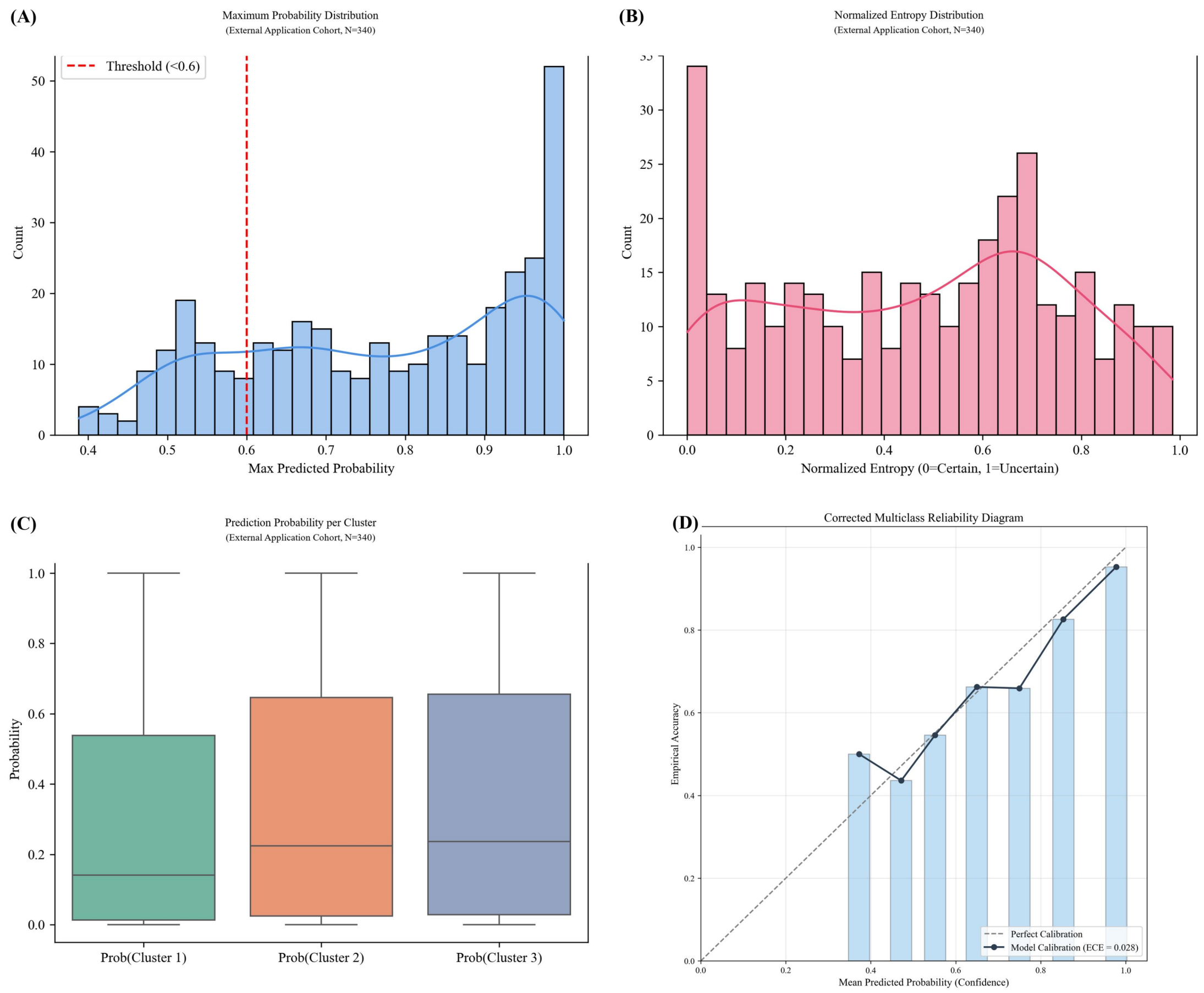

Supplementary Figure 20. External prediction uncertainty and internal-validation multiclass calibration

(A) Distribution of maximum predicted phenotype probability in the external application cohort; the dashed line at 0.60 is a descriptive low-confidence threshold rather than a validated rejection rule. (B) Distribution of normalized entropy, where 0 indicates greater certainty and 1 greater uncertainty. (C) Distributions of predicted probabilities for Clusters 1–3 in the external application cohort. (D) Ten-bin multiclass reliability diagram in the internal validation cohort after prespecified Hungarian alignment of the reference clusters; 10-bin multiclass ECE = 0.028.

ECE, expected calibration error.

Supplementary Tables

Supplementary Table 1. ICD-9-CM and ICD-10-CM codes used for heart failure identification and prespecified disease exclusions

Condition	ICD-9-CM code(s)	ICD-10-CM code(s)	Role
Heart failure	428.0; 428.1; 428.20-428.23; 428.30-428.33; 428.40-428.43; 428.9	I50.1; I50.20-I50.23; I50.30-I50.33; I50.40-I50.43; I50.810-I50.814; I50.82-I50.84; I50.89; I50.9	Inclusion
Infiltrative or restrictive cardiomyopathy	425.4	I42.3-I42.5; I43	Exclusion
Hypertrophic cardiomyopathy	425.1; 425.11; 425.18	I42.1-I42.2	Exclusion
Acute myocardial infarction	410.00-410.92	I21.0-I21.9; I22.0-I22.9	Exclusion
Congenital heart disease	745.0-747.9	Q20.0-Q28.9	Exclusion
Constrictive pericarditis	423.2	I31.1	Exclusion
Heart transplant status or history	V42.1	Z94.1	Exclusion

Notes: Heart-failure codes were used together with a documented left ventricular ejection fraction of at least 50% to define HFpEF eligibility. The remaining code groups were used as prespecified exclusion criteria. Codes are displayed with decimal points for readability; MIMIC diagnosis fields were matched after removal of decimal points. ICD-9-CM, International Classification of Diseases, Ninth Revision, Clinical Modification; ICD-10-CM, International Classification of Diseases, Tenth Revision, Clinical Modification.

Supplementary Table 2. Baseline characteristics of the internal validation cohort stratified by clusters

Variables	Phenotypes			*P*-value
	**Cluster 1** N = 425	**Cluster 2** N = 530	**Cluster 3** N = 569	
Sex, n (%)				< 0.001
Female	179 (42)	350 (66)	381 (67)	
Male	246 (58)	180 (34)	188 (33)	
Age, years	76.93 (67.43-84.17)	78.55 (66.82-85.25)	78.42 (70.14-85.25)	0.088
EF, %	55.00 (55.00-60.00)	55.00 (55.00-60.00)	55.00 (55.00-55.00)	< 0.001
Weight, kg	83.30 (70.36-95.70)	81.44 (67.87-98.00)	75.20 (63.50-90.00)	< 0.001
HR, beats/min	78.00 (67.00-91.00)	91.00 (78.00-105.00)	84.00 (75.00-94.00)	< 0.001
SBP, mmHg	125.00 (108.00-144.00)	136.00 (121.00-153.00)	109.79 (98.00-122.00)	< 0.001
MBP, mmHg	74.00 (66.00-84.00)	89.00 (78.00-102.00)	70.00 (62.00-77.00)	< 0.001
RR, breaths/min	19.00 (16.00-23.00)	21.00 (17.00-25.00)	16.00 (14.00-21.00)	< 0.001
Temperature, °C	37.11 (36.72-37.72)	37.17 (36.83-37.72)	37.44 (37.00-38.00)	< 0.001
SpO_2, %	93.00 (90.00-98.00)	94.00 (90.00-97.00)	95.00 (91.00-100.00)	< 0.001
Glucose, mg/dL	149.00 (115.00-195.00)	140.00 (115.00-179.00)	131.00 (110.00-158.00)	< 0.001
Hematocrit, %	30.10 (27.20-33.60)	33.35 (29.90-37.20)	29.30 (26.10-32.50)	< 0.001
MCHC, g/dL	33.00 (32.00-33.90)	33.10 (32.00-34.10)	33.60 (32.60-34.70)	< 0.001
MCV, fL	90.00 (86.00-94.00)	89.00 (84.00-92.00)	91.00 (87.00-95.00)	< 0.001
Platelet, K/μL	217.00 (163.00-272.00)	233.00 (182.00-304.00)	186.00 (132.00-243.00)	< 0.001
RDW, %	15.60 (14.50-16.90)	14.80 (14.00-16.20)	15.00 (14.00-16.10)	< 0.001

Variables	Phenotypes			*P*-value
	**Cluster 1** N = 425	**Cluster 2** N = 530	**Cluster 3** N = 569	
WBC, K/μL	10.20 (7.40-14.50)	10.20 (7.60-13.50)	11.10 (7.80-15.30)	0.025
Anion gap, mEq/L	16.00 (14.00-18.00)	13.00 (11.00-15.00)	12.00 (11.00-14.00)	< 0.001
BUN, mg/dL	51.00 (34.00-73.00)	21.00 (15.00-29.00)	22.00 (17.00-33.00)	< 0.001
Calcium, mg/dL	8.50 (8.04-9.00)	8.60 (8.20-9.00)	8.12 (7.60-8.50)	< 0.001
Chloride, mEq/L	102.00 (98.00-107.00)	102.00 (98.00-105.00)	108.00 (105.00-112.00)	< 0.001
Creatinine, mg/dL	2.30 (1.60-3.60)	1.00 (0.80-1.20)	1.00 (0.80-1.30)	< 0.001
Sodium, mEq/L	138.00 (135.00-141.00)	139.00 (136.00-141.00)	140.00 (138.00-142.00)	< 0.001
Potassium, mEq/L	4.50 (4.00-5.00)	4.00 (3.60-4.40)	4.10 (3.70-4.50)	< 0.001
PT, s	15.00 (13.40-18.78)	14.70 (13.30-18.10)	15.30 (13.70-17.00)	0.470
PTT, s	33.33 (28.5-42.20)	31.90 (27.20-40.54)	34.30 (29.00-42.60)	< 0.001
Ventilation, n (%)				< 0.001
NO	299 (70)	356 (67)	210 (37)	
YES	126 (30)	174 (33)	359 (63)	
Myocardial infarction, n (%)				< 0.001
NO	312 (73)	444 (84)	470 (83)	
YES	113 (27)	86 (16)	99 (17)	
Peripheral vascular disease, n (%)				< 0.001
NO	348 (82)	490 (92)	485 (85)	
YES	77 (18)	40 (7.5)	84 (15)	
Cerebrovascular disease, n (%)				0.120
NO	391 (92)	467 (88)	516 (91)	
YES	34 (8.0)	63 (12)	53 (9.3)	

Variables	Phenotypes			*P*-value
	**Cluster 1** N = 425	**Cluster 2** N = 530	**Cluster 3** N = 569	
Chronic pulmonary disease, n (%)				< 0.001
NO	274 (64)	221 (42)	356 (63)	
YES	151 (36)	309 (58)	213 (37)	
Diabetes, n (%)				< 0.001
NO	138 (32)	360 (68)	405 (71)	
YES	287 (68)	170 (32)	164 (29)	
Liver disease, n (%)				0.087
NO	387 (91)	502 (95)	528 (93)	
YES	38 (8.9)	28 (5.3)	41 (7.2)	
Renal disease, n (%)				< 0.001
NO	86 (20)	477 (90)	449 (79)	
YES	339 (80)	53 (10)	120 (21)	
Malignant cancer, n (%)				0.350
NO	393 (92)	481 (91)	511 (90)	
YES	32 (7.5)	49 (9.2)	58 (10)	
Hypertension, n (%)				< 0.001
NO	375 (88)	167 (32)	315 (55)	
YES	50 (12)	363 (68)	254 (45)	
Cardiac arrhythmias, n (%)				0.300
NO	165 (39)	225 (42)	217 (38)	
YES	260 (61)	305 (58)	352 (62)	
Diuretics, n (%)				0.001
NO	258 (61)	264 (50)	287 (50)	
YES	167 (39)	266 (50)	282 (50)	
Beta-blockers, n (%)				0.110
NO	282 (66)	336 (63)	341 (60)	
YES	143 (34)	194 (37)	228 (40)	
ACEI/ARB, n (%)				< 0.001
NO	381 (90)	444 (84)	525 (92)	
YES	44 (10)	86 (16)	44 (7.7)	

Variables	Phenotypes			*P*-value
	**Cluster 1**<br>N = 425	**Cluster 2**<br>N = 530	**Cluster 3**<br>N = 569	
Status 365, n (%)	149 (35)	162 (31)	161 (28)	0.072

Data are presented as n (%) for categorical variables and as median (interquartile range) for continuous variables. Group comparisons were performed using Pearson's chi-squared test for categorical variables and Kruskal-Wallis rank sum test for continuous variables. A two-sided *P* value < 0.05 was considered statistically significant.

Abbreviations: BUN, blood urea nitrogen; EF, ejection fraction; HR, heart rate; MBP, mean blood pressure; MCHC, mean corpuscular hemoglobin concentration; MCV, mean corpuscular volume; PT, prothrombin time; PTT, partial thromboplastin time; RDW, red cell distribution width; RR, respiratory rate; SBP, systolic blood pressure; SpO_2, peripheral oxygen saturation; WBC, white blood cell count.

Comorbidities were defined using ICD codes. Status 365 denotes all-cause mortality by day 365 after ICU admission; under the 24-hour landmark design, entry into the risk set occurred at 24 hours (excluding patients who died within the first 24 hours), and the maximum post-landmark follow-up was 364 days.

Supplementary Table 3. Baseline characteristics of the external application cohort stratified by classifier-assigned phenotypes

Variables	Phenotypes			*P*-value
	**Cluster 1**<br>**N** = 101	**Cluster 2**<br>**N** = 116	**Cluster 3**<br>**N** = 123	
Sex, n (%)				0.120
Female	49 (49)	41 (35)	55 (45)	
Male	52 (51)	75 (65)	68 (55)	
Age, years	70.00<br>(60.00-76.00)	71.50<br>(62.00-76.00)	72.00<br>(63.00-78.00)	0.520
EF, %	61.30<br>(57.80-65.30)	62.35<br>(59.05-66.10)	61.90<br>(58.70-65.30)	0.370
Weight, kg	65.00<br>(60.92-72.00)	65.71<br>(60.00-74.80)	68.45<br>(62.65-71.41)	0.330
HR, beats/min	97.00<br>(81.00-113.00)	92.50<br>(81.50-109.50)	88.00<br>(77.00-108.00)	0.041
SBP, mmHg	127.00<br>(111.00-142.00)	149.50<br>(133.00-158.50)	117.00<br>(102.00-133.89)	< 0.001
MBP, mmHg	92.33<br>(80.67-100.00)	106.00<br>(98.17-115.33)	85.33<br>(76.00-94.33)	< 0.001
RR, breaths/min	22.00<br>(20.00-25.00)	22.00<br>(20.00-27.00)	20.00<br>(16.00-23.00)	< 0.001
Temperature, ℃	36.60<br>(36.40-37.10)	36.60<br>(36.35-37.05)	36.70<br>(36.30-37.00)	0.760
SpO_2, %	95.00<br>(91.54-98.00)	96.00<br>(93.00-97.84)	96.00<br>(94.00-98.00)	0.150
Glucose, mg/dL	189.20<br>(154.27-236.00)	163.67<br>(130.60-205.40)	171.48<br>(122.50-200.00)	0.014
Platelet, K/µL	161.00<br>(104.00-232.00)	211.33<br>(156.50-284.00)	159.00<br>(111.00-227.00)	< 0.001
Hematocrit, %	31.60<br>(25.80-37.30)	35.05<br>(29.55-39.95)	36.70<br>(31.10-41.10)	< 0.001
MCV, fL	91.50<br>(87.20-95.40)	92.10<br>(88.70-94.55)	94.20<br>(89.90-97.70)	< 0.001
MCHC, g/dL	32.52<br>(31.80-33.30)	32.80<br>(32.10-33.40)	33.00<br>(32.40-33.70)	0.021
WBC, K/µL	10.60<br>(7.13-16.00)	9.55<br>(7.05-13.21)	8.46<br>(6.30-12.50)	0.039

Variables	Phenotypes			*P*-value
	**Cluster 1** N = 101	**Cluster 2** N = 116	**Cluster 3** N = 123	
RDW, %	14.70 (14.00-16.10)	14.50 (13.50-16.17)	14.20 (13.50-15.50)	0.043
Creatinine, mg/dL	2.30 (1.50-3.90)	0.80 (0.70-1.10)	0.80 (0.60-1.10)	< 0.001
BUN, mg/dL	48.10 (31.90-69.70)	19.80 (14.95-28.85)	21.30 (16.20-28.50)	< 0.001
Sodium, mEq/L	138.20 (134.90-142.10)	138.35 (135.75-140.80)	139.40 (135.33-142.30)	0.660
Potassium, mEq/L	4.42 (4.08-5.06)	4.15 (3.83-4.43)	4.17 (3.83-4.59)	< 0.001
Chloride, mEq/L	102.10 (97.10-106.90)	101.55 (97.60-104.90)	103.90 (99.40-106.90)	0.039
Calcium, mg/dL	8.30 (7.80-8.62)	8.40 (8.15-8.80)	8.40 (8.00-8.80)	0.250
Anion gap, mEq/L	17.20 (13.70-20.80)	13.85 (11.97-16.05)	13.20 (11.10-14.80)	< 0.001
PT, s	12.90 (11.80-15.40)	12.58 (11.63-14.35)	12.80 (11.70-14.00)	0.480
PTT, s	30.50 (27.10-35.00)	29.50 (27.2-33.05)	29.20 (27.00-32.90)	0.300
Ventilation, n (%)				0.100
NO	51 (50)	75 (65)	73 (59)	
YES	50 (50)	41 (35)	50 (41)	
Myocardial infarction, n (%)				0.390
NO	92 (91)	110 (95)	117 (95)	
YES	9 (8.9)	6 (5.2)	6 (4.9)	
Peripheral vascular disease, n (%)				0.120
NO	78 (77)	76 (66)	92 (75)	
YES	23 (23)	40 (34)	31 (25)	
Cerebrovascular disease, n (%)				0.920
NO	62 (61)	68 (59)	74 (60)	
YES	39 (39)	48 (41)	49 (40)	

Variables	Phenotypes			*P*-value
	**Cluster 1** **N** = 101	**Cluster 2** **N** = 116	**Cluster 3** **N** = 123	
Chronic pulmonary disease, n (%)				0.260
NO	78 (77)	78 (67)	87 (71)	
YES	23 (23)	38 (33)	36 (29)	
Diabetes, n (%)				< 0.001
NO	27 (27)	69 (59)	90 (73)	
YES	74 (73)	47 (41)	33 (27)	
Liver disease, n (%)				0.089
NO	56 (55)	81 (70)	76 (62)	
YES	45 (45)	35 (30)	47 (38)	
Renal disease, n (%)				< 0.001
NO	7 (6.9)	83 (72)	88 (72)	
YES	94 (93)	33 (28)	35 (28)	
Malignant cancer, n (%)				0.370
NO	95 (94)	105 (91)	109 (89)	
YES	6 (5.9)	11 (9.5)	14 (11)	
Hypertension, n (%)				< 0.001
NO	32 (32)	45 (39)	71 (58)	
YES	69 (68)	71 (61)	52 (42)	
Cardiac arrhythmias, n (%)				0.058
NO	64 (63)	77 (66)	64 (52)	
YES	37 (37)	39 (34)	59 (48)	
Diuretics, n (%)				0.320
NO	74 (73)	74 (64)	85 (69)	
YES	27 (27)	42 (36)	38 (31)	
Beta-blockers, n (%)				0.470
NO	68 (67)	77 (66)	90 (73)	
YES	33 (33)	39 (34)	33 (27)	
ACEI/ARB, n (%)				0.017
NO	77 (76)	84 (72)	107 (87)	
YES	24 (24)	32 (28)	16 (13)	

Variables	Phenotypes			*P*-value
	**Cluster 1** **N** = 101	**Cluster 2** **N** = 116	**Cluster 3** **N** = 123	
Status 365, n (%)	43 (43)	44 (38)	40 (33)	0.300

Data are presented as n (%) for categorical variables and as median (interquartile range) for continuous variables. Group comparisons were performed using Pearson's chi-squared test for categorical variables and Kruskal-Wallis rank sum test for continuous variables. All *P* values are two-sided. Laboratory measurements were harmonized across cohorts before analysis; MCHC is reported in g/dL in all tables. Comorbidities were identified using prespecified ICD-9/ICD-10 codes. Status 365 denotes all-cause mortality by day 365 after ICU admission; under the 24-hour landmark design, entry into the risk set occurred at 24 hours (excluding patients who died within the first 24 hours), and the maximum post-landmark follow-up was 364 days. Abbreviations: BUN, blood urea nitrogen; EF, ejection fraction; HR, heart rate; MBP, mean blood pressure; MCHC, mean corpuscular hemoglobin concentration; MCV, mean corpuscular volume; PT, prothrombin time; PTT, partial thromboplastin time; RDW, red cell distribution width; RR, respiratory rate; SBP, systolic blood pressure; SpO_2, peripheral oxygen saturation; WBC, white blood cell count.

Supplementary Table 4. Unified propensity-score overlap, event-count, effective-sample-size, summary balance, and covariate-specific ASMD diagnostics

A. Derivation cohort

Medication	Stratum	Exposed events/N	Unexposed events/N	PS overlap (%)	sIPTW ESS (E/U)	OW ESS (E/U)	Max. pre-ASMD	Max. post-sIPTW ASMD (n > 0.10)	Max. post-OW ASMD (n > 0.10)
Diuretics	Overall	387/1311	381/1200	99.6	1230.2/1106.8	1218.1/1124.7	0.231	0.011 (0)	0.000 (0)
Diuretics	1	138/375	143/358	99.0	347.0/322.2	345.0/326.9	0.300	0.021 (0)	0.000 (0)
Diuretics	2	167/511	123/411	99.6	495.1/389.9	485.3/399.2	0.264	0.013 (0)	0.000 (0)
Diuretics	3	82/425	115/431	99.5	393.6/396.6	390.7/400.9	0.397	0.018 (0)	0.000 (0)
ACEI/ARB	Overall	60/290	708/2221	99.0	216.5/2191.1	284.1/1390.2	0.548	0.128 (2)	0.000 (0)
ACEI/ARB	1	13/68	268/665	98.9	51.5/660.7	67.4/432.9	0.601	0.169 (4)	0.000 (0)
ACEI/ARB	2	37/151	253/771	98.0	114.2/755.3	146.9/548.6	0.560	0.135 (3)	0.000 (0)
ACEI/ARB	3	10/71	187/785	92.2	59.5/780.5	70.5/495.0	0.498	0.240 (4)	0.000 (0)
Beta-blockers	Overall	292/1035	476/1476	99.6	946.7/1406.1	982.9/1349.5	0.230	0.015 (0)	0.000 (0)
Beta-blockers	1	104/299	177/434	98.9	266.2/405.1	277.2/378.9	0.388	0.042 (0)	0.000 (0)
Beta-blockers	2	126/411	164/511	98.5	379.0/487.8	390.8/476.6	0.261	0.018 (0)	0.000 (0)
Beta-blockers	3	62/325	135/531	99.1	304.9/517.4	317.0/499.4	0.182	0.023 (0)	0.000 (0)

B. Internal validation cohort

Medication	Stratum	Exposed events/N	Unexposed events/N	PS overlap (%)	sIPTW ESS (E/U)	OW ESS (E/U)	Max. pre-ASMD	Max. post-sIPTW ASMD (n > 0.10)	Max. post-OW ASMD (n > 0.10)
Diuretics	Overall	203/715	269/809	99.7	638.5/743.6	653.8/734.2	0.269	0.018 (0)	0.000 (0)
Diuretics	1	62/167	87/258	99.3	152.6/247.0	160.4/233.4	0.373	0.036 (0)	0.000 (0)
Diuretics	2	80/266	82/264	98.7	250.2/244.8	247.8/248.3	0.323	0.019 (0)	0.000 (0)
Diuretics	3	61/282	100/287	99.0	242.4/259.3	249.1/258.0	0.378	0.033 (0)	0.000 (0)
ACEI/ARB	Overall	38/174	434/1350	98.5	127.1/1330.3	170.3/800.3	0.464	0.140 (4)	0.000 (0)
ACEI/ARB	1	12/44	137/381	94.8	28.5/372.0	42.5/166.0	0.730	0.357 (6)	0.000 (0)
ACEI/ARB	2	20/86	142/444	98.7	65.1/437.9	84.4/336.2	0.457	0.075 (0)	0.000 (0)
ACEI/ARB	3	6/44	155/525	85.6	38.7/523.0	43.9/360.5	0.480	0.261 (6)	0.000 (0)
Beta-blockers	Overall	158/565	314/959	99.3	483.4/906.9	534.6/832.0	0.168	0.029 (0)	0.000 (0)
Beta-blockers	1	43/143	106/282	97.9	118.9/266.7	134.1/232.9	0.409	0.047 (0)	0.000 (0)
Beta-blockers	2	60/194	102/336	98.3	169.2/321.6	185.6/297.3	0.239	0.040 (0)	0.000 (0)
Beta-blockers	3	55/228	106/341	99.3	198.1/320.7	215.5/304.1	0.328	0.027 (0)	0.000 (0)

C. External application cohort

Medication	Stratum	Exposed events/N	Unexposed events/N	PS overlap (%)	sIPTW ESS (E/U)	OW ESS (E/U)	Max. pre-ASMD	Max. post-sIPTW ASMD (n > 0.10)	Max. post-OW ASMD (n > 0.10)
Diuretics	Overall	27 / 107	100 / 233	90.0	92.3/225.3	102.3/200.0	0.298	0.074 (0)	0.000 (0)
Diuretics	1	7 / 27	36 / 74	81.2	23.7/71.8	26.2/60.0	0.478	0.391 (10)	0.383 (7)
Diuretics	2	12 / 42	32 / 74	86.2	37.7/72.1	39.8/67.1	0.418	0.237 (6)	0.228 (4)
Diuretics	3	8 / 38	32 / 85	95.1	33.5/82.0	36.8/74.3	0.370	0.253 (7)	0.220 (6)
ACEI/ARB	Overall	25 / 72	102 / 268	90.3	51.3/256.6	69.7/185.1	0.678	0.103 (1)	0.000 (0)
ACEI/ARB	1	9 / 24	34 / 77	80.2	18.8/75.2	23.5/58.3	0.458	0.418 (9)	0.401 (9)
ACEI/ARB	2	12 / 32	32 / 84	90.5	21.2/78.2	30.7/60.2	0.618	0.257 (12)	0.293 (8)
ACEI/ARB	3	4 / 16	36 / 107	81.3	12.1/105.6	15.6/76.2	0.819	0.646 (9)	0.489 (11)
Beta-blockers	Overall	36 / 105	91 / 235	97.3	92.7/226.9	101.1/204.8	0.220	0.037 (0)	0.000 (0)
Beta-blockers	1	11 / 33	32 / 68	94.1	28.9/65.8	31.8/56.8	0.398	0.247 (4)	0.248 (6)
Beta-blockers	2	13 / 39	31 / 77	94.8	34.9/73.4	37.9/68.3	0.367	0.426 (10)	0.452 (8)
Beta-blockers	3	12 / 33	28 / 90	87.8	29.6/88.6	31.6/81.5	0.650	0.505 (8)	0.460 (7)

D. Covariate-specific ASMDs: derivation cohort

D1. Diuretics

Covariate	Overall			Cluster 1			Cluster 2			Cluster 3		
	Pre	sIPTW	OW	Pre	sIPTW	OW	Pre	sIPTW	OW	Pre	sIPTW	OW
Age	**0.115**	0.003	0.000	0.025	0.021	0.000	**0.139**	0.003	0.000	**0.207**	0.008	0.000
Sex	**0.135**	0.004	0.000	**0.300**	0.002	0.000	0.074	0.002	0.000	0.037	0.006	0.000
Weight	**0.231**	0.002	0.000	0.031	0.013	0.000	**0.264**	0.003	0.000	**0.397**	0.018	0.000
Heart rate	0.010	0.004	0.000	0.058	0.002	0.000	0.023	0.004	0.000	0.018	0.007	0.000
SBP	0.052	0.006	0.000	**0.152**	0.004	0.000	0.008	0.000	0.000	0.079	0.012	0.000
SpO_2	0.100	0.005	0.000	**0.166**	0.016	0.000	**0.166**	0.007	0.000	**0.189**	0.008	0.000
Log creatinine	**0.123**	0.011	0.000	**0.293**	0.016	0.000	0.018	0.004	0.000	**0.108**	0.001	0.000
Potassium	**0.112**	0.002	0.000	0.006	0.011	0.000	**0.123**	0.013	0.000	**0.270**	0.013	0.000
Log BUN	0.016	0.007	0.000	0.035	0.015	0.000	0.052	0.005	0.000	**0.104**	0.017	0.000
Myocardial infarction	0.010	0.001	0.000	0.056	0.001	0.000	0.039	0.001	0.000	0.036	0.004	0.000
Chronic pulmonary disease	**0.133**	0.001	0.000	**0.140**	0.003	0.000	0.031	0.001	0.000	**0.170**	0.004	0.000
Renal disease	0.029	0.003	0.000	0.072	0.002	0.000	0.061	0.001	0.000	0.045	0.009	0.000
Hypertension	0.039	0.003	0.000	0.027	0.014	0.000	0.058	0.003	0.000	0.002	0.003	0.000
Cardiac arrhythmias	0.012	0.005	0.000	0.002	0.014	0.000	0.037	0.004	0.000	0.016	0.005	0.000

D2. ACEI/ARB

Covariate	Overall			Cluster 1			Cluster 2			Cluster 3		
	Pre	sIPTW	OW	Pre	sIPTW	OW	Pre	sIPTW	OW	Pre	sIPTW	OW
Age	0.022	0.005	0.000	**0.124**	0.012	0.000	**0.140**	0.020	0.000	**0.137**	0.051	0.000
Sex	0.039	0.006	0.000	0.017	0.033	0.000	0.014	0.011	0.000	0.077	0.061	0.000
Weight	0.078	0.054	0.000	0.053	0.050	0.000	0.091	0.002	0.000	**0.189**	**0.152**	0.000
Heart rate	0.071	0.039	0.000	0.084	0.073	0.000	**0.223**	0.002	0.000	**0.454**	**0.240**	0.000
SBP	**0.548**	**0.104**	0.000	**0.352**	0.019	0.000	**0.560**	**0.102**	0.000	**0.498**	**0.140**	0.000
SpO_2	0.093	0.073	0.000	**0.193**	0.030	0.000	0.021	**0.112**	0.000	0.035	0.010	0.000
Log creatinine	**0.232**	0.041	0.000	**0.547**	**0.169**	0.000	0.075	0.005	0.000	0.064	0.094	0.000
Potassium	**0.174**	0.013	0.000	0.079	0.028	0.000	**0.179**	0.019	0.000	**0.132**	0.020	0.000
Log BUN	**0.327**	0.030	0.000	**0.601**	**0.141**	0.000	**0.287**	0.016	0.000	0.015	0.058	0.000
Myocardial infarction	0.015	0.030	0.000	0.061	**0.122**	0.000	0.000	0.018	0.000	**0.107**	0.061	0.000
Chronic pulmonary disease	0.001	0.036	0.000	**0.177**	0.013	0.000	0.070	0.030	0.000	**0.108**	0.012	0.000
Renal disease	0.082	0.013	0.000	0.002	0.090	0.000	0.046	**0.135**	0.000	0.035	0.017	0.000
Hypertension	**0.298**	**0.128**	0.000	0.025	**0.164**	0.000	**0.291**	0.043	0.000	**0.369**	**0.214**	0.000
Cardiac arrhythmias	**0.182**	0.058	0.000	0.091	0.072	0.000	**0.329**	0.030	0.000	0.039	0.078	0.000

D3. Beta-blockers

Covariate	Overall			Cluster 1			Cluster 2			Cluster 3		
	Pre	sIPTW	OW	Pre	sIPTW	OW	Pre	sIPTW	OW	Pre	sIPTW	OW
Age	0.045	0.004	0.000	0.006	0.002	0.000	**0.122**	0.011	0.000	0.009	0.005	0.000
Sex	0.050	0.005	0.000	**0.150**	0.017	0.000	0.030	0.001	0.000	0.040	0.008	0.000
Weight	0.042	0.014	0.000	**0.169**	0.031	0.000	0.085	0.018	0.000	**0.125**	0.009	0.000
Heart rate	**0.211**	0.005	0.000	**0.136**	0.010	0.000	**0.229**	0.001	0.000	**0.166**	0.000	0.000
SBP	**0.230**	0.015	0.000	**0.388**	0.042	0.000	**0.158**	0.008	0.000	0.080	0.005	0.000
SpO_2	0.000	0.001	0.000	**0.110**	0.011	0.000	0.027	0.008	0.000	0.087	0.009	0.000
Log creatinine	0.041	0.010	0.000	0.020	0.020	0.000	0.017	0.007	0.000	**0.119**	0.008	0.000
Potassium	0.058	0.006	0.000	0.054	0.014	0.000	**0.213**	0.003	0.000	**0.134**	0.005	0.000
Log BUN	0.060	0.014	0.000	0.091	0.020	0.000	0.017	0.006	0.000	**0.112**	0.012	0.000
Myocardial infarction	**0.132**	0.007	0.000	**0.315**	0.020	0.000	0.005	0.017	0.000	**0.115**	0.011	0.000
Chronic pulmonary disease	0.096	0.007	0.000	**0.172**	0.034	0.000	**0.159**	0.002	0.000	0.067	0.003	0.000
Renal disease	0.025	0.007	0.000	**0.278**	0.041	0.000	0.018	0.005	0.000	0.056	0.014	0.000
Hypertension	0.092	0.007	0.000	**0.205**	0.034	0.000	**0.155**	0.003	0.000	**0.182**	0.012	0.000
Cardiac arrhythmias	**0.107**	0.007	0.000	0.041	0.005	0.000	**0.261**	0.003	0.000	0.085	0.023	0.000

E. Covariate-specific ASMDs: internal validation cohort

E1. Diuretics

Covariate	Overall			Cluster 1			Cluster 2			Cluster 3		
	Pre	sIPTW	OW	Pre	sIPTW	OW	Pre	sIPTW	OW	Pre	sIPTW	OW
Age	0.036	0.003	0.000	0.081	0.005	0.000	0.092	0.002	0.000	0.083	0.003	0.000
Sex	0.081	0.000	0.000	0.073	0.008	0.000	**0.117**	0.019	0.000	0.057	0.033	0.000
Weight	**0.204**	0.001	0.000	0.021	0.021	0.000	**0.323**	0.006	0.000	**0.247**	0.021	0.000
Heart rate	0.000	0.006	0.000	**0.102**	0.004	0.000	**0.103**	0.015	0.000	**0.151**	0.002	0.000
SBP	0.006	0.008	0.000	**0.116**	0.001	0.000	**0.168**	0.002	0.000	**0.126**	0.009	0.000
SpO_2	**0.130**	0.002	0.000	0.021	0.002	0.000	0.056	0.001	0.000	**0.378**	0.001	0.000
Log creatinine	**0.269**	0.018	0.000	**0.373**	0.036	0.000	0.016	0.005	0.000	**0.211**	0.003	0.000
Potassium	**0.104**	0.005	0.000	0.051	0.015	0.000	**0.211**	0.009	0.000	**0.340**	0.006	0.000
Log BUN	**0.177**	0.008	0.000	0.071	0.013	0.000	0.088	0.003	0.000	**0.242**	0.017	0.000
Myocardial infarction	0.048	0.007	0.000	**0.169**	0.005	0.000	0.017	0.006	0.000	0.036	0.026	0.000
Chronic pulmonary disease	**0.129**	0.002	0.000	**0.117**	0.010	0.000	**0.121**	0.001	0.000	0.094	0.002	0.000
Renal disease	**0.102**	0.012	0.000	0.005	0.013	0.000	0.040	0.007	0.000	0.078	0.001	0.000
Hypertension	**0.165**	0.012	0.000	0.082	0.002	0.000	0.078	0.012	0.000	**0.201**	0.013	0.000
Cardiac arrhythmias	0.090	0.010	0.000	0.003	0.009	0.000	**0.152**	0.007	0.000	**0.109**	0.026	0.000

E2. ACEI/ARB

Covariate	Overall			Cluster 1			Cluster 2			Cluster 3		
	Pre	sIPTW	OW	Pre	sIPTW	OW	Pre	sIPTW	OW	Pre	sIPTW	OW
Age	0.091	0.038	0.000	**0.174**	0.081	0.000	**0.144**	0.004	0.000	**0.177**	**0.160**	0.000
Sex	0.001	0.004	0.000	0.075	0.022	0.000	0.023	0.011	0.000	0.076	0.043	0.000
Weight	0.012	0.025	0.000	**0.320**	0.035	0.000	0.004	0.018	0.000	**0.185**	0.069	0.000
Heart rate	**0.319**	0.076	0.000	**0.229**	**0.115**	0.000	**0.457**	0.001	0.000	**0.480**	**0.261**	0.000
SBP	**0.464**	**0.140**	0.000	**0.576**	**0.357**	0.000	**0.270**	0.053	0.000	**0.229**	0.045	0.000
SpO_2	0.034	0.043	0.000	**0.133**	0.064	0.000	0.039	0.014	0.000	0.078	0.092	0.000
Log creatinine	**0.216**	**0.104**	0.000	**0.360**	0.022	0.000	**0.146**	0.075	0.000	**0.130**	0.081	0.000
Potassium	**0.106**	0.075	0.000	0.044	**0.224**	0.000	**0.141**	0.013	0.000	0.023	**0.140**	0.000
Log BUN	**0.264**	**0.132**	0.000	**0.730**	**0.149**	0.000	0.002	0.023	0.000	**0.184**	**0.149**	0.000
Myocardial infarction	**0.275**	0.046	0.000	**0.399**	0.039	0.000	**0.249**	0.028	0.000	**0.261**	**0.160**	0.000
Chronic pulmonary disease	0.050	0.006	0.000	**0.316**	**0.108**	0.000	0.088	0.047	0.000	0.024	0.038	0.000
Renal disease	**0.147**	0.047	0.000	0.068	0.031	0.000	**0.128**	0.069	0.000	0.079	0.012	0.000
Hypertension	**0.220**	**0.113**	0.000	0.063	0.063	0.000	0.063	0.006	0.000	**0.317**	**0.132**	0.000
Cardiac arrhythmias	**0.141**	0.033	0.000	**0.253**	**0.121**	0.000	0.098	0.039	0.000	0.062	0.044	0.000

E3. Beta-blockers

Covariate	Overall			Cluster 1			Cluster 2			Cluster 3		
	Pre	sIPTW	OW	Pre	sIPTW	OW	Pre	sIPTW	OW	Pre	sIPTW	OW
Age	0.014	0.029	0.000	0.084	0.027	0.000	0.022	0.040	0.000	0.020	0.016	0.000
Sex	0.084	0.006	0.000	**0.123**	0.014	0.000	**0.172**	0.017	0.000	0.072	0.005	0.000
Weight	0.021	0.028	0.000	**0.113**	0.039	0.000	0.036	0.023	0.000	0.017	0.027	0.000
Heart rate	**0.155**	0.005	0.000	**0.279**	0.045	0.000	**0.202**	0.011	0.000	0.005	0.013	0.000
SBP	**0.147**	0.001	0.000	**0.317**	0.004	0.000	**0.146**	0.005	0.000	**0.159**	0.002	0.000
SpO_2	**0.130**	0.016	0.000	**0.181**	0.006	0.000	**0.112**	0.014	0.000	**0.328**	0.027	0.000
Log creatinine	**0.129**	0.000	0.000	0.084	0.008	0.000	**0.110**	0.021	0.000	**0.273**	0.011	0.000
Potassium	0.021	0.003	0.000	0.027	0.014	0.000	0.082	0.000	0.000	**0.246**	0.006	0.000
Log BUN	**0.144**	0.008	0.000	**0.154**	0.028	0.000	**0.151**	0.023	0.000	**0.328**	0.007	0.000
Myocardial infarction	0.095	0.012	0.000	**0.212**	0.011	0.000	**0.120**	0.017	0.000	0.006	0.012	0.000
Chronic pulmonary disease	**0.168**	0.007	0.000	**0.409**	0.047	0.000	**0.216**	0.004	0.000	0.025	0.016	0.000
Renal disease	0.011	0.001	0.000	**0.105**	0.026	0.000	0.096	0.004	0.000	0.019	0.001	0.000
Hypertension	**0.129**	0.011	0.000	0.038	0.015	0.000	**0.108**	0.013	0.000	**0.165**	0.001	0.000
Cardiac arrhythmias	**0.139**	0.014	0.000	0.010	0.016	0.000	**0.239**	0.022	0.000	**0.151**	0.005	0.000

F. Covariate-specific ASMDs: external application cohort (descriptive)

F1. Diuretics

Covariate	Overall			Cluster 1			Cluster 2			Cluster 3		
	Pre	sIPTW	OW	Pre	sIPTW	OW	Pre	sIPTW	OW	Pre	sIPTW	OW
Age	**0.242**	0.024	0.000	0.049	**0.334**	**0.313**	**0.398**	**0.159**	**0.139**	**0.297**	0.077	0.098
Sex	0.010	0.029	0.000	**0.319**	**0.285**	**0.259**	0.090	0.077	0.041	**0.231**	0.093	**0.158**
Weight	**0.276**	0.074	0.000	**0.343**	**0.228**	0.094	**0.290**	0.013	0.013	**0.218**	0.003	0.081
Heart rate	0.036	0.010	0.000	0.053	0.043	0.036	0.075	**0.108**	0.084	0.034	0.085	0.082
SBP	**0.120**	0.005	0.000	**0.141**	0.066	0.070	0.057	0.075	0.079	**0.118**	0.059	0.029
SpO_2	0.089	0.016	0.000	**0.148**	**0.139**	**0.112**	0.065	0.039	0.052	0.068	**0.104**	0.027
Log creatinine	**0.298**	0.039	0.000	**0.478**	**0.154**	0.100	**0.125**	0.008	0.045	**0.207**	**0.131**	0.068
Potassium	**0.294**	0.055	0.000	**0.419**	**0.112**	0.062	**0.189**	**0.121**	0.056	**0.180**	0.019	0.078
Log BUN	**0.213**	0.013	0.000	**0.403**	**0.120**	**0.128**	**0.127**	**0.237**	**0.228**	**0.202**	**0.253**	**0.198**
Myocardial infarction	0.035	0.001	0.000	0.074	0.055	0.003	0.029	0.077	0.047	0.026	0.001	0.032
Chronic pulmonary disease	**0.253**	0.019	0.000	**0.218**	0.018	0.088	**0.418**	**0.191**	**0.192**	0.073	**0.111**	**0.126**
Renal disease	**0.192**	0.000	0.000	**0.188**	**0.282**	**0.304**	0.079	0.008	0.000	**0.337**	**0.248**	**0.220**
Hypertension	0.043	0.017	0.000	**0.262**	**0.391**	**0.335**	0.099	0.094	0.079	**0.226**	**0.155**	**0.128**
Cardiac arrhythmias	**0.209**	0.033	0.000	**0.202**	**0.314**	**0.383**	**0.386**	**0.214**	**0.176**	**0.370**	**0.189**	**0.136**

F2. ACEI/ARB

Covariate	Overall			Cluster 1			Cluster 2			Cluster 3		
	Pre	sIPTW	OW	Pre	sIPTW	OW	Pre	sIPTW	OW	Pre	sIPTW	OW
Age	**0.104**	0.071	0.000	0.055	**0.321**	**0.299**	**0.357**	**0.114**	**0.293**	0.046	0.001	0.072
Sex	0.025	0.004	0.000	**0.294**	**0.367**	**0.401**	0.028	**0.167**	0.031	**0.418**	**0.646**	**0.489**
Weight	0.046	0.053	0.000	**0.366**	**0.347**	**0.292**	0.014	**0.154**	0.098	**0.428**	**0.484**	**0.362**
Heart rate	0.018	0.011	0.000	**0.165**	**0.154**	0.092	0.034	**0.166**	**0.109**	**0.107**	0.095	**0.113**
SBP	**0.401**	**0.103**	0.000	**0.318**	**0.168**	0.005	**0.301**	0.006	0.032	**0.192**	0.079	0.100
SpO_2	0.034	0.023	0.000	**0.182**	**0.178**	**0.161**	**0.260**	**0.257**	**0.243**	0.056	**0.208**	**0.153**
Log creatinine	0.079	0.064	0.000	0.024	0.052	**0.109**	**0.199**	**0.236**	0.010	**0.327**	**0.367**	**0.454**
Potassium	0.056	0.009	0.000	0.012	**0.132**	**0.123**	0.056	0.028	0.030	**0.297**	**0.255**	**0.356**
Log BUN	0.095	0.054	0.000	**0.227**	0.068	0.015	0.012	**0.178**	**0.150**	**0.431**	**0.512**	**0.380**
Myocardial infarction	0.040	0.034	0.000	**0.156**	0.067	**0.109**	**0.136**	**0.238**	**0.211**	0.069	0.046	**0.102**
Chronic pulmonary disease	0.021	0.042	0.000	0.069	**0.260**	0.011	**0.138**	**0.243**	**0.183**	**0.477**	**0.532**	**0.384**
Renal disease	**0.238**	0.027	0.000	**0.447**	**0.418**	**0.356**	**0.179**	**0.240**	**0.236**	**0.224**	0.090	0.032
Hypertension	**0.678**	0.062	0.000	**0.458**	0.050	**0.127**	**0.618**	**0.123**	0.070	**0.819**	**0.289**	**0.197**
Cardiac arrhythmias	**0.168**	0.053	0.000	**0.208**	0.065	0.010	**0.113**	**0.222**	**0.259**	**0.399**	**0.311**	**0.366**

F3. Beta-blockers

Covariate	Overall			Cluster 1			Cluster 2			Cluster 3		
	Pre	sIPTW	OW	Pre	sIPTW	OW	Pre	sIPTW	OW	Pre	sIPTW	OW
Age	**0.184**	0.024	0.000	**0.167**	0.061	**0.123**	0.055	**0.103**	**0.101**	**0.398**	**0.292**	**0.265**
Sex	0.034	0.006	0.000	0.089	0.075	0.035	0.098	0.100	0.064	0.063	0.023	0.061
Weight	0.080	0.011	0.000	**0.320**	**0.247**	**0.248**	**0.361**	**0.426**	**0.452**	**0.269**	**0.156**	**0.202**
Heart rate	**0.193**	0.008	0.000	**0.123**	0.048	0.032	0.072	**0.162**	**0.137**	**0.331**	**0.175**	**0.121**
SBP	0.043	0.020	0.000	0.009	0.047	0.037	0.042	0.021	0.049	0.082	**0.152**	0.091
SpO_2	**0.188**	0.020	0.000	**0.127**	0.006	0.013	**0.282**	**0.140**	**0.155**	**0.175**	0.042	0.094
Log creatinine	**0.214**	0.022	0.000	**0.254**	0.061	0.030	**0.158**	0.062	0.077	**0.650**	**0.505**	**0.460**
Potassium	0.096	0.007	0.000	0.099	0.028	0.010	0.087	**0.112**	0.076	0.087	0.043	0.047
Log BUN	0.082	0.015	0.000	0.073	0.001	0.030	0.006	**0.111**	0.066	**0.332**	**0.357**	**0.340**
Myocardial infarction	**0.138**	0.002	0.000	0.009	**0.185**	**0.231**	**0.163**	0.060	0.057	**0.240**	**0.114**	**0.150**
Chronic pulmonary disease	0.001	0.007	0.000	**0.157**	**0.175**	**0.137**	**0.148**	**0.152**	**0.175**	0.031	0.046	0.097
Renal disease	0.056	0.010	0.000	0.052	0.045	**0.208**	**0.367**	**0.319**	**0.346**	0.056	0.010	0.002
Hypertension	**0.132**	0.037	0.000	**0.143**	0.042	**0.160**	**0.171**	**0.117**	**0.103**	0.004	0.058	0.077
Cardiac arrhythmias	**0.220**	0.030	0.000	**0.398**	**0.156**	0.087	**0.349**	**0.160**	**0.123**	0.097	**0.231**	**0.236**

Notes: Summary panels A–C report values in parentheses after the maximum post-weighting ASMD as the number of covariates with ASMD > 0.10; ESS is reported as exposed/unexposed. Detailed panels D–F report covariate-specific absolute standardized mean differences before weighting and after sIPTW and OW; values > 0.10 are bolded. The 14 prespecified propensity-score covariates were age, sex, weight, heart rate, SBP, SpO_2, creatinine, potassium, BUN, myocardial infarction, chronic pulmonary disease, renal disease, hypertension, and cardiac arrhythmias.

Creatinine and BUN were assessed on the log scale used in the propensity-score models. In the derivation and internal validation cohorts, medication-specific propensity-score models included cluster membership and all cluster-by-covariate interaction terms. In the smaller external application cohort, weights were estimated from cohort-level models without cluster interactions; the resulting cluster-specific values are descriptive balance diagnostics only. OW was the primary exploratory weighted analysis and sIPTW was a sensitivity analysis. ACEI, angiotensin-converting enzyme inhibitor; ARB, angiotensin receptor blocker; ASMD, absolute standardized mean difference; BUN, blood urea nitrogen; ESS, effective sample size; OW, overlap weighting; SBP, systolic blood pressure; sIPTW, stabilized inverse probability of treatment weighting; SpO_2, peripheral oxygen saturation.

Supplementary Table 5. Comparison of the K = 2 and K = 3 clustering solutions in the derivation and internal validation cohorts

Criterion	K = 2 solution	K = 3 solution	Interpretation
Cluster structure	1,040 (41.4%); 1,471 (58.6%)	733 (29.2%); 922 (36.7%); 856 (34.1%)	Both solutions avoided very small clusters; K = 3 yielded three reasonably balanced groups.
Clinical profiles	Broad high-burden group versus an older, lower renal-metabolic-burden group	Cardiorenal-metabolic; hypertensive-pulmonary; low-blood-pressure/arrhythmia profiles	K = 3 separated clinically recognizable patterns that were combined under K = 2.
Derivation-cohort 365-day mortality	36.0% versus 26.8% (*P* < 0.001)	38.3%, 31.5%, and 23.0% (*P* < 0.001)	K = 3 provided a graded descriptive mortality pattern rather than a binary split.
Internal-validation survival separation	Log-rank *P* < 0.0001	Log-rank *P* = 0.072	Prognostic separation was sensitive to clustering resolution.
Mean silhouette width	0.083	0.060	Both values were low; K = 2 showed modestly greater separation.
Resampling stability	Median ARI 0.940 (IQR 0.260)	Median ARI 0.924 (IQR 0.175)	Both solutions showed high median stability.
OW medication-by-cluster interaction *P* values	Derivation: 0.992/0.421/0.234; validation: 0.297/0.838/0.967	Derivation: 0.816/0.387/0.750; validation: 0.192/0.567/0.565	No interaction was identified under either K.
Overall assessment	Statistically more compact; retained as an equally plausible lower-resolution sensitivity solution	Greater clinical resolution; retained as the main exploratory representation	K = 3 was clinically motivated and does not establish statistical or biological superiority.

Notes: Medication interaction *P* values are ordered as diuretics/ACEI/ARB/beta-blockers. ARI, adjusted Rand index; OW, overlap weighting.

Supplementary Table 6. Missingness and distribution differences in the 14 phenotype-classifier variables between the derivation and external application cohorts

Feature	Derivation missing (%)	External missing (%)	Derivation mean ± SD	External mean ± SD	ASMD	*P* value
MBP	<0.1	0.88	82.00 ± 17.99	78.91 ± 17.07	0.176	< 0.001
Creatinine	1.23	9.41	1.63 ± 1.36	1.63 ± 1.35	0.000	0.913
BUN	1.31	10.29	33.37 ± 22.26	33.67 ± 22.88	0.013	0.680
RR	<0.1	0.88	19.96 ± 5.84	19.54 ± 5.82	0.072	0.029
Chloride	1.23	0	102.55 ± 6.41	104.04 ± 6.40	0.233	< 0.001
SBP	0.52	0.88	126.51 ± 25.72	125.26 ± 25.74	0.049	0.134
HR	<0.1	0.88	85.82 ± 19.31	86.60 ± 19.58	0.040	0.220
Renal Disease	<0.1	0	0.41 ± 0.49	0.34 ± 0.47	0.146	<0.001
Hematocrit	2.7	0	31.47 ± 6.10	31.27 ± 5.40	0.035	0.280
Glucose	0.71	22.35	151.39 ± 67.86	154.11 ± 66.26	0.041	0.210
Calcium	8.8	0	8.44 ± 0.68	8.38 ± 0.76	0.083	0.010
SpO_2	<0.1	19.41	96.50 ± 3.73	94.25 ± 4.41	0.551	<0.001
Anion gap	1.43	0	14.60 ± 3.70	13.97 ± 3.32	0.179	<0.001
Platelet	3.78	0	204.67 ± 88.81	224.06 ± 98.79	0.206	<0.001

Notes: Missingness was summarized before imputation. ASMD was calculated from the reported cohort means and standard deviations; values > 0.10 indicate a potentially meaningful distribution difference. *P* values are descriptive and

are not used as a criterion for model transportability. ASMD, absolute standardized mean difference; SD, standard deviation.

TabPFN robustness and representative-case analyses

Supplementary Table 7. Feature-ablation performance of the TabPFN phenotype classifier

Feature removed	Macro-F1	Delta Macro-F1	Phenotype 1 AUC	Delta AUC	Phenotype 2 AUC	Delta AUC	Phenotype 3 AUC	Delta AUC
Hematocrit	0.798	0.0320	0.963	0.0057	0.948	0.0134	0.945	0.0063
Respiratory rate	0.804	0.0253	0.968	0.0011	0.948	0.0130	0.932	0.0186
Glucose	0.806	0.0240	0.966	0.0035	0.954	0.0068	0.949	0.0017
Renal disease	0.806	0.0232	0.950	0.0190	0.956	0.0054	0.944	0.0070
Heart rate	0.812	0.0179	0.972	-0.0031	0.952	0.0091	0.942	0.0088
Peripheral oxygen saturation	0.814	0.0156	0.970	-0.0005	0.953	0.0076	0.944	0.0071
Anion gap	0.817	0.0123	0.968	0.0015	0.961	0.0004	0.947	0.0036
Calcium	0.817	0.0122	0.968	0.0009	0.958	0.0032	0.947	0.0036
Blood urea nitrogen	0.822	0.0081	0.968	0.0010	0.958	0.0029	0.949	0.0021
Creatinine	0.822	0.0079	0.963	0.0057	0.955	0.0060	0.950	0.0008
Mean blood pressure	0.828	0.0021	0.972	-0.0026	0.960	0.0008	0.948	0.0031
Chloride	0.828	0.0012	0.977	-0.0079	0.953	0.0084	0.947	0.0037
Systolic blood pressure	0.829	0.0009	0.970	-0.0009	0.959	0.0018	0.950	0.0007
Platelet	0.833	-0.0030	0.968	0.0006	0.958	0.0033	0.949	0.0025

Notes: Delta values were calculated as full-model performance minus performance after removal of the specified feature; positive values indicate performance loss after ablation, whereas negative values indicate a small improvement after ablation. Phenotype-specific AUCs were calculated using a one-versus-rest approach. AUC, area under the receiver operating characteristic curve.

Supplementary Table 8. Representative correctly classified, misclassified, and low-confidence cases from the TabPFN phenotype classifier

A. Classification outcomes and predicted probabilities

**Case category**	**Sample index**	**Reference phenotype**	**Predicted phenotype**	**Predicted probabilities (P1 / P2 / P3)**	**Maximum probability**
Correctly classified case 1	13	2	2	0.089 / 0.810 / 0.101	0.810
Correctly classified case 2	6	2	2	0.034 / 0.912 / 0.053	0.912
Misclassified case 1	1	1	3	0.029 / 0.055 / 0.916	0.916
Misclassified case 2	3	1	2	0.435 / 0.562 / 0.003	0.562
Low-confidence case	1493	2	3	0.251 / 0.373 / 0.376	0.376

B. Raw feature profiles of the representative cases

**Feature**	**Correct case 1**	**Correct case 2**	**Misclassified case 1**	**Misclassified case 2**	**Low-confidence case**
Mean blood pressure, mmHg	78.33	51	73.67	69	60
Creatinine, mg/dL	1.0	1.1	1.1	1.2	1.9
Blood urea nitrogen, mg/dL	35	24	27	29	48
Respiratory rate, breaths/min	37	19	16	24	27
Chloride, mEq/L	97	92	104	94	108
Systolic blood pressure, mmHg	99	91	107	121	114
Heart rate, beats/min	111	63	64	89	87
Renal disease (0/1)	0	0	0	1	0
Hematocrit, %	20.5	45.579	31.9	33.1	31.1
Glucose, mg/dL	134	145	159	149	125
Calcium, mg/dL	6.4	9.4	8.5	7.9	8.1
Peripheral oxygen saturation, %	90	89	90	90	93
Anion gap, mEq/L	17	12	11	17	15
Platelet, K/µL	40	227	162	228	152

Notes: P1, P2, and P3 denote Phenotypes 1, 2, and 3, respectively. The predicted phenotype corresponds to the largest predicted probability. Continuous feature values are raw values reported in the source analysis output; renal disease is a binary indicator coded as 0 or 1. Sample index corresponds to the index reported in the source analysis output. BUN, blood urea nitrogen; SpO_2, peripheral oxygen saturation.

Supplementary Table 9. Scaled Schoenfeld residual tests for the full medication-by-cluster Cox models in the derivation and internal validation cohorts

A. Derivation cohort

Medication	Model	Medication main effect chi-square (df); P	Cluster main effect chi-square (df); P	Medication-by-cluster chi-square (df); P	Global test chi-square (df); P
Diuretics	Unadjusted	2.163 (1); 0.141	2.618 (2); 0.270	1.261 (2); 0.532	7.585 (5); 0.181
Diuretics	sIPTW	2.360 (1); 0.124	4.342 (2); 0.114	1.775 (2); 0.412	9.703 (5); 0.084
Diuretics	OW	1.178 (1); 0.278	2.130 (2); 0.345	0.931 (2); 0.628	4.672 (5); 0.457
ACEI/ARB	Unadjusted	1.404 (1); 0.236	2.455 (2); 0.293	2.873 (2); 0.238	6.051 (5); 0.301
ACEI/ARB	sIPTW	0.008 (1); 0.931	2.842 (2); 0.241	0.368 (2); 0.832	3.693 (5); 0.594
ACEI/ARB	OW	0.206 (1); 0.650	0.867 (2); 0.648	1.048 (2); 0.592	1.642 (5); 0.896
Beta-blockers	Unadjusted	**4.867 (1); 0.027**	2.571 (2); 0.277	4.624 (2); 0.099	8.079 (5); 0.152
Beta-blockers	sIPTW	**6.223 (1); 0.013**	3.301 (2); 0.192	5.741 (2); 0.057	9.815 (5); 0.081
Beta-blockers	OW	2.783 (1); 0.095	1.453 (2); 0.484	2.932 (2); 0.231	4.396 (5); 0.494

B. Internal validation cohort

Medication	Model	Medication main effect chi-square (df); *P*	Cluster main effect chi-square (df); *P*	Medication-by-cluster chi-square (df); *P*	Global test chi-square (df); *P*
Diuretics	Unadjusted	1.800 (1); 0.180	0.041 (2); 0.980	0.791 (2); 0.674	3.657 (5); 0.600
Diuretics	sIPTW	1.278 (1); 0.258	0.255 (2); 0.880	1.170 (2); 0.557	4.151 (5); 0.528
Diuretics	OW	0.759 (1); 0.384	0.161 (2); 0.923	0.554 (2); 0.758	2.222 (5); 0.818
ACEI/ARB	Unadjusted	0.019 (1); 0.890	0.079 (2); 0.961	0.856 (2); 0.652	1.520 (5); 0.911
ACEI/ARB	sIPTW	0.307 (1); 0.579	0.023 (2); 0.988	1.697 (2); 0.428	3.872 (5); 0.568
ACEI/ARB	OW	0.234 (1); 0.628	0.311 (2); 0.856	0.695 (2); 0.706	1.872 (5); 0.867
Beta-blockers	Unadjusted	0.874 (1); 0.350	0.078 (2); 0.962	2.827 (2); 0.243	4.699 (5); 0.454
Beta-blockers	sIPTW	1.165 (1); 0.280	0.032 (2); 0.984	3.318 (2); 0.190	4.954 (5); 0.422
Beta-blockers	OW	0.604 (1); 0.437	0.086 (2); 0.958	1.868 (2); 0.393	2.405 (5); 0.791

Notes: Each cell reports chi-square (degrees of freedom); *P* value from a scaled Schoenfeld residual test applied to the full-cohort Cox model containing medication, three-level cluster, and medication-by-cluster interaction terms. The diagnostic models used the same survival time, event indicator, complete-case analysis set, and analytical weights as the corresponding analysis. Because an interaction is present, the medication main-effect test is conditional on the reference cluster. *P* values < 0.05 are bolded. In the derivation cohort, the beta-blocker medication term yielded *P* = 0.027 in the descriptive unadjusted model and *P* = 0.013 in the sIPTW sensitivity model; the sIPTW interaction and global tests were *P* = 0.057 and *P* = 0.081, respectively. This term-specific sensitivity-model signal precludes interpreting its coefficient as a constant effect over follow-up; it was not used as confirmatory evidence. No OW test in either cohort and no weighted test in internal validation had *P* < 0.05. ACEI, angiotensin-converting enzyme inhibitor; ARB, angiotensin receptor blocker; OW, overlap weighting; sIPTW, stabilized inverse probability of treatment weighting.
